# AI-assisted identification of disability patterns within identical EDSS grades

**DOI:** 10.1177/13524585251327300

**Published:** 2025-04-18

**Authors:** Martina Greselin, Po-Jui Lu, Magdalena Mroczek, Nuria Cerdá-Fuertes, Anastasios Demirtzoglou, Athina Papadopoulou, Jens Kuhle, David Leppert, Sophie Arnould, Manar Aoun, Ludwig Kappos, Cristina Granziera, Marcus D’Souza

**Affiliations:** Translational Imaging in Neurology (ThINk) Basel, Department of Biomedical Engineering, Faculty of Medicine, University Hospital Basel and University of Basel, Basel, Switzerland; Department of Neurology, University Hospital Basel, Basel, Switzerland; Research Center for Clinical Neuroimmunology and Neuroscience Basel (RC2NB), University Hospital Basel and University of Basel, Basel, Switzerland; Translational Imaging in Neurology (ThINk) Basel, Department of Biomedical Engineering, Faculty of Medicine, University Hospital Basel and University of Basel, Basel, Switzerland; Department of Neurology, University Hospital Basel, Basel, Switzerland; Research Center for Clinical Neuroimmunology and Neuroscience Basel (RC2NB), University Hospital Basel and University of Basel, Basel, Switzerland; Department of Neurology, University Hospital Basel, Basel, Switzerland; Research Center for Clinical Neuroimmunology and Neuroscience Basel (RC2NB), University Hospital Basel and University of Basel, Basel, Switzerland; Translational Imaging in Neurology (ThINk) Basel, Department of Biomedical Engineering, Faculty of Medicine, University Hospital Basel and University of Basel, Basel, Switzerland; Department of Neurology, University Hospital Basel, Basel, Switzerland; Research Center for Clinical Neuroimmunology and Neuroscience Basel (RC2NB), University Hospital Basel and University of Basel, Basel, Switzerland; Department of Neurology, University Hospital Basel, Basel, Switzerland; Research Center for Clinical Neuroimmunology and Neuroscience Basel (RC2NB), University Hospital Basel and University of Basel, Basel, Switzerland; Translational Imaging in Neurology (ThINk) Basel, Department of Biomedical Engineering, Faculty of Medicine, University Hospital Basel and University of Basel, Basel, Switzerland; Department of Neurology, University Hospital Basel, Basel, Switzerland; Research Center for Clinical Neuroimmunology and Neuroscience Basel (RC2NB), University Hospital Basel and University of Basel, Basel, Switzerland; Department of Neurology, University Hospital Basel, Basel, Switzerland; Research Center for Clinical Neuroimmunology and Neuroscience Basel (RC2NB), University Hospital Basel and University of Basel, Basel, Switzerland; Research Center for Clinical Neuroimmunology and Neuroscience Basel (RC2NB), University Hospital Basel and University of Basel, Basel, Switzerland; Novartis AG, Basel, Switzerland; Novartis AG, Basel, Switzerland; Department of Neurology, University Hospital Basel, Basel, Switzerland; Research Center for Clinical Neuroimmunology and Neuroscience Basel (RC2NB), University Hospital Basel and University of Basel, Basel, Switzerland; Translational Imaging in Neurology (ThINk) Basel, Department of Biomedical Engineering, Faculty of Medicine, University Hospital Basel and University of Basel, Basel, Switzerland; Department of Neurology, University Hospital Basel, Basel, Switzerland; Research Center for Clinical Neuroimmunology and Neuroscience Basel (RC2NB), University Hospital Basel and University of Basel, Basel, Switzerland; Department of Neurology, University Hospital Basel, Basel, Switzerland; Research Center for Clinical Neuroimmunology and Neuroscience Basel (RC2NB), University Hospital Basel and University of Basel, Basel, Switzerland

**Keywords:** Multiple sclerosis, EDSS, disability patterns, artificial intelligence, clinical trials

## Abstract

**Background:**

The Neurostatus-Expanded Disability Status Scale (EDSS) is the most frequently used measure of disability in multiple sclerosis (MS) trials. However, EDSS scores ⩾4.5 are mainly based on ambulation and may fail to capture relevant disability patterns in other functional domains.

**Objective:**

The objective was to determine how assessments categorized with the same EDSS score may reflect distinct disability patterns.

**Methods:**

We analysed 13,103 assessments from 1636 people with secondary progressive MS, from the EXPAND trial. The data set is composed of Functional System scores (FSS) and their corresponding subscores, Ambulation scores and EDSS scores. We performed a descriptive analysis to define the relevant Functional Systems (FS). The subscores were then binarized based on the Neurostatus definition and grouped by respective EDSS scores. Finally, we applied two consecutive machine learning algorithms, to cluster the data. New subscore patterns were then created by aggregating clusters based on their dominant features.

**Results:**

The clustering algorithm yielded numerous clusters, grouping assessments with similar patterns. In patients with EDSS ⩾4.0, our approach allowed differentiation into four subscore patterns within the same EDSS score.

**Conclusion:**

Applying Artificial Intelligence (AI) to large data sets of high-quality clinical assessments allows for distinguishing among different subscore patterns within identical EDSS scores.

## Introduction

Multiple sclerosis (MS) is a chronic, immune-mediated disease of the central nervous system, manifesting clinically by acute episodes of inflammatory activity (relapses) and continuous deterioration of neurological functions. The latter might result from relapse-associated worsening (RAW) or – more frequently – from progression independent of relapse activity (PIRA).^1,2^

Clinical history and neurological examination remain the gold standard for quantifying and tracking disability over time. The Expanded Disability Status Scale (EDSS) is the clinical reference measure for assessing clinical changes and is used to define confirmed worsening or confirmed progression for both, RAW and PIRA. Furthermore, the EDSS serves as the main clinical reference to be compared with other measures, such as magnetic resonance imaging (MRI), fluid biomarkers^3,4^ and patient-reported outcomes,^5^ cross-sectionally, longitudinally or regarding their prognostic value.^6^

However, an EDSS score ⩾4.5 reflects almost exclusively the ambulation capacity, while the deficits captured by other Functional Systems (FS) and their respective subscores tend to be eclipsed. This loss of information has important implications for documenting relevant disease worsening and understanding disease phenotypes of people with multiple sclerosis (pwMS). Such neglect of deficits in domains other than ambulation is also an obstacle to patient-tailored disease management.

The Neurostatus-EDSS is the main measure in phase 2 and 3 trials for new disease-modifying treatments in MS.^7^ It summarizes 120 subscores (=quantified items of the neurological examination) based on a standardized neurological examination into seven Functional System Scores (FSS) and one Ambulation Score (AS), ultimately determining the respective EDSS score.^8^ In 2009, the Neurostatus-EDSS paper version was supplemented by a digital version, the Neurostatus-eEDSS.^9,10^ The latter provides automated, algorithm-based real-time feedback on the consistency of entries, resulting in higher accuracy and reduced noise in assessments. In regulatory-relevant studies, real-time feedback is complemented by an expert review provided by the University Hospital Basel to resolve remaining inconsistencies.^10,11^

Our objective was to investigate how far identical EDSS scores harbour heterogeneous patterns of disability. We applied machine learning algorithms (MLA) to the detailed Neurostatus-eEDSS assessments obtained during the EXPAND study in secondary progressive MS (SPMS).^12^ By integrating various subscores from each assessment, the MLA identified patterns within the data and clusters with similar characteristics. The results of the clustering algorithms are used to create a sub-classification, offering complementary information to the respective EDSS scores for a more comprehensive reflection of overall disability in pwMS.

## Materials and methods

**Figure 1.**
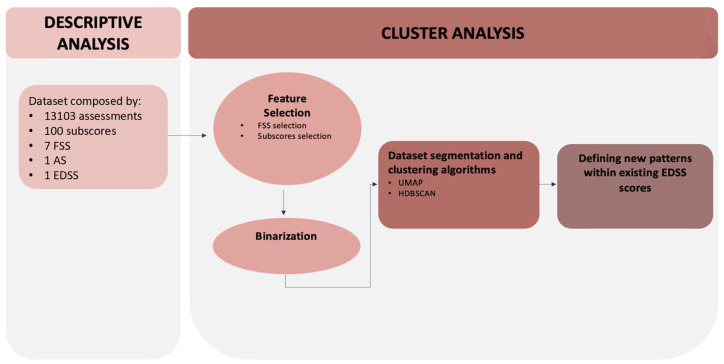
Flowchart outlining the key steps of the analysis, with references to the corresponding chapters in the manuscript. FSS: Functional System Score; EDSS: Expanded Disability Status Scale; AS: Ambulation Score; UMAP: Uniform Manifold Approximation and Projection; HDBSCAN: Hierarchical Density-Based Spatial Clustering of Applications with Noise.

A flowchart of the key steps of our study is presented in Figure 1.

### Data set and pre-processing

The data set was derived from the EXPAND trial a double-blinded, randomized, phase 3 study, where 1651 people with secondary progressive MS (pwSPMS) were randomly assigned to receive Siponimod or placebo.^12^ The placebo-controlled core part was stopped after the occurrence of ⩾374 confirmed disability worsening (CDW) events. At this time, ⩾95% of participants had been under assigned treatment for ⩾12 months.^12^ The Neurostatus-eEDSS was obtained every 3 months by certified raters, capturing the scoring results on a tablet that provided real-time feedback, followed with an expert-review interaction.^11^ Our data set consists of all mandatory subscores (100) per EDSS assessment, FSS (7), AS (1) and the EDSS score (1) from every visit attended. It also includes labels ‘P’ (for permanent deficits that are not due to MS) and ‘T’ (for temporary not MS-related deficits), sex and age. No information about the treatment arm was included in our data set. All data were encrypted, and the study was approved by the Ethics Committee of North-western and Central Switzerland (ID: 2017-02099).

Encrypted data were transferred via dedicated channels to ensure confidentiality and integrity. The initial visit is considered the baseline visit. We adjusted the data set for the occurrence of ‘P’ and ‘T’ and replaced the values of these subscores with ‘normal’ values of a standardized clinical examination, according to the Neurostatus-EDSS definitions.^8^

### Descriptive analysis

We conducted a descriptive analysis to examine the distribution of FSS according to the EDSS scores in the data set.

### Cluster analysis

Assessments with identical EDSS score, ranging between 4.0 and 6.5, were used to investigate their different patterns of subscores, using cluster analysis, as described in detail below.

#### Feature selection

The boxplots obtained in the descriptive analysis were utilized to identify the FS exhibiting the highest values in the data set. Subscores associated with these FS were chosen through a dual criterion approach using the Pearson correlation coefficient and the subscore distribution. Specifically, subscores with a Pearson correlation coefficient greater than 0.70 with another subscore or demonstrating a high uniformity in assessment values (where over 85% of the assessments had the same value) were excluded from the analysis.

#### Binarization

**Figure 2.**
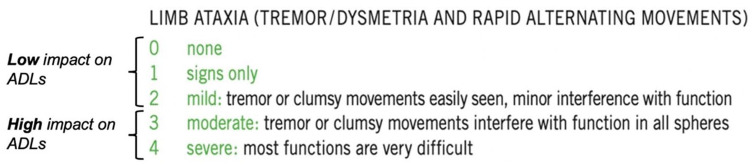
Example of the binarization for tremor dysmetria and rapid alternating movements subscores. Binarization groups the scores into two categories: those considered having a low impact on ADLs and those considered having a medium to high impact on ADLs. In this case, the threshold was set at 2: all grades equal to or lower than ‘mild’ were classified as ‘zero’, and all values higher than ‘mild’ were classified as ‘one’.

The data set exhibits complexity, wherein each subscore encompasses multiple potential grades (0 to 5). Consequently, we employed a method which we defined as ‘binarization’, categorizing grades based on the Neurostatus-EDSS definitions. Each subscore was transformed into two possible values: a ‘zero’, including subscore gradings of ‘none’, ‘signs only’ and ‘mild’ and a ‘one’, including those subscore gradings that per Neurostatus definition imply a higher impact on daily life activities (‘moderate’ and ‘severe’/‘marked’). For muscle strength we applied an additional step before binarization, details of which are reported in the Supplementary Materials. For subscores involving information from both right and left limbs, a single value was computed for both sides of the body. The binarized value corresponds to ‘zero’ if both sides have values equal to or lower than the threshold described above, a value of ‘one’ is assigned in the other cases. Figure 2 shows an example of the limb ataxia subscore, where for each score the severity of the symptoms is reported with a clinical explanation. The threshold for all the binarized subscores is reported in the Supplementary Materials (section 1). The subscores obtained after binarization are referred to as ‘features’.

#### Data set segmentation

The full data set was divided into six data sets, each corresponding to a specific EDSS score ranging from 4.0 to 6.5, in increments of 0.5. The methods outlined in sections ‘Clustering algorithm’ and ‘Defining new patterns within existing EDSS scores’ were applied in two ways:

1. To the entire data set, which includes all assessments with the same EDSS score.
2. For each data set, consisting of assessments with the same EDSS score, a three-fold cross-validation was performed. The data set was split into three equal parts (folds), where in each iteration, one fold served as the validation data set, and the remaining two folds were combined to form the training data set. This process was repeated three times, ensuring that each fold was used once as the validation set. The two MLA algorithms and hyperparameter tuning (as described in sections ‘Clustering algorithm’ and ‘Defining new patterns within existing EDSS scores’) were executed on the training data set for each iteration, and the trained model was subsequently applied to the corresponding validation data set.

#### Clustering algorithm

Two MLAs were applied to capture data-specific patterns of the data set, emphasizing specific characteristics of different assessments. The first, Uniform Manifold Approximation and Projection (UMAP), performed dimensionality reduction and the second, Hierarchical Density-Based Spatial Clustering of Applications with Noise (HDBSCAN), was applied for clustering. UMAP is designed for nonlinear dimensionality reduction of data sets.^13^ It is particularly effective with high-dimensional data and yields clear visualizations and interpretable outcomes.^14^ Moreover, an advantage of this method lies in its ability to improve the performance of clustering algorithms.^15^ Furthermore, it can depict the specific details or the overall patterns in the data set. It excels particularly in efficiently capturing the specific details or local structures within the data.^16^

The UMAP algorithm allows to reduce the dimensionality of the data set to 3 or 2 to facilitate the visualization of results. In this context, a dense region in the low-dimensional space corresponds to data points sharing common features in the starting configuration. Accordingly, the HDBSCAN algorithm emerges as a suitable clustering algorithm that aims at delineating clusters in low-dimensionality data.^17^ The HDBSCAN algorithm is designed to identify clusters in a data set based on the density of data points.^18^ Furthermore, it explicitly identifies noise points in the data that do not belong to any cluster. This is beneficial for handling noisy data.

The relative validity score, a simplification of the Density-Based Cluster Validity (DBCV) score, is applied to compare the results during the tuning of hyperparameters.^19^ The score is calculated with all the combinations of UMAP and HDBSCAN hyperparameters, and the hyperparameter with the higher score is used to calculate the results. Consequently, the results of the clustering algorithm consolidate data exhibiting comparable features.

#### Defining new patterns within existing EDSS scores

For each cluster, we calculated the percentage of assessments with high impairment in activities of daily living (ADLs) for each selected feature (=subscore after binarization). A percentage over 50% indicated that most assessments within the cluster exhibit significant impairment in ADL for that feature. By combining these features, we aggregated the clusters into four subgrades of identical EDSS scores. This categorization groups clusters based on the combination of features that show high impairment in most of the assessments.

## Results

### Descriptive analysis

Following pre-processing, 13,103 assessments obtained from a total of 1636 patients, averaging 8 ± 2.97 visits per patient, were included in our study. Table 1 summarizes the characteristics of the data set.

**Table 1.** Characteristics in our data set.

Number of assessments	13,103
Number of patients	1636
Age (mean ±SD, year)	48 ± 7.9
Sex, female %	60%
Number of visits per patient (mean ± SD)	8 ±2.97
Patients with 6-month CDW, %	342/1636, 21%

CDW: confirmed disability worsening; SD: standard deviation.

The sex and the age are calculated per patient. The CDW was evaluated based on the standard definition, where an increase of at least 1 point on the EDSS was considered for scores between 1.0 and 5.0, and an increase of at least 0.5 points was applied for baseline scores of 5.5 and above.

**Figure 3.**
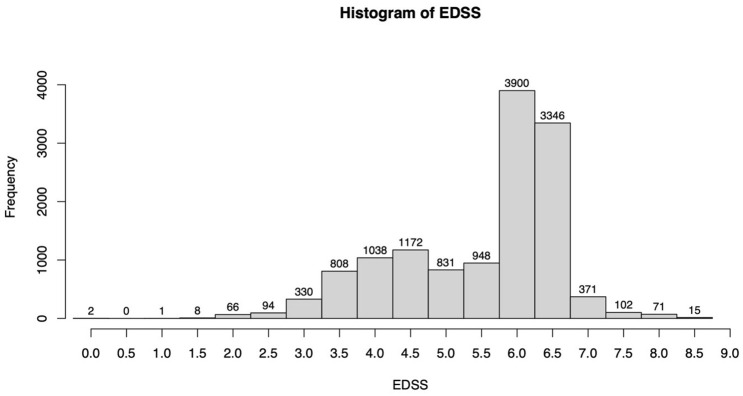
Histogram of the EDSS score distribution in the data set.

The range of the EDSS scores analysed was between 0 and 8.5. Scores of 6.0 and 6.5 showed the highest frequency, while 86% of the assessments ranged between 4.0 and 6.5 (Figure 3). The number of assessments for EDSS scores 0–3.5 and ⩾7.0 was too low for a meaningful statistical evaluation, and hence, further analysis was restricted to EDSS scores from 4.0 to 6.5.

The distribution of FSS, as well as that of subscores for each EDSS score, is shown in the Supplementary Materials (sections 5 and 6).

### Clustering analysis

#### Feature selection

**Figure 4.**
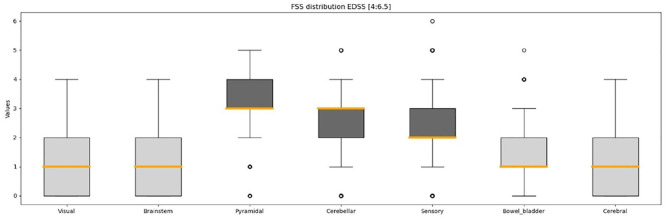
Distribution of Functional Systems for EDSS scores between 4.0 and 6.5. The respective converted values of the Visual FSS and the Bowel and Bladder FSS are utilized. The boxplot’s horizontal line denotes the range between minimum and maximum values. The vertical boundaries of the box delineate the upper and lower quantiles, while the central thick line indicates the median value. The points report the value of the outliers. FSS: Functional System Score.

Within EDSS 4.0–6.5, the Pyramidal, Cerebellar and Sensory FS had the highest FSS values among the seven FS as previously reported by others;^20^ the Visual, Brainstem, Bowel and Bladder, and Cerebral FS were not used in the further analysis due to their lower disability levels in most assessments (Figure 4). Table 2 provides a summary of observed subscores and the application of the defined criterion for feature exclusion. After feature exclusion, the data set was composed of 15 subscores derived from three FS: Pyramidal, Cerebellar and Sensory.

**Table 2.** Subscores of the cerebellar, pyramidal and sensory functional systems, along with their inclusion and exclusion criteria.

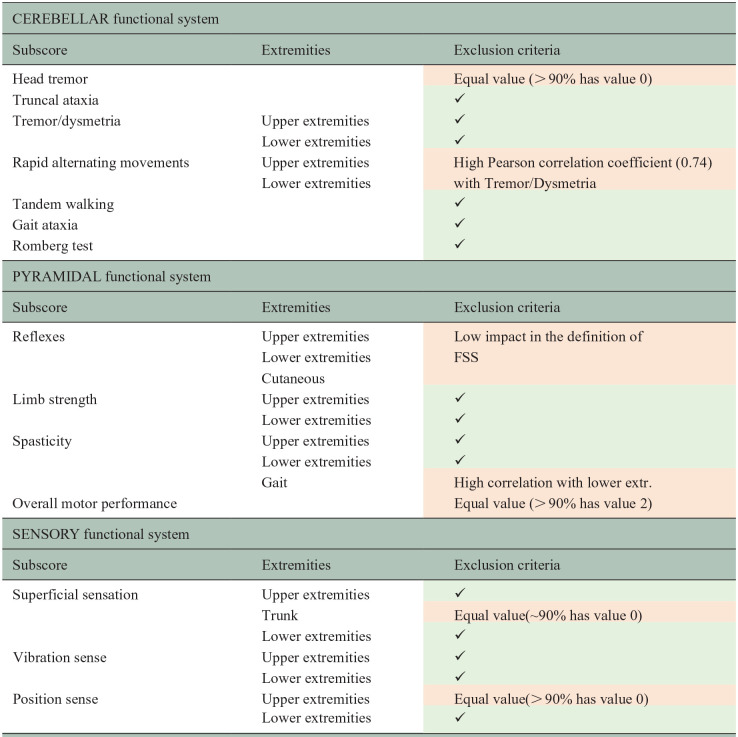

Exclusion criteria are highlighted in red, while the green section with the ✔ symbol indicates the subscores that are included.

#### Data set segmentation

The data set was partitioned into six subsets covering the EDSS scores 4.0–6.5 (Table 3). In total, 11,235 assessments from 1558 patients were used for further analysis.

**Table 3.** The number of assessments and patients for each data set included in the analysis.

EDSS	Number of assessments	Number of patients
4.0	1038	278
4.5	1172	339
5.0	831	275
5.5	948	310
6.0	3900	815
6.5	3346	700

Each data set was then divided into three equal-dimension folders.

#### Cluster algorithms

**Figure 5.**
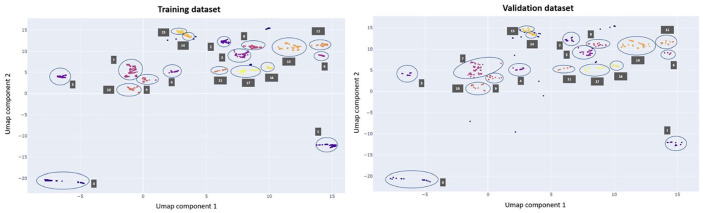
Results from a single cross-validation iteration of the EDSS score 6.0 data set are shown. The plot on the left displays the results for the training data set, while the plot on the right shows the results for the validation data set. The clusters are visually represented by distinct colours, with circles and corresponding labels added to enhance clarity. Outliers are denoted by blue points without accompanying labels. The two components are new simplified dimensions that represent combinations of features to capture important patterns in the data.

Cluster analysis using UMAP and HDBSCAN revealed distinct clusters, as exemplified in Figure 5. Figure 5 presents a cluster analysis of the data set with an EDSS score of 6.0, showing the results from one cross-validation iteration, including both the training and validation data sets.

The data are represented by two components defined by the UMAP algorithm that maintains the data variability. Assessments that are represented as closely positioned data points along the two UMAP components exhibit similar characteristics. Furthermore, the figure demonstrates the separation of the output cluster of the HDBSCAN algorithm by the two components.

The aggregated assessments within clusters exhibit similar characteristics. To confirm this, we calculated the percentage of assessments showing high impairment in the ADL for each cluster. Table 4 provides an example of the cluster composition for EDSS 6.0 for the training data set (full table in Supplemental Materials), indicating that Cluster 0 is characterized by a low percentage of assessments with high impairment in the ADL across all features. In contrast, Cluster 1 is composed entirely of assessments (100%) with high disability only in tandem walking. This shows that assessments within the same cluster share similar characteristics, while different clusters exhibit distinct properties from one another. Figure 5 also illustrates that the results from the training and validation data sets were similar. This similarity in data representation, observed after hyperparameter tuning of UMAP and HDBSCAN, suggests that the selected parameters generalize effectively across data sets.

**Table 4.** Results of the clustering algorithm for the first two clusters of training data sets of a single cross-validation iteration of the EDSS score 6.0.

Number of clusters	BMRC		Spasticity		Truncal ataxia	Tremor		Tandem walking	Gait ataxia	Romberg test	Superficial Sensation		Vibration Sense		Position sense	Number data	Pattern score
	Upper	Lower	Upper	Lower		Upper	Lower				Upper	Lower	Upper	Lower	Lower		
0	9	33	1	0	0	0	1	0	0	3	1	8	1	19	8	203	D
1	1	0	1	0	1	2	7	98	0	9	2	9	6	20	10	183	B

The values represent the percentage of assessments within clusters exhibiting high levels of impairment in ADL. The NUMBER CLUSTER column denotes the cluster identified by a number, while the PATTERN SCORE represents the pattern score assigned to each cluster, as defined in section ‘Defining new patterns within existing EDSS scores’.

#### Defining new patterns within existing EDSS scores

As shown in Table 5, four distinct subscore patterns were identified within each EDSS score level across 4.0 to 6.5. Pattern A was defined if any of the following features was high (cluster contained more than 50% of assessments with a high impact on ADL): muscle strength, tremor/dysmetria, truncal ataxia or gait ataxia. Pattern B was defined if spasticity or tandem walking had high values. Pattern C was applicable if only one of the sensory features or the Romberg test was high. Pattern D was defined as if none of these features was high.

**Table 5.** Composition of the disability patterns detected in identical EDSS scores.

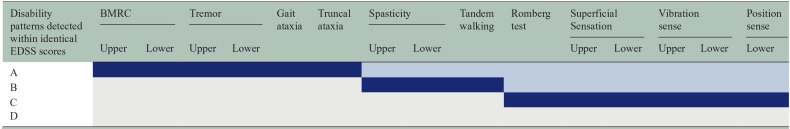

BMRC: British Medical Research Council.

For each row, the corresponding disability pattern is defined if at least one of the dark blue features has a percentage of assessment with high impairment in ADL higher than 50%. The light blue feature’s percentage of high impairment may vary and is not required to be high. For example, to be classified as pattern score B, the dark blue features must be high, while the light blue features can be either high or not.

In the example presented in Table 4, Cluster 0 is classified under pattern D, as it shows <50% assessments with high ADL for all features. In contrast, Cluster 1 exhibits >50% of assessments with high ADL only for tandem walking and is therefore classified under pattern B. The distribution of patterns obtained by each EDSS score after running the procedure across all EDSS scores from 4.0 to 6.5 is provided in Table 6. As shown in the table, assessments categorized under the same EDSS exhibited different patterns, reflecting different cluster characteristics. Overall, higher EDSS scores show a higher percentage of assessments falling within pattern score A.

**Table 6.** The percentage of assessments for each data set categorized by pattern group.

EDSS	Pattern A			Pattern B			Pattern C			Pattern D		
	*full*	*train*	*val*	*full*	*train*	*val*	*full*	*train*	*val*	*full*	*train*	*val*
4	30.6	31.2	25.7	32.3	31.1	30.3	10.8	10.5	10.7	26.3	27.2	33.2
		33.4	32.1		29.1	31.8		9.2	10.1		28.3	26.0
		26.0	29.7		32.5	35.3		13.0	9.6		28.5	25.4
4.5	51.7	44.9	41.3	22.6	27.0	28.5	5.5	4.3	3.4	20.1	23.9	26.9
		51.0	48.3		23.7	25.7		5.1	4.3		20.2	21.7
		48.4	53.4		20.0	21.0		8.8	7.8		22.7	17.8
5	53.2	44.2	50.2	20.4	27.0	20.9	6.0	5.7	0.0	20.4	23.1	28.9
		49.5	52.6		22.5	25.9		5.9	5.6		22.2	15.9
		25.6	62.6		49.3	9.6		0.0	0.0		25.2	27.8
5.5	49.9	52.6	51.6	31.7	29.8	32.2	5.6	3.6	2.8	12.8	14.0	13.5
		47.6	47.9		33.1	34.1		5.7	6.2		13.6	11.8
		57.4	56.2		28.0	26.3		0.0	0.0		14.6	17.5
6	75.4	74.2	73.6	20.0	14.5	14.6	0.0	6.6	7.5	4.6	4.7	4.3
		73.0	71.7		18.6	19.0		0.0	0.0		8.4	9.2
		75.2	75.4		16.6	16.8		0.0	0.0		8.2	7.8
6.5	97.5	90.4	91.0	2.5	9.6	9.0	0.0	0.0	0.0	0.0	0.0	0.0
		96.6	97.7		3.4	2.3		0.0	0.0		0.0	0.0
		91.7	91.3		8.3	8.7		0.0	0.0		0.0	0.0

For eachDSS data set, the results are derived using either the entire data set (full) or by dividing it into training (train) and validation (val) subsets. For each EDSS value, three results were obtained from the cross-validation process, corresponding to the three iterations of the cross-validation.

However, there were 4.6% and 20% of the assessments classified with an EDSS of 6.0 displayed patterns D and B, respectively. For assessments with an EDSS of 6.5, 2.5% of the assessments had pattern scores B (Table 6). These assessments had high EDSS due to ambulatory problems but demonstrated low subscores in other domains.

Table 6 also allows for a comparison of the results obtained when the procedure was applied to the full data set versus when cross-validation was performed. In the cross-validation process, it can be observed that the percentage of assessments belonging to the same pattern group is similar between the training and validation data sets, even though the validation set is treated as new data by the algorithm. Moreover, the results derived from a subset of the data (training dataset) are consistent with those calculated using the full data set. It is important to note that the data set under consideration may include repeatable data due to the limited range of possible values.

## Discussion

The EDSS is the reference scale to quantify disability accumulation in clinical routine and clinical trials for drug development in pwMS and related diseases.^21^ Other than imaging and fluid biomarkers, the EDSS as a clinical descriptor of disability accumulation represents the closest approximation to the subjective burden of disease. This increases its relevance as a pivotal endpoint measure during a drug approval process. The dominant position of the EDSS is further underpinned by being part of composite outcomes, such as No Evidence of Disease Activity (NEDA) and No Evidence of Progression or Active Disease (NEPAD), as well as PIRA and RAW that are used to describe the individual disease status^22,23^ and to discern the different drivers of disease progression in pwMS.^24^

However, a weak point of the EDSS is its dependency on ambulation and its relatively low sensitivity to changes in domains other than ambulation, particularly in more severely affected patients.^25^ Consequently, at EDSS ⩾4.5, the contribution of other clinically relevant symptoms is overshadowed and not reflected by the overall EDSS score alone. An immediate consequence of this is a reduced ability to capture the heterogeneity of the disease and relative inertia to longitudinal change at these EDSS levels, factors of high relevance for the selection of participants and detection of drug effect in clinical trials.^25^

Our study aimed to address these limitations, taking advantage of the large, high-quality data set of the EXPAND study. Using the Neurostatus-eEDSS, this data set contained not only the EDSS and FSS information but also quantified, standardized and rigorously quality-controlled assessments of the full neurological examination, which remarkably includes 100 mandatory subtests per assessment, the so-called subscores.

We applied MLA on those subscores and identified clusters which led to distinct disability patterns within EDSS scores ranging from 4.0 to 6.5. These patterns allow describing with more granularity the range of functional impairment beyond ambulation in pwMS. In our data set, higher EDSS scores include a higher proportion of assessments falling into pattern A. This might indicate that these patterns may also be useful for a more granular quantification of the severity of deficits or further prognosis, but further studies are necessary to explore this option. Such increased granularity may increase the power of clinical trials to detect treatment effects on deficits that are relevant to patients and their ADL but are not reflected by quantifying ambulation only. A more comprehensive description of the burden of disease may help to better select pwMS who may benefit from more specific treatments, both symptomatic and targeted according to underlying pathogenic processes. Further studies in other data sets, including patients at different disease stages, are needed to establish the utility of the patterns derived in this study. If further studies confirm that this increase in granularity comes along with improved correlations to other measures and higher predictive score, these patterns could potentially be integrated into a more granular, ordinally arranged measure, which, while not necessarily equidistant, may serve as an ‘expanded’ version of the EDSS. Such integration of disease patterns would also have implications for improved power of analysis and reduced sample sizes in future clinical trials.

In conclusion, the patterns obtained through our analysis set the stage for further development of the well-established Neurostatus-EDSS and Neurostatus-eEDSS. These advancements aim to provide a more comprehensive assessment of disability, offering greater sensitivity to change and addressing a key limitation of the EDSS, particularly in the severity range between 4.0 and 6.5.

## Supplemental Material

sj-docx-1-msj-10.1177_13524585251327300 – Supplemental material for AI-assisted identification of disability patterns within identical EDSS grades Supplemental material, sj-docx-1-msj-10.1177_13524585251327300 for AI-assisted identification of disability patterns within identical EDSS grades by Martina Greselin, Po-Jui Lu, Magdalena Mroczek, Nuria Cerdá-Fuertes, Anastasios Demirtzoglou, Athina Papadopoulou, Jens Kuhle, David Leppert, Sophie Arnould, Manar Aoun, Ludwig Kappos, Cristina Granziera and Marcus D’Souza in Multiple Sclerosis Journal
