# Supplementary material for: AI-assisted identification of disability patterns within identical EDSS grades
Source: Mult Scler. 2025 Apr 18;31(6):677–88. doi: 10.1177/13524585251327300 (PMC12092942; doi:10.1177/13524585251327300)
Supplement: sj-docx-1-msj-10.1177_13524585251327300 – Supplemental material for AI-assisted identification of disability patterns within identical EDSS grades [file sj-docx-1-msj-10.1177_13524585251327300.docx]

# SUPPLEMENTAL MATERIAL

## BINARIZATION THRESHOLD

The following table reports the binarization threshold for all the subscores.

b) Subscores composed by right and left extremities features.

| **SUBSCORES RIGHT** | **SUBSCORES LEFT** | **Score**  **for low impact on ADL** |
| --- | --- | --- |
| SPASTICITY_ARMS_R | SPASTICITY_ARMS_L | ≤1 |
| SPASTICITY_LEGS_R | SPASTICITY_LEGS_L | ≤1 |
| TREMOR_DYSMETRIA_UE_R | TREMOR_DYSMETRIA_UE_L | ≤2 |
| TREMOR_DYSMETRIA_LE_R | TREMOR_DYSMETRIA_LE_L | ≤2 |
| SUPERFICIAL_SENSATION_UE_R | SUPERFICIAL_SENSATION_UE_L | ≤2 |
| SUPERFICIAL_SENSATION_LE_R | SUPERFICIAL_SENSATION_LE_L | ≤2 |
| VIBRATION_SENSE_UE_R | VIBRATION_SENSE_UE_L | ≤2 |
| VIBRATION_SENSE_LE_R | VIBRATION_SENSE_LE_L | ≤2 |
| POSITION_SENSE_LE_R | POSITION_SENSE_LE_L | ≤1 |

a) Subscores composed by one feature

| **SUBSCORES** | **Score**  **for low impact on ADL** |
| --- | --- |
| BMRC_UE | ≤1 |
| BMRC_LE | ≤1 |
| TRUNCAL_ATAXIA | ≤2 |
| TANDEM_WALKING | ≤1 |
| GAIT_ATAXIA | ≤2 |
| ROMBERG_TEST | ≤2 |

Table S1: Binarization threshold for different subscores. a) Threshold for the subscores with only one feature. The binarized value corresponds to a low impact on ADL if the feature has a value equal to or lower than the threshold. b) Threshold for the subscores with one feature for the right and one for the left extremities. The binarized value corresponded to a low impact on ADL if both the features have values equal to or lower than the threshold, a high impact on daily life was defined in the other cases.

*ADL=Activities of Daily Living; BMRC= British Medical Research Council; UE=Upper Extremities; LE=Lower Extremities; R=Right; L=Left.*

## 2. British Medical Research Council (BMRC) SUBSCORES PREPROCESSING

This feature is composed of 20 subscores, 10 for the upper and 10 for the lower extremities, corresponding to 5 subscores for each extremity. Each of the 20 subscores encompassed 6 disability grades (0 to 5). The six grades were used to calculate a summary score for the lower and upper extremities. Afterwards, these scores were binarized, and the values of high and low disability were obtained also for the BMRC. The approach was based on the specifications and definitions of the Neurostatus-(e)EDSS. An example is provided in Figure S1.


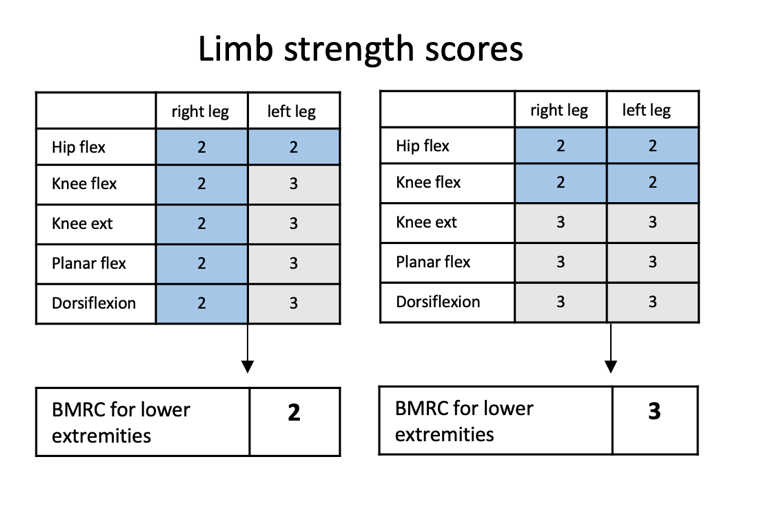


Figure S1: Example of BMRC calculation. Each limb is composed of 5 scores: the value 5 reports normal condition, and the value 0 reports high disability. The values are combined and result in one value for the upper extremities and one for the lower.

### 3. NEW PATTERN SCORE

Table S2 presents the output of the clustering algorithm for the clustering algorithm for training datasets of a single cross-validation iteration of the EDSS score 6.0. The values represent the percentage of assessments within clusters exhibiting high levels of impairments in ADL (= 1 after binarization). Each row corresponds to the results of an individual cluster, with a cluster value of -1 indicating noisy data. Clusters are then aggregated into the pattern score based on features exceeding the 50% threshold, which are highlighted in yellow in Table S2.

| **number_cluster** | **BMRC_upp** | **BMRC_low** | **SPASTICITY_UPP** | **SPASTICITY_LOW** | **TRUNCAL_ATAXIA** | **TREMOR_UPP** | **TREMOR_LOW** | **TANDEM_WALKING** | **GAIT_ATAXIA** | **ROMBERG_TEST** | **SUPERFICIAL_SEN_UPP** | **SUPERFICIAL_SEN_LOW** | **VIBRATION_SEN_UPP** | **VIBRATION_SEN_LOW** | **POSITION_SEN_LOW** | **N_data** | **PATTERN SCORE** |
| --- | --- | --- | --- | --- | --- | --- | --- | --- | --- | --- | --- | --- | --- | --- | --- | --- | --- |
| -1 | 18 | 71 | 4 | 89 | 15 | 25 | 28 | 96 | 63 | 63 | 2 | 16 | 5 | 40 | 14 | 112 | - |
| 0 | 9 | 33 | 1 | 0 | 0 | 0 | 1 | 0 | 0 | 3 | 1 | 8 | 1 | 19 | 8 | 203 | D |
| 1 | 1 | 0 | 1 | 0 | 1 | 2 | 7 | 98 | 0 | 9 | 2 | 9 | 6 | 20 | 10 | 183 | B |
| 2 | 5 | 100 | 11 | 100 | 13 | 8 | 0 | 89 | 100 | 2 | 1 | 7 | 2 | 0 | 9 | 131 | A |
| 3 | 1 | 0 | 14 | 100 | 3 | 3 | 0 | 99 | 100 | 0 | 0 | 5 | 1 | 0 | 8 | 150 | A |
| 4 | 0 | 0 | 5 | 99 | 5 | 28 | 100 | 99 | 100 | 1 | 4 | 6 | 0 | 1 | 11 | 79 | A |
| 5 | 0 | 41 | 8 | 97 | 4 | 5 | 41 | 92 | 99 | 8 | 14 | 24 | 10 | 100 | 32 | 130 | A |
| 6 | 1 | 100 | 15 | 100 | 0 | 0 | 13 | 0 | 0 | 0 | 1 | 1 | 1 | 6 | 9 | 78 | A |
| 7 | 0 | 5 | 3 | 48 | 28 | 18 | 39 | 99 | 99 | 100 | 1 | 6 | 3 | 27 | 6 | 290 | A |
| 8 | 12 | 100 | 22 | 90 | 3 | 22 | 100 | 90 | 98 | 0 | 1 | 11 | 0 | 0 | 6 | 156 | A |
| 9 | 3 | 14 | 0 | 0 | 2 | 16 | 60 | 91 | 92 | 0 | 2 | 1 | 3 | 64 | 0 | 91 | A |
| 10 | 1 | 13 | 1 | 5 | 7 | 10 | 0 | 99 | 93 | 1 | 13 | 15 | 1 | 1 | 2 | 166 | A |
| 11 | 0 | 0 | 1 | 45 | 1 | 3 | 5 | 0 | 100 | 0 | 0 | 3 | 1 | 20 | 5 | 76 | A |
| 12 | 11 | 100 | 22 | 100 | 4 | 2 | 0 | 100 | 0 | 7 | 0 | 2 | 1 | 1 | 9 | 139 | A |
| 13 | 7 | 100 | 11 | 56 | 4 | 1 | 38 | 97 | 2 | 4 | 11 | 16 | 8 | 42 | 12 | 190 | A |
| 14 | 4 | 96 | 11 | 96 | 15 | 43 | 94 | 99 | 96 | 94 | 1 | 7 | 8 | 97 | 61 | 72 | A |
| 15 | 13 | 100 | 14 | 92 | 11 | 17 | 99 | 100 | 89 | 100 | 0 | 2 | 2 | 1 | 5 | 123 | A |
| 16 | 0 | 0 | 0 | 100 | 0 | 0 | 11 | 100 | 0 | 5 | 0 | 0 | 1 | 0 | 4 | 79 | B |
| 17 | 1 | 0 | 29 | 100 | 5 | 1 | 5 | 12 | 1 | 3 | 7 | 13 | 1 | 10 | 7 | 152 | B |

Table S2: The results of the clustering algorithm for the dataset with EDSS equal to 6.0. Each row presents the results of a cluster for each feature, as indicated in the columns. The **NUMBER CLUSTER** column denotes the cluster identified by a number while the **PATTERN SCORE** represents the pattern score assigned to each cluster, as defined in Chapter 3.2.4. UPP=upper extrimities and LOW=Lower extremities.

### 4. FSS Distribution across EDSS steps

The boxplots below illustrates the distribution of FSS across various values of the Expanded Disability Status Scale (EDSS) step. The visual FSS correspond to the converted value of the visual FSS. The same applies to the bowel and bladder measurements.


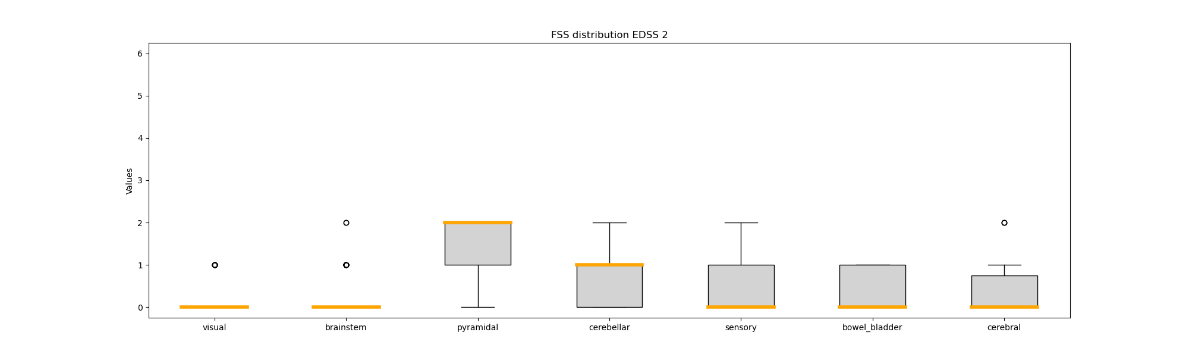


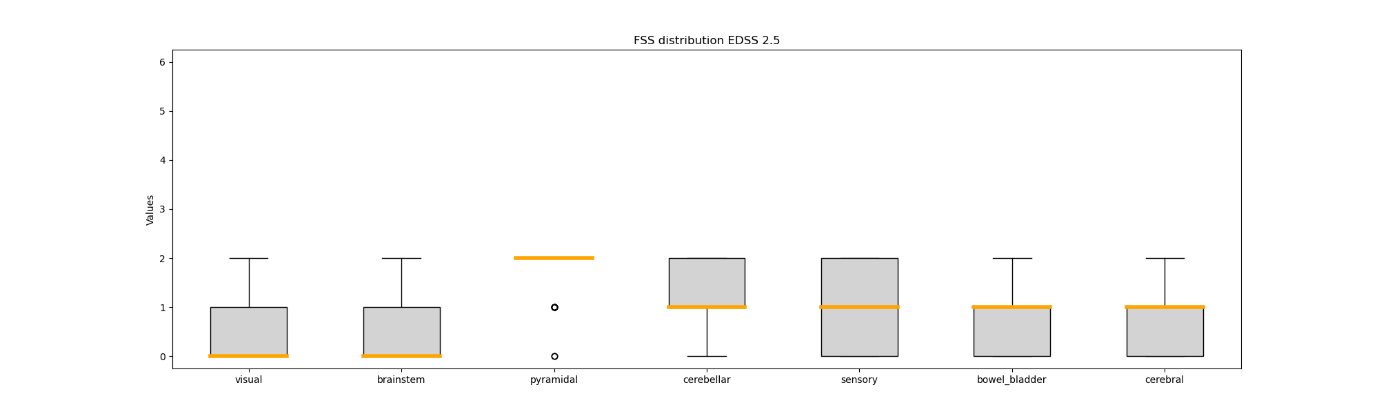

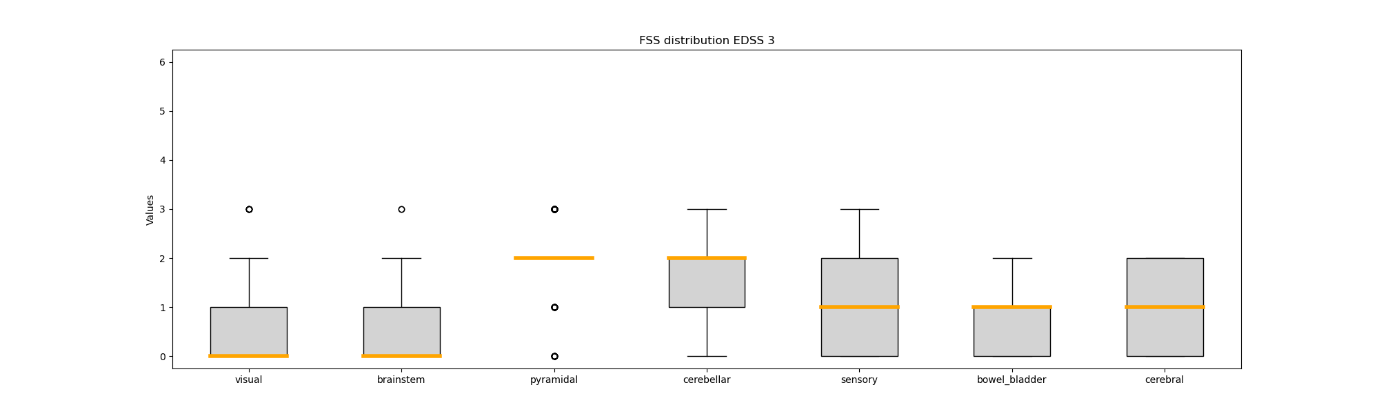

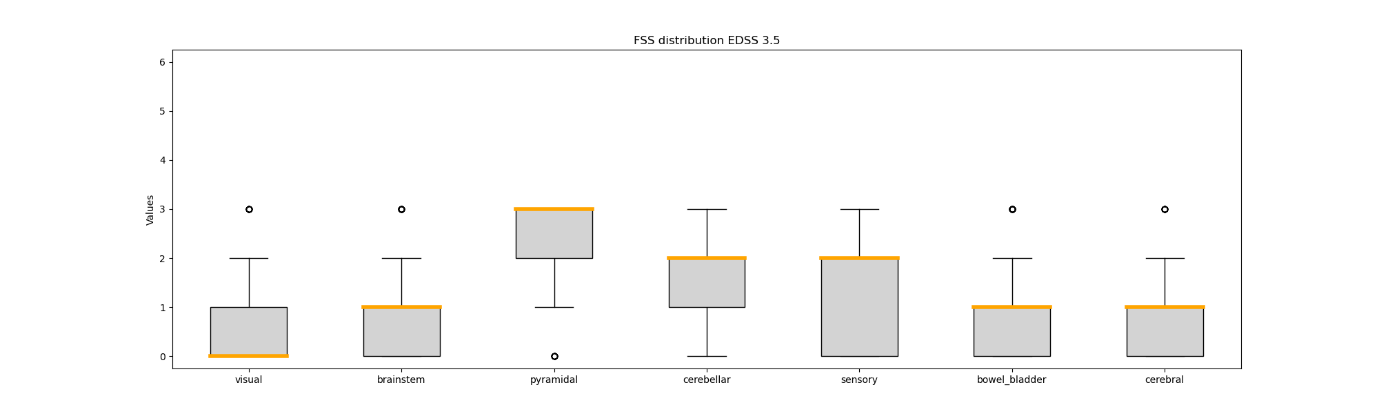

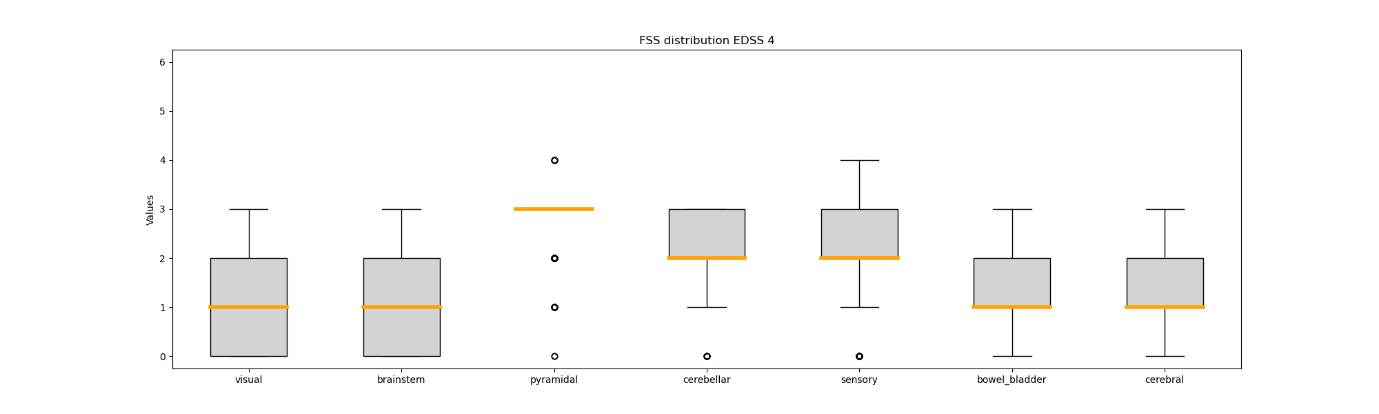

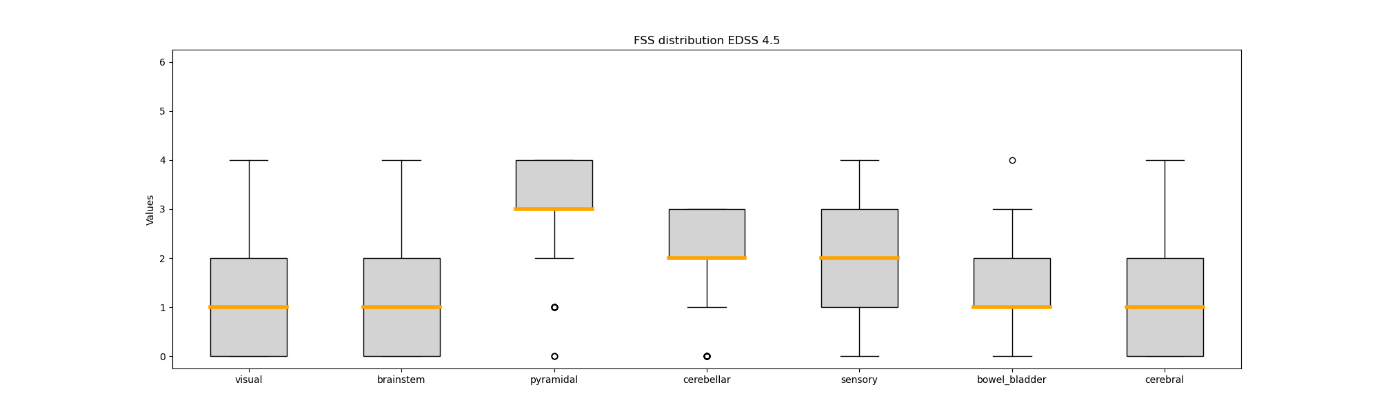

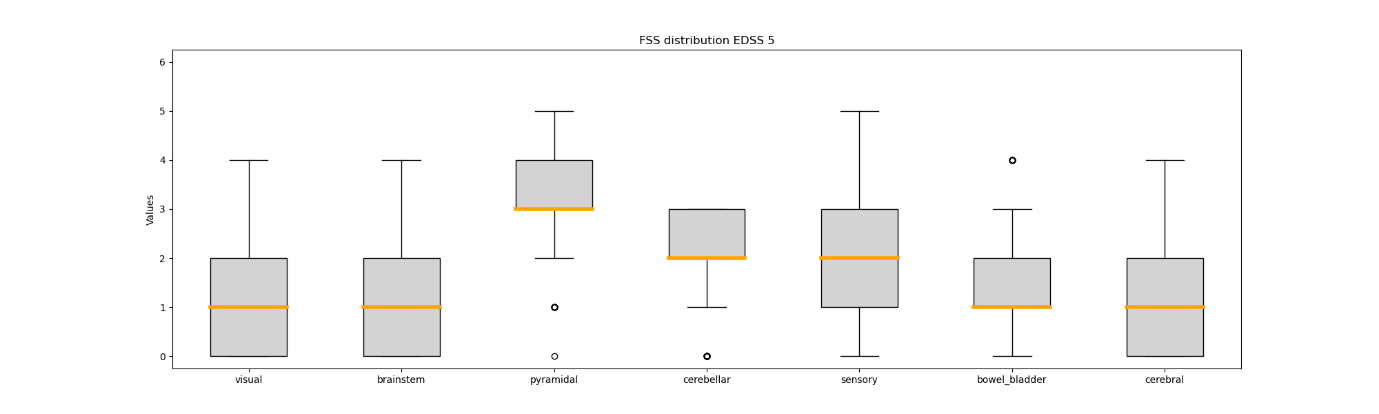

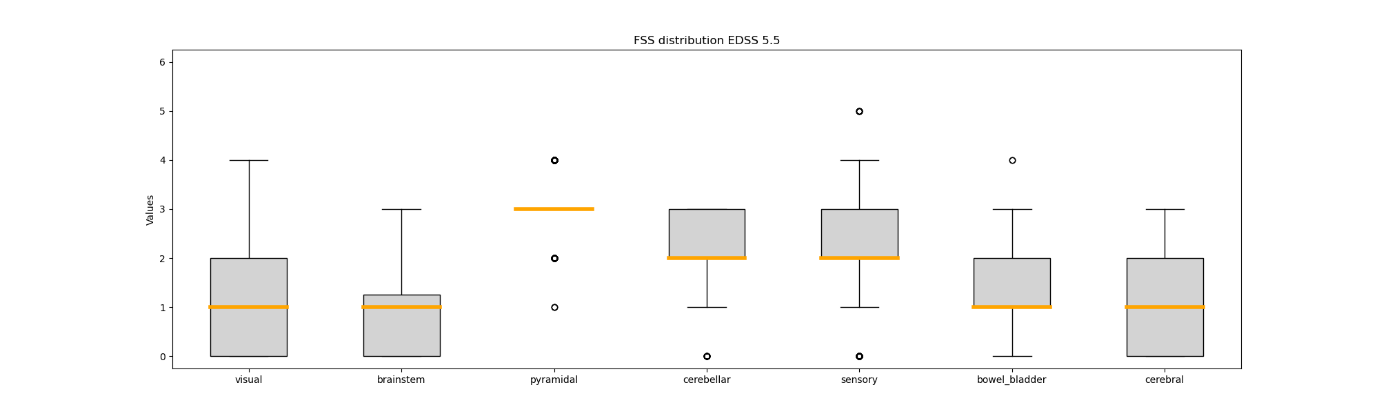

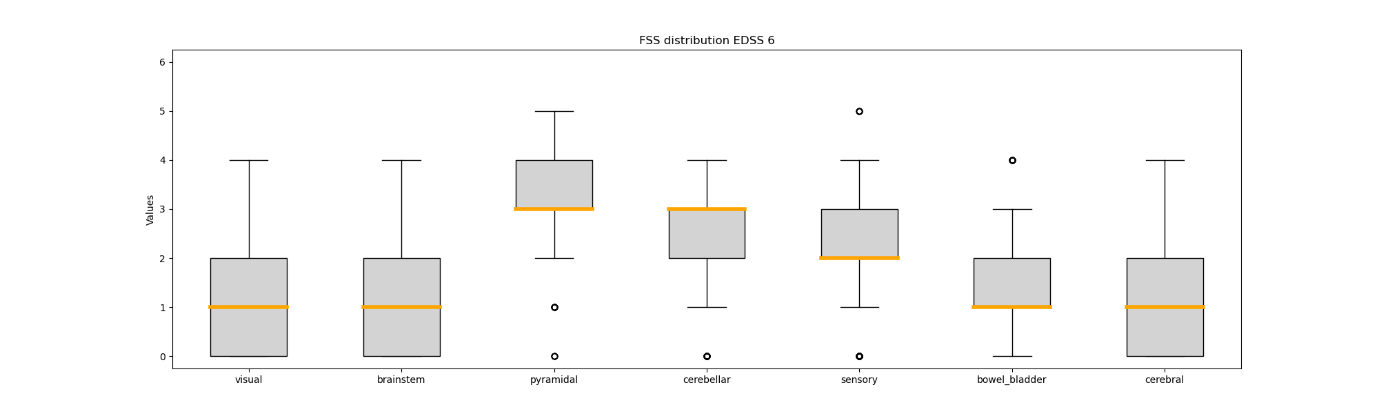


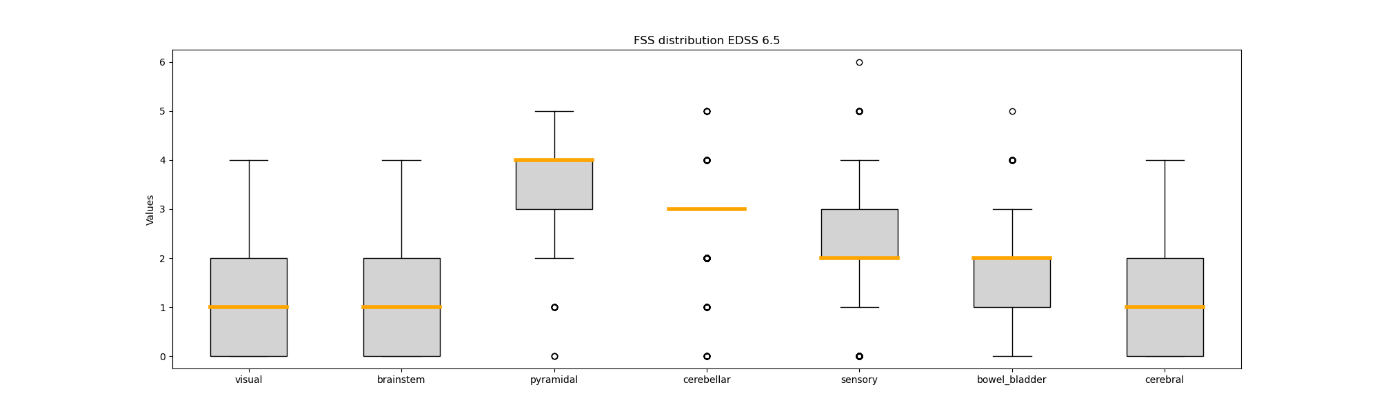


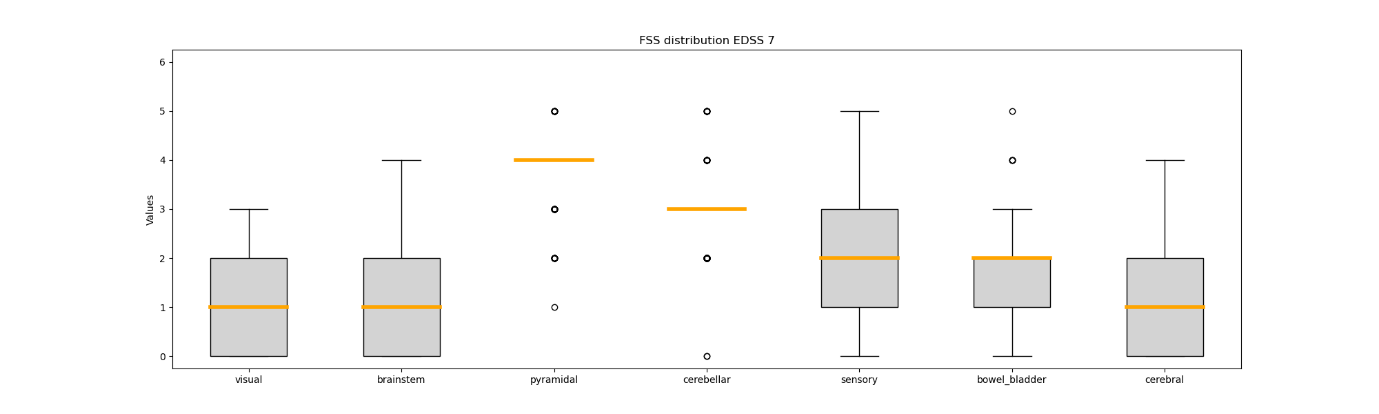


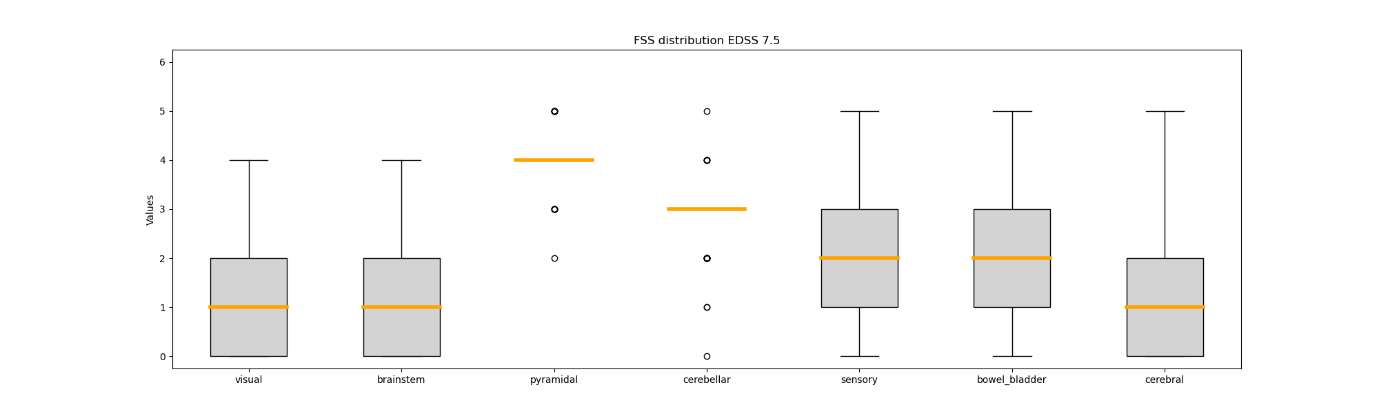


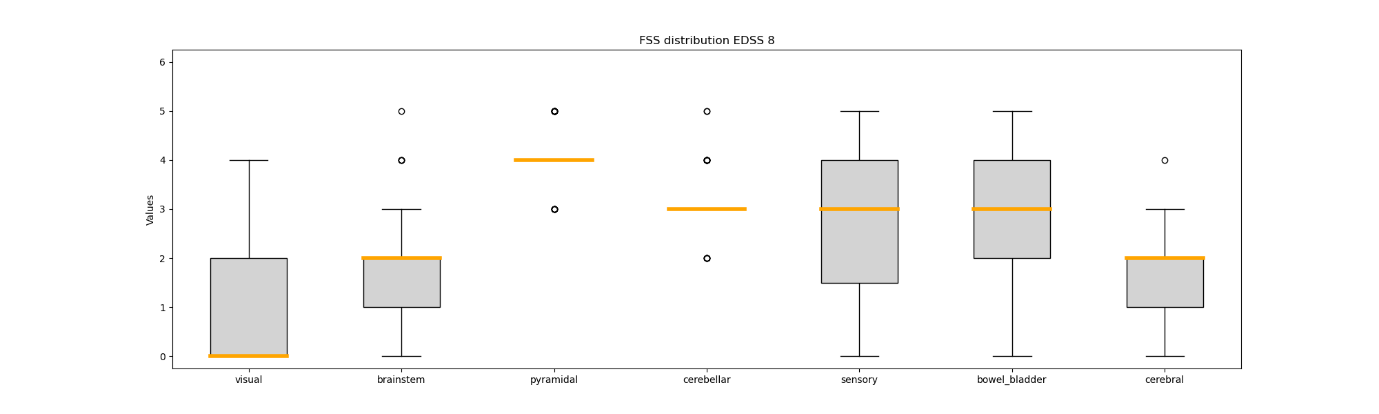


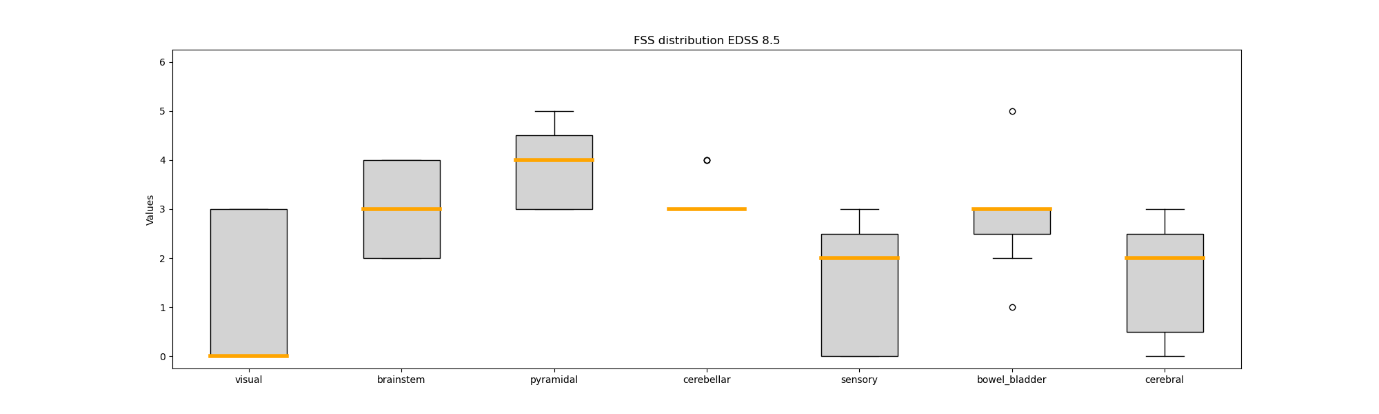


## 5. Subscores Distributions

### 5.1 Distribution of Subscores for each FSS

The distribution of each subscore is presented for every FSS value. Subscores highlighted in dark grey are those included in the cluster analysis.


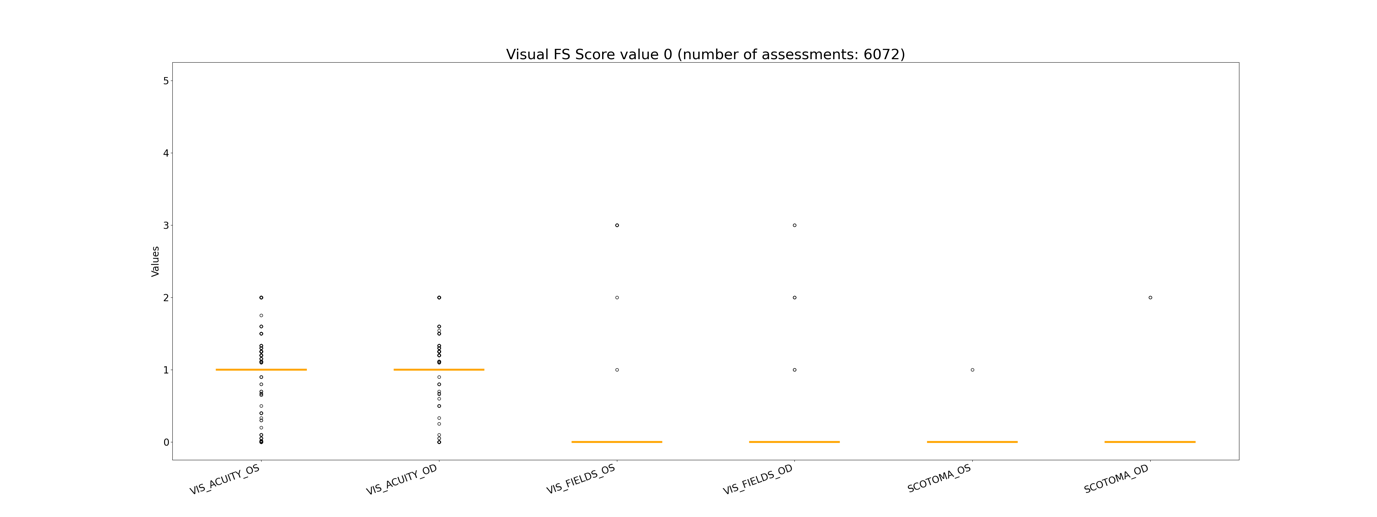

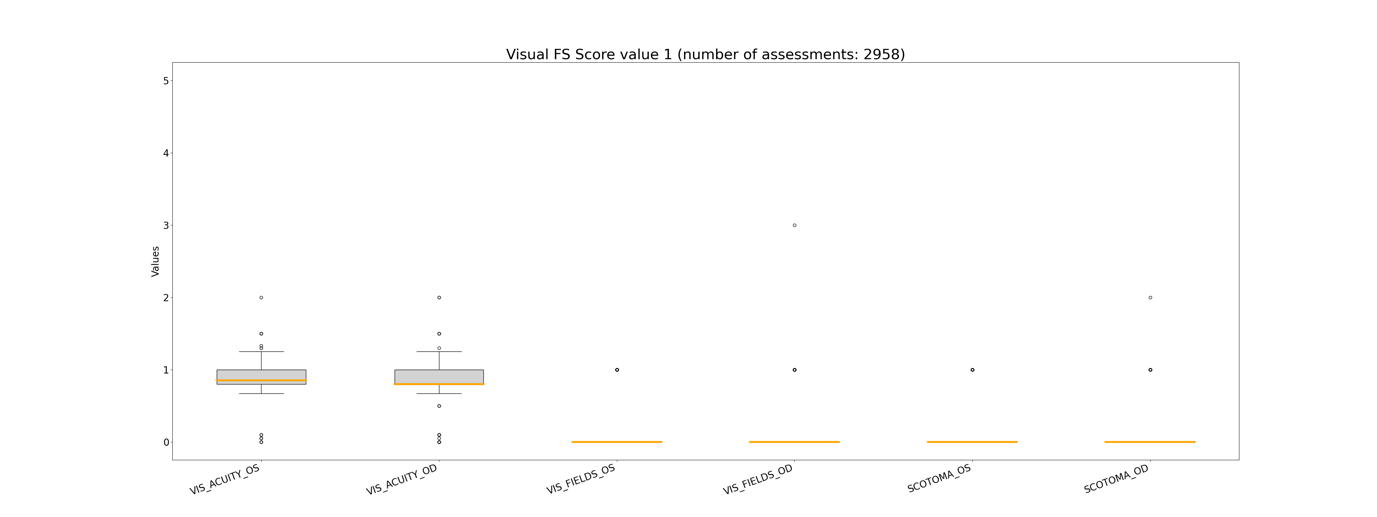

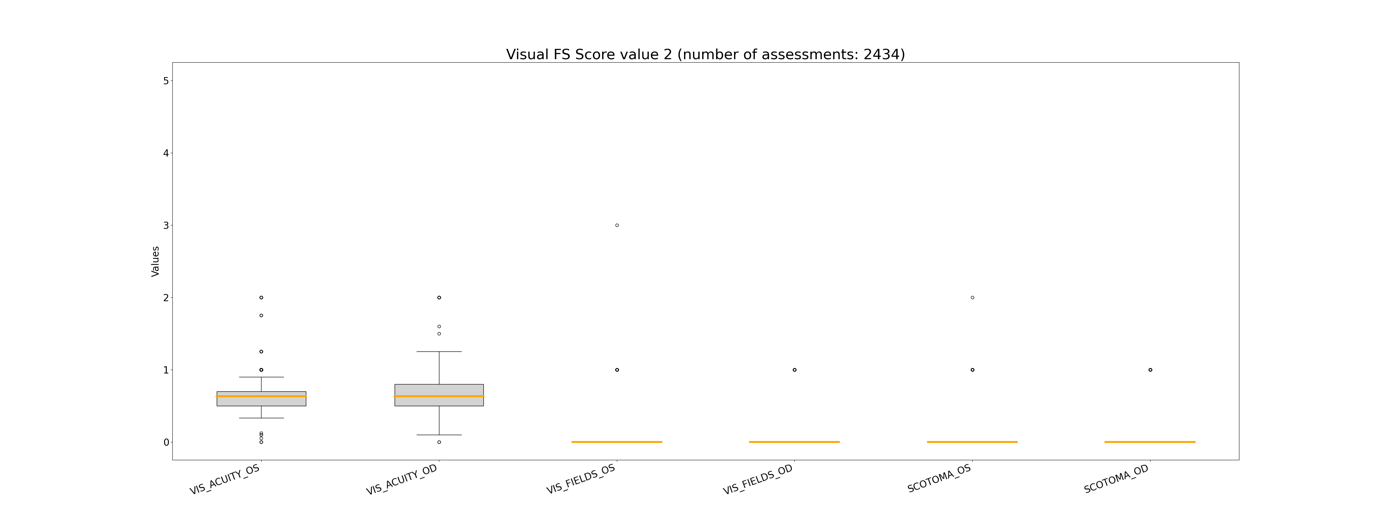

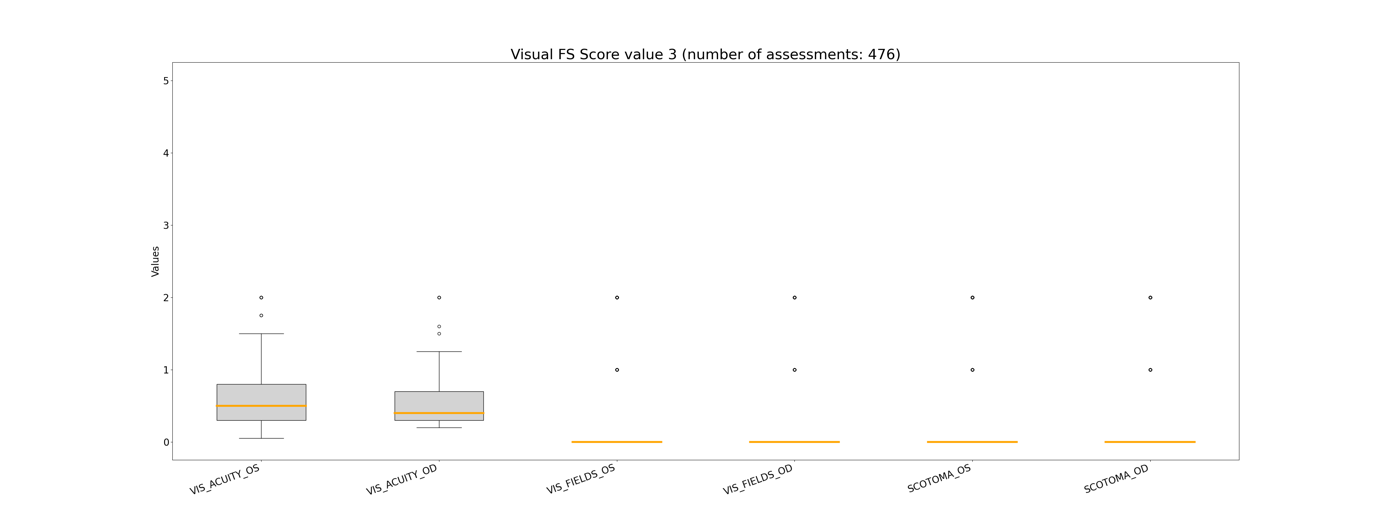

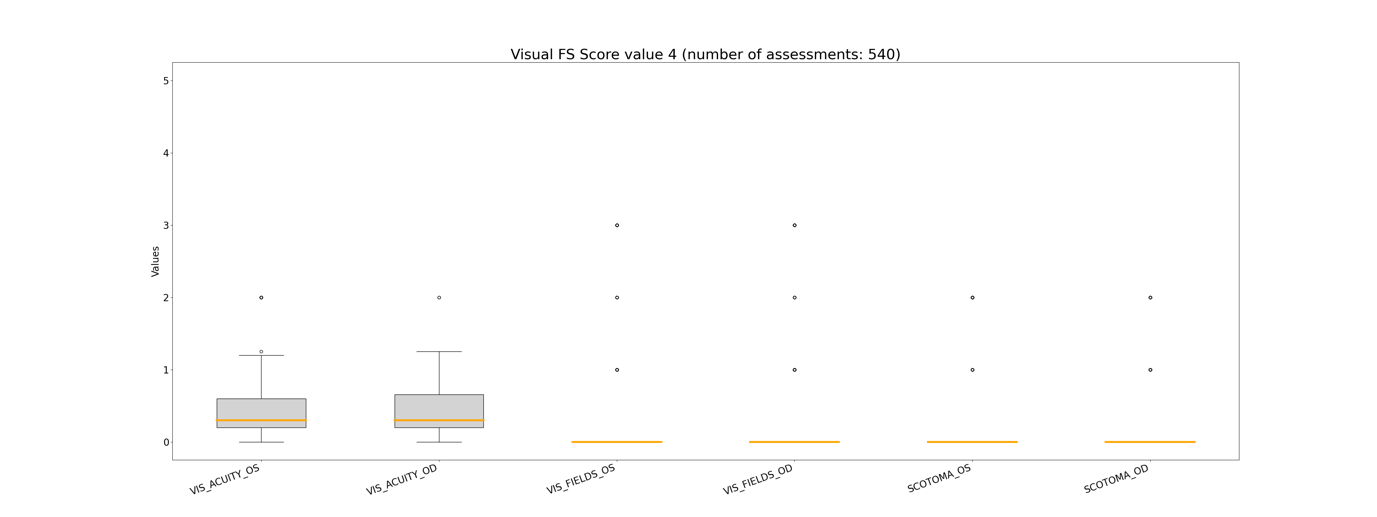

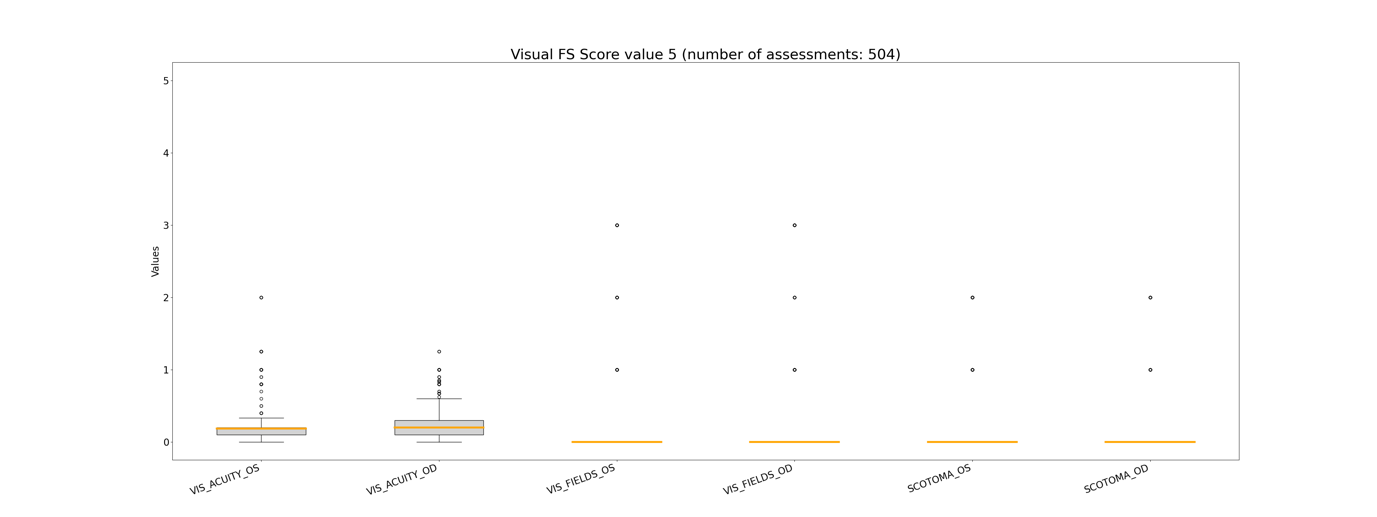

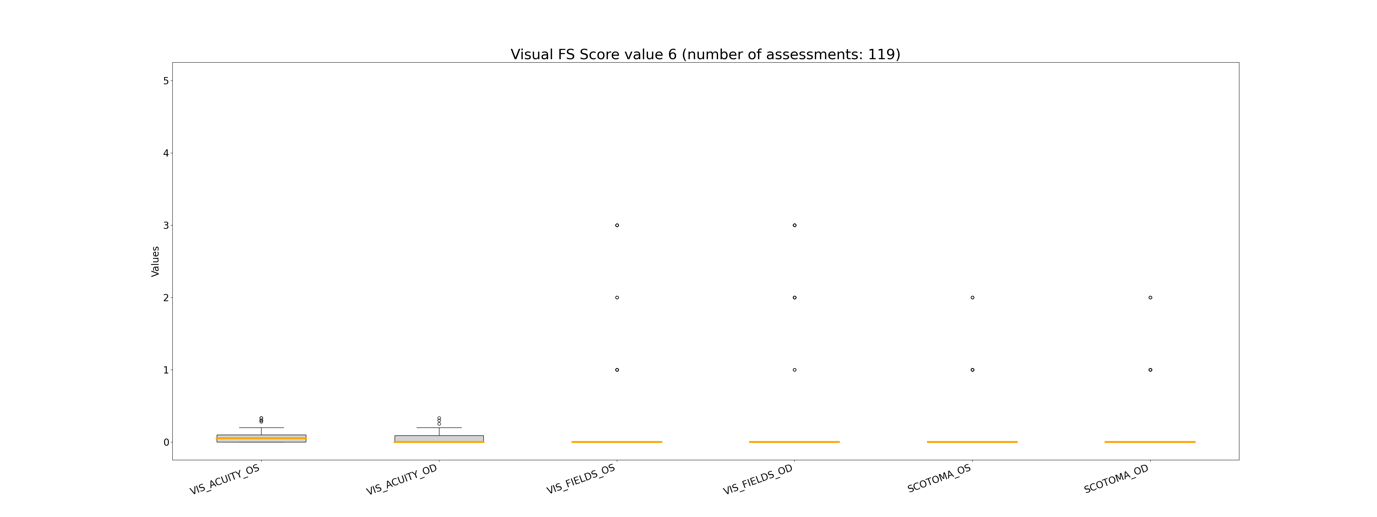


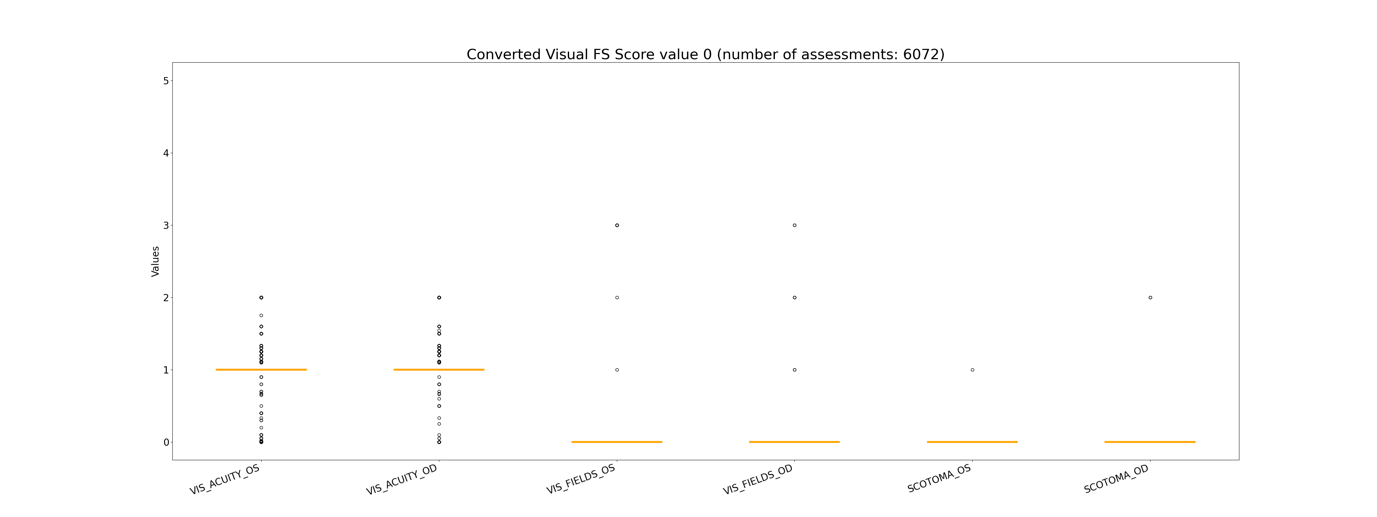

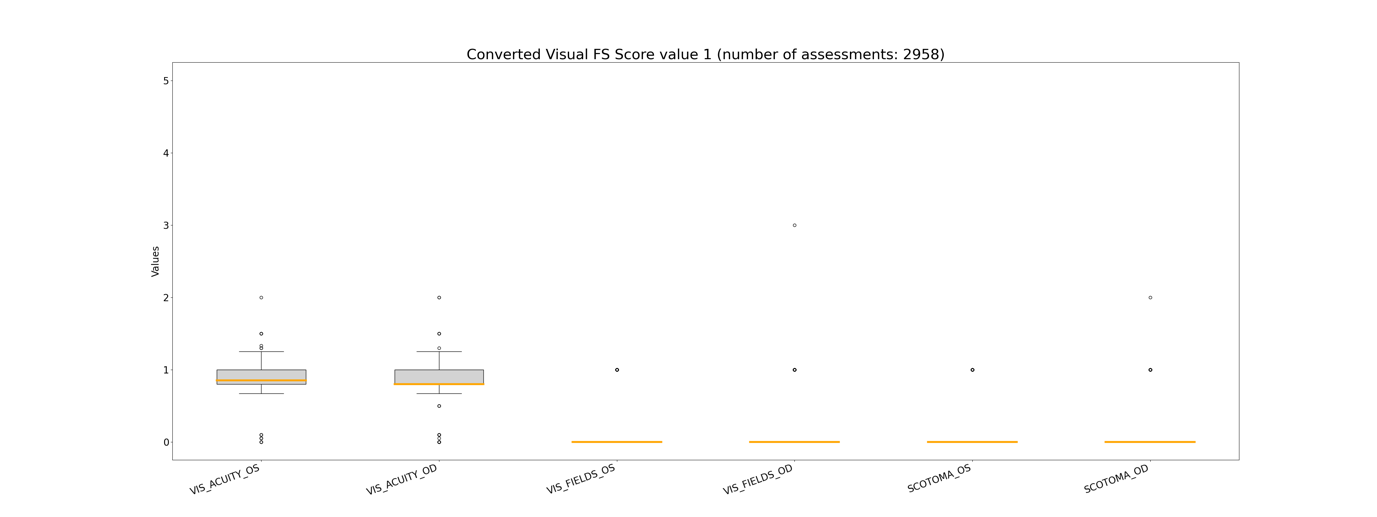

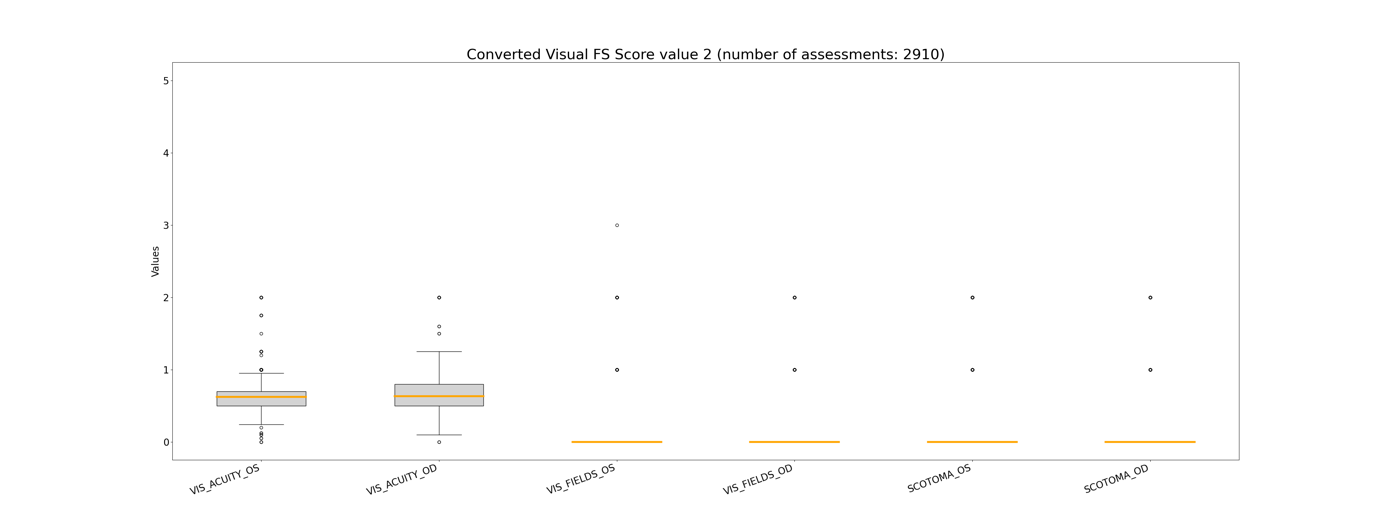

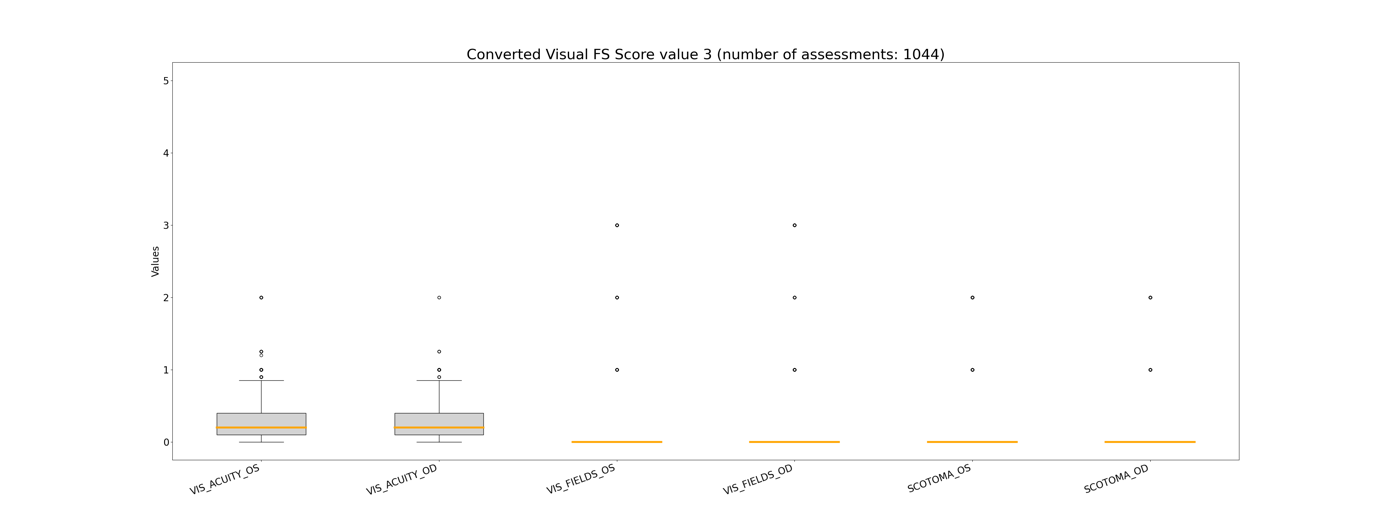

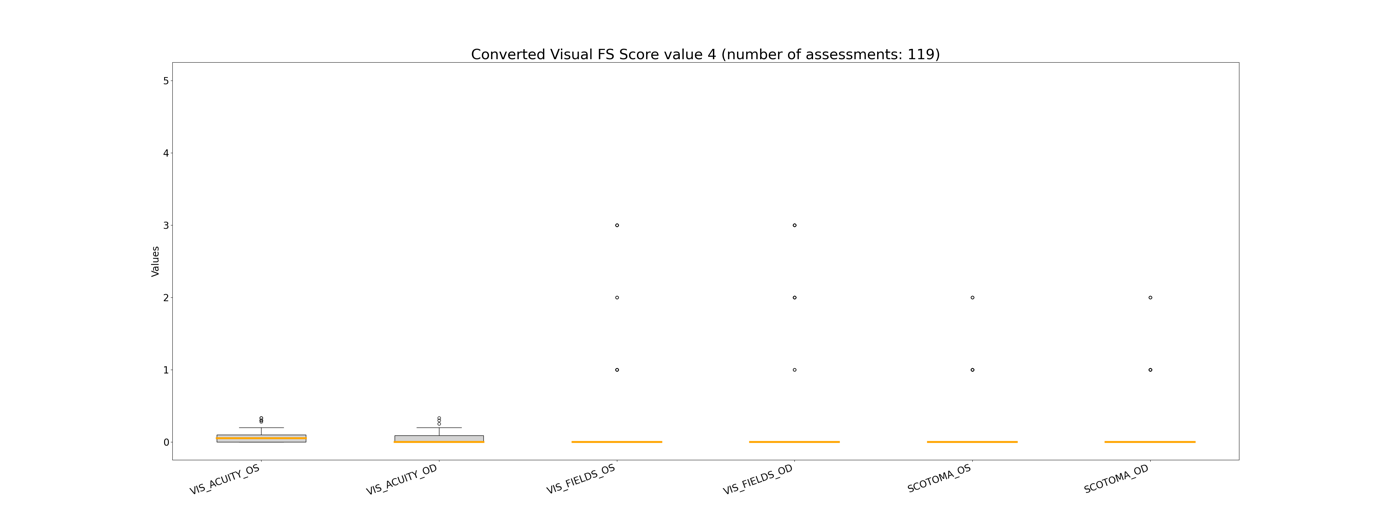


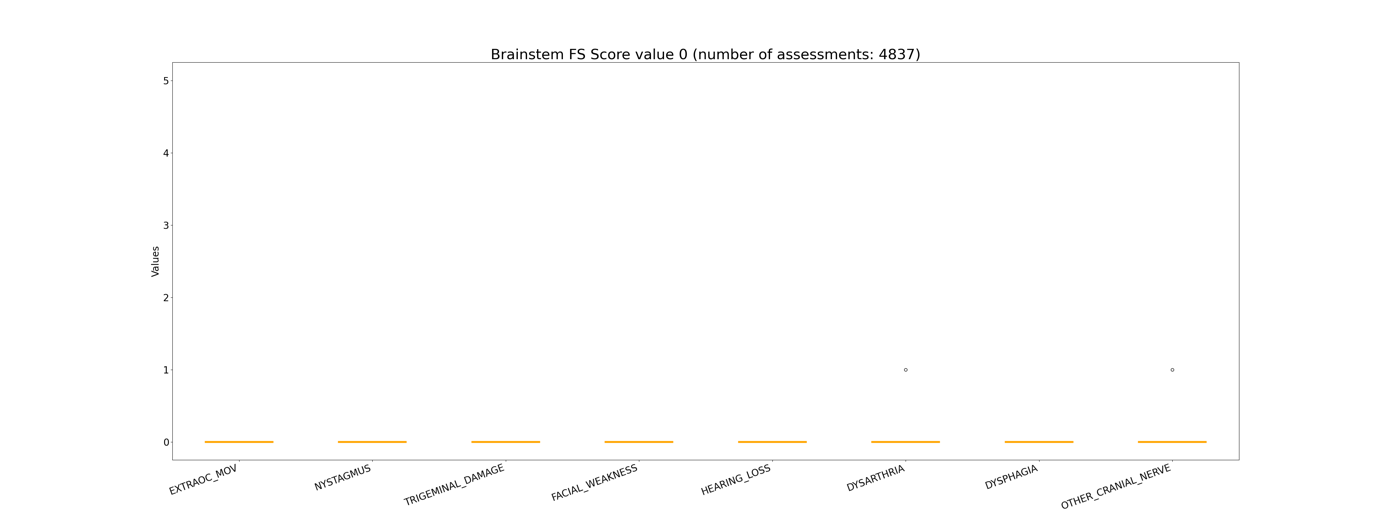


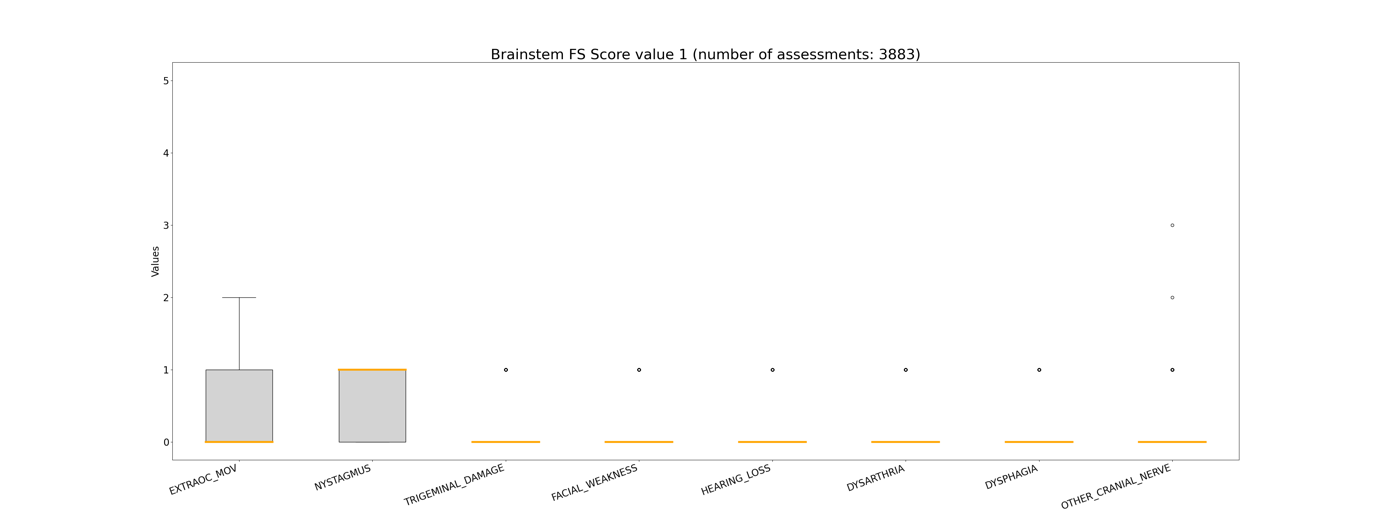

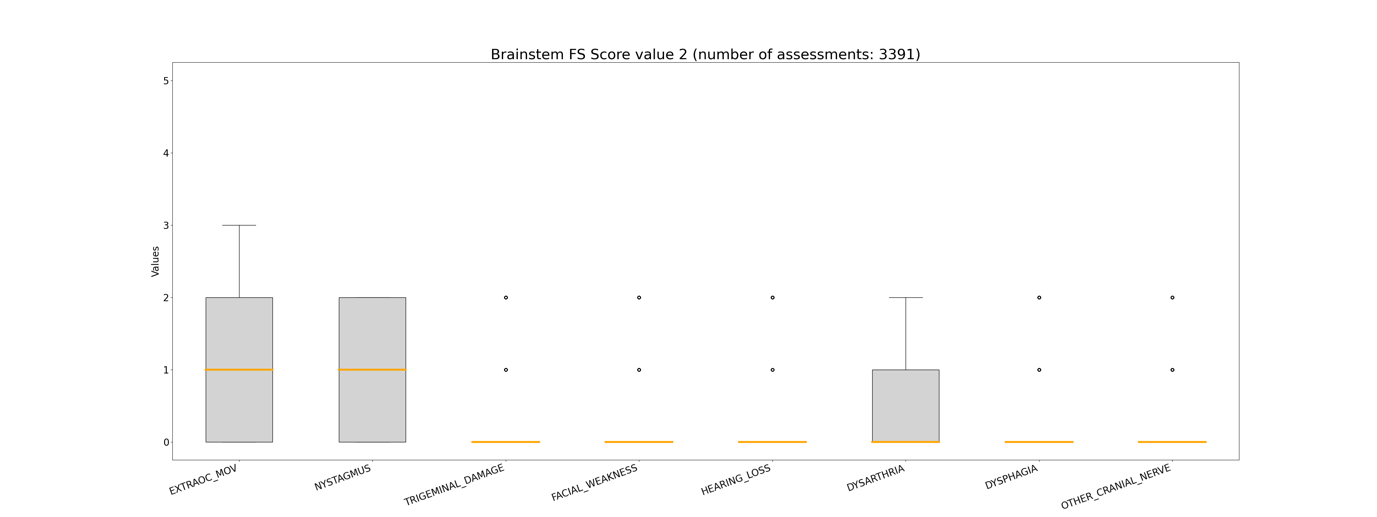

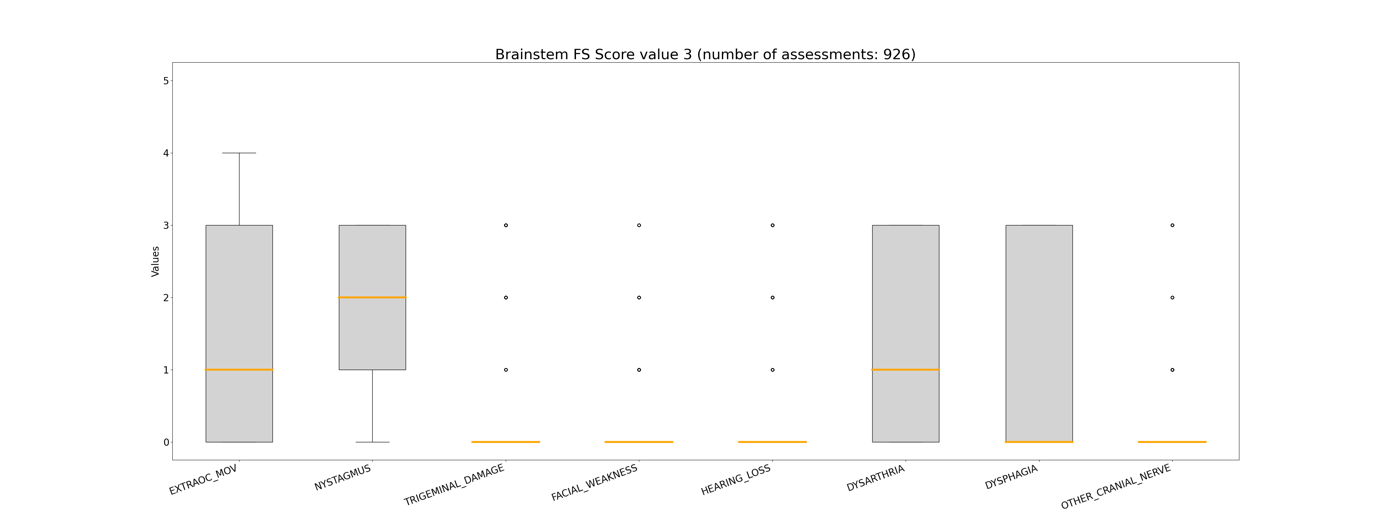

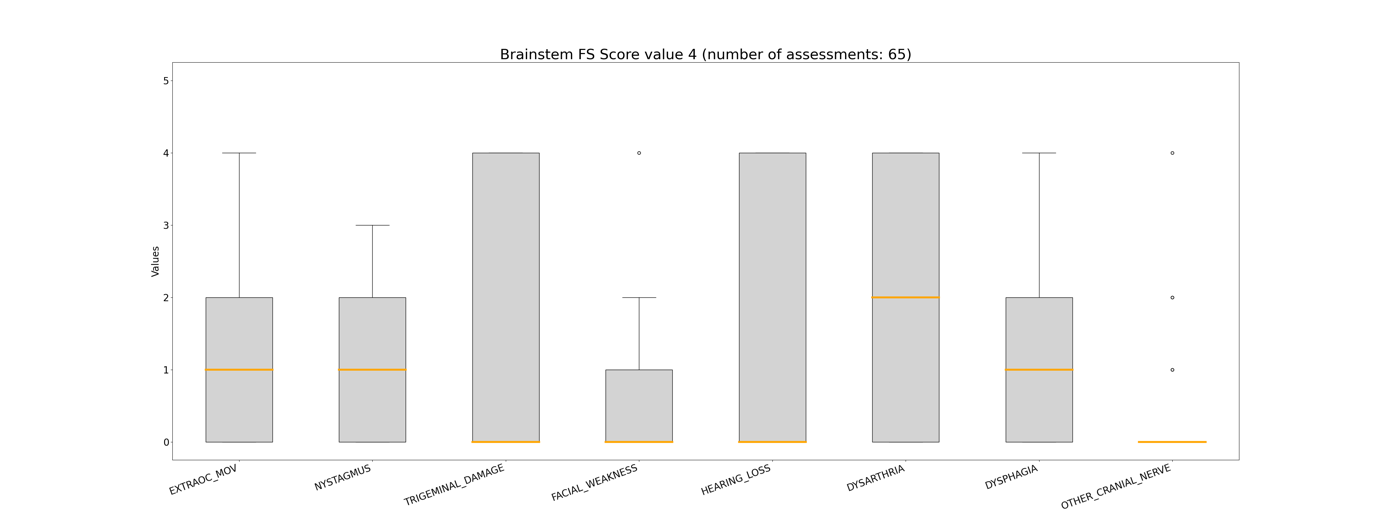

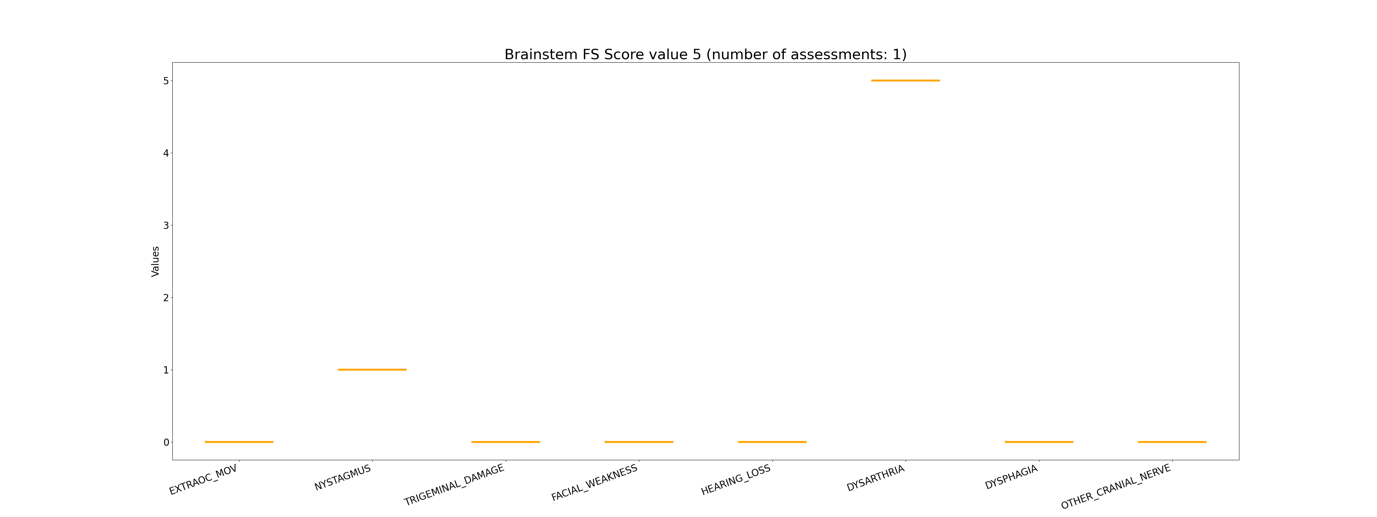


##
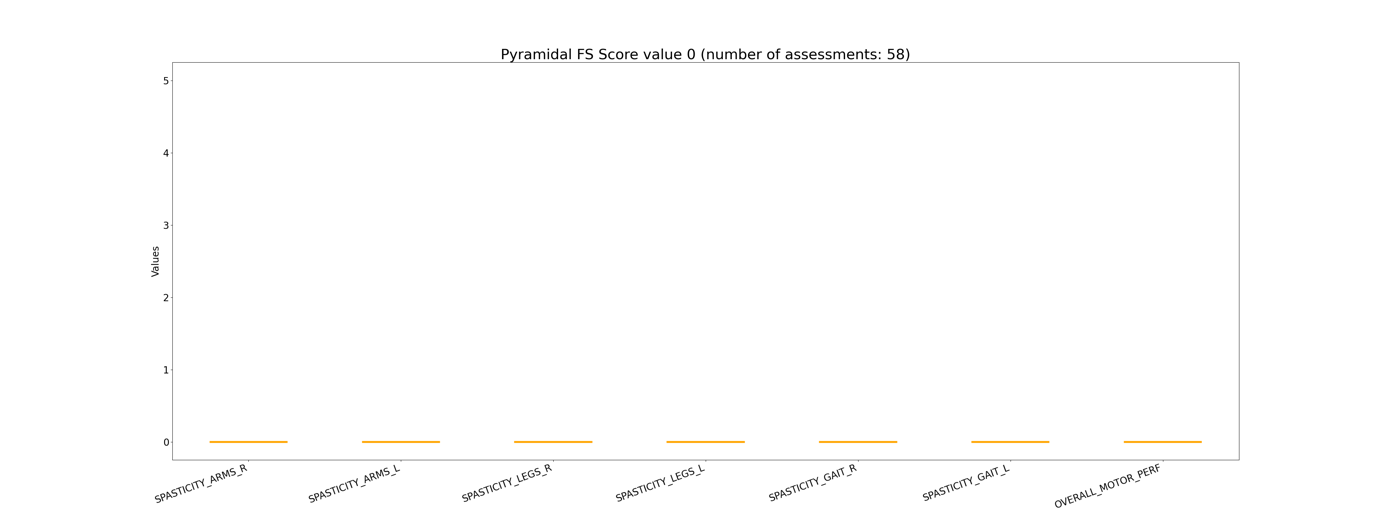

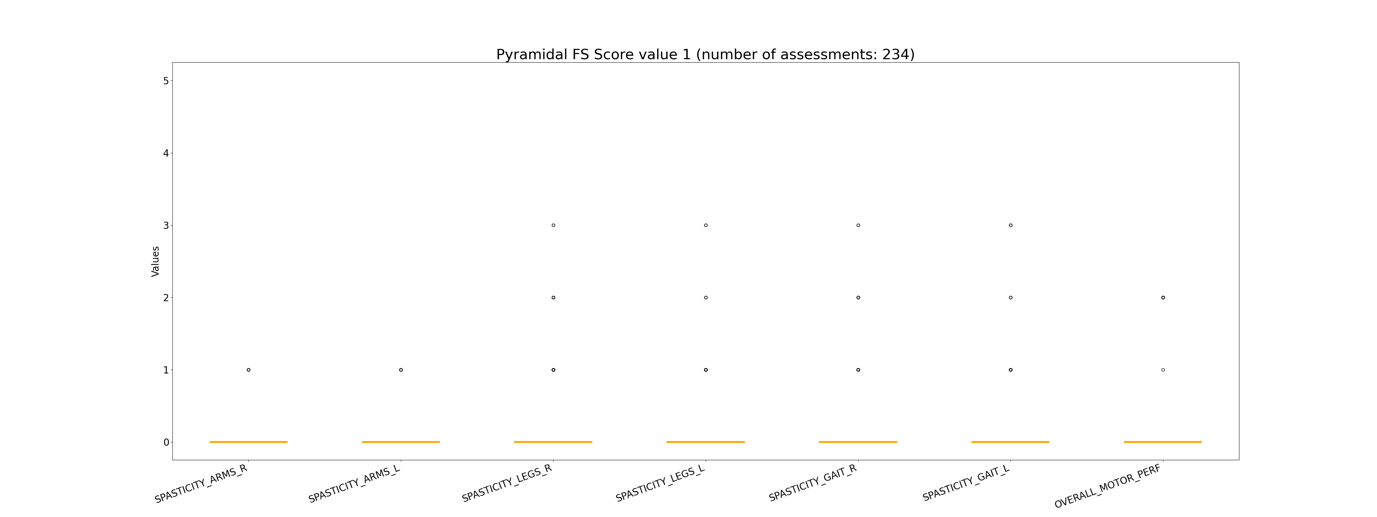


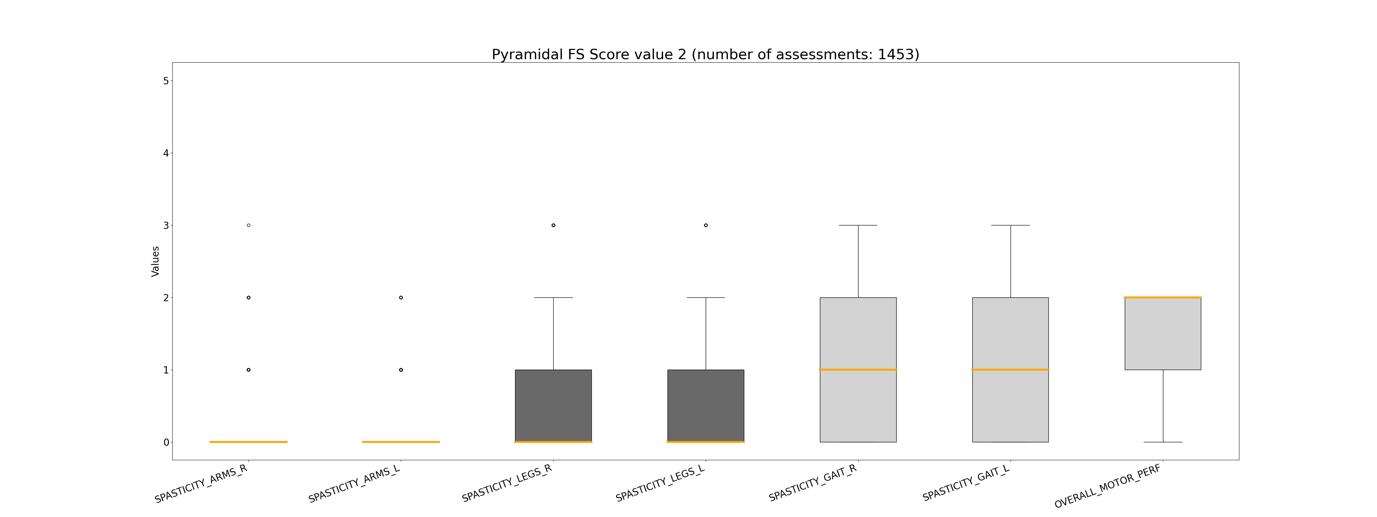


##
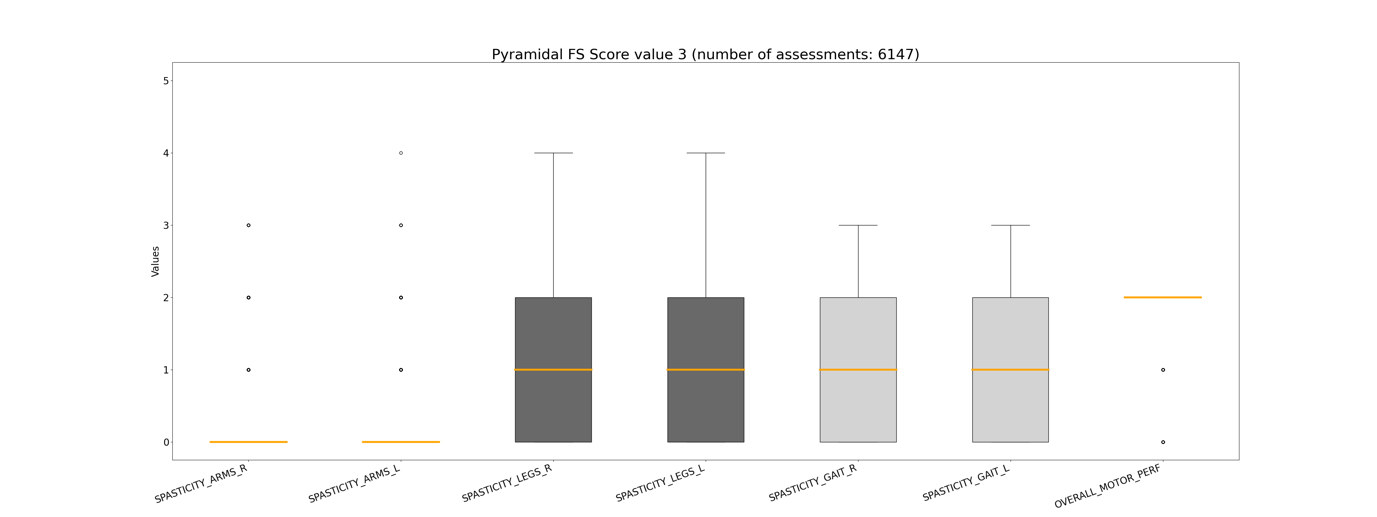

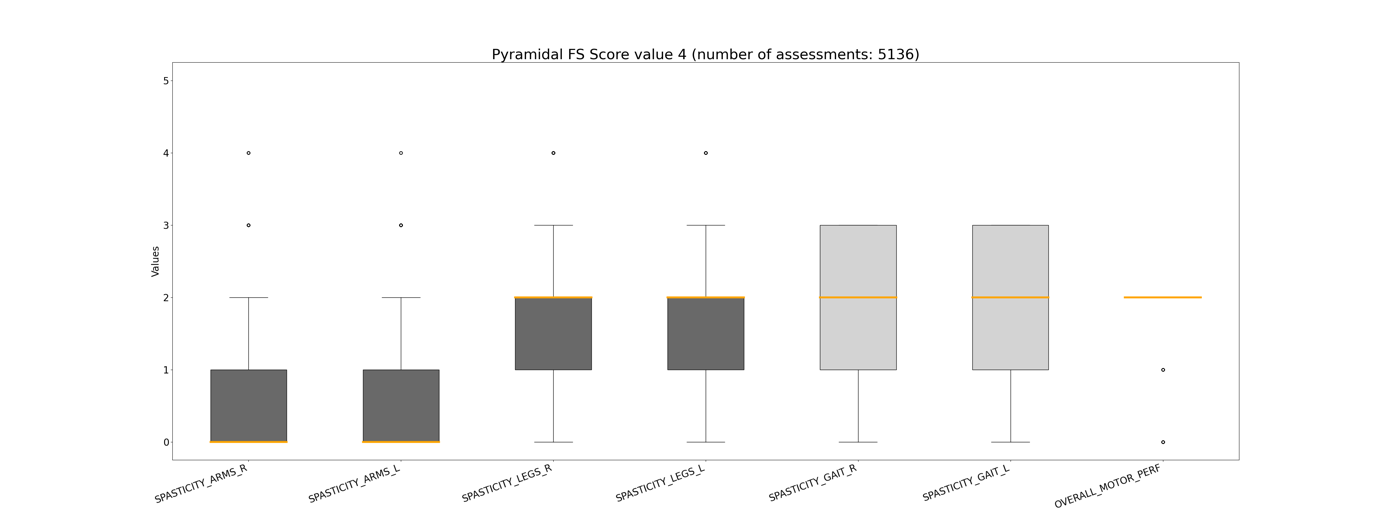

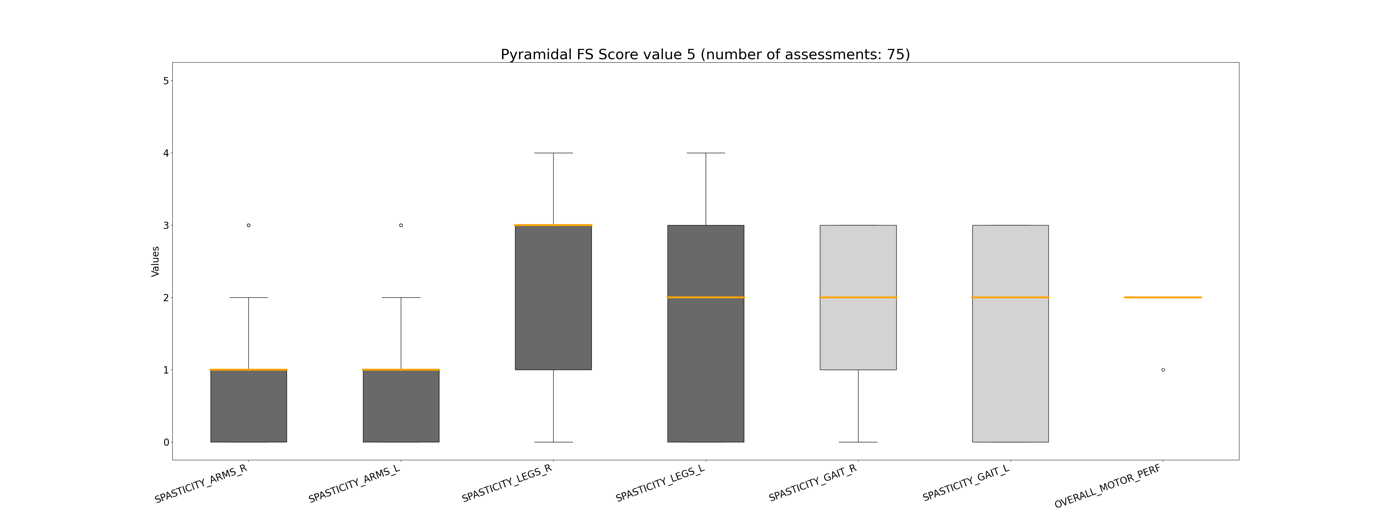


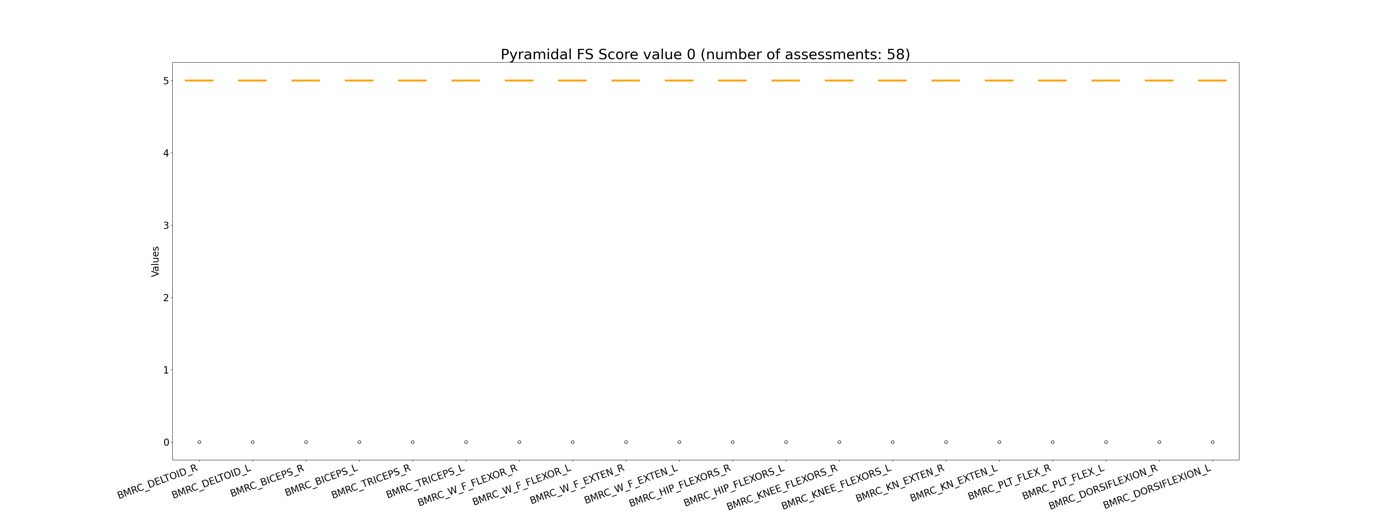


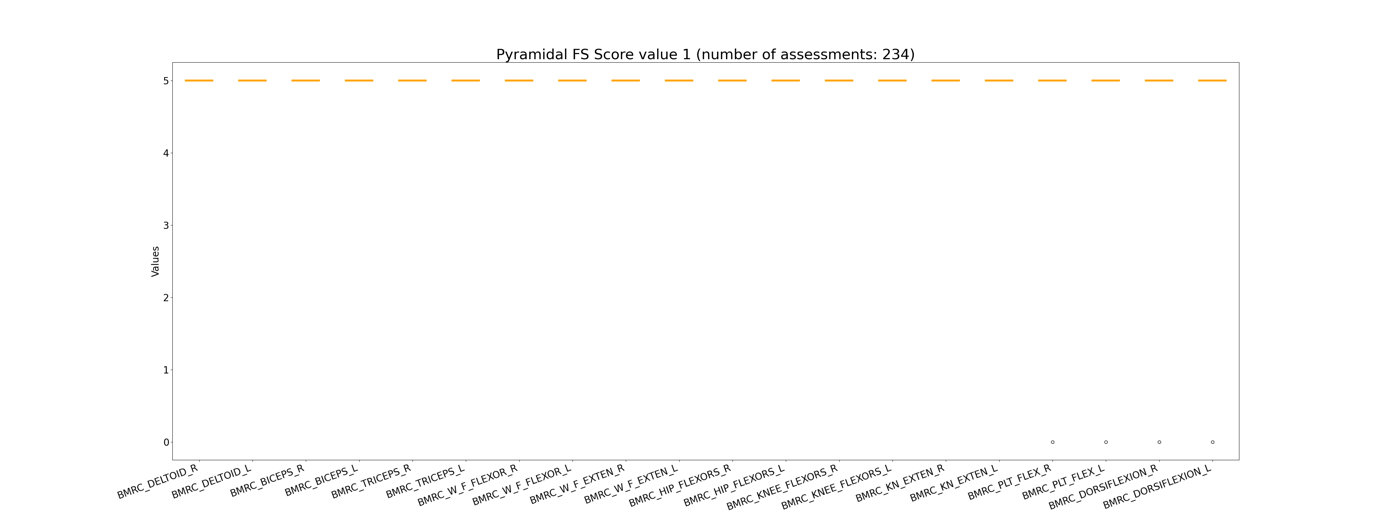

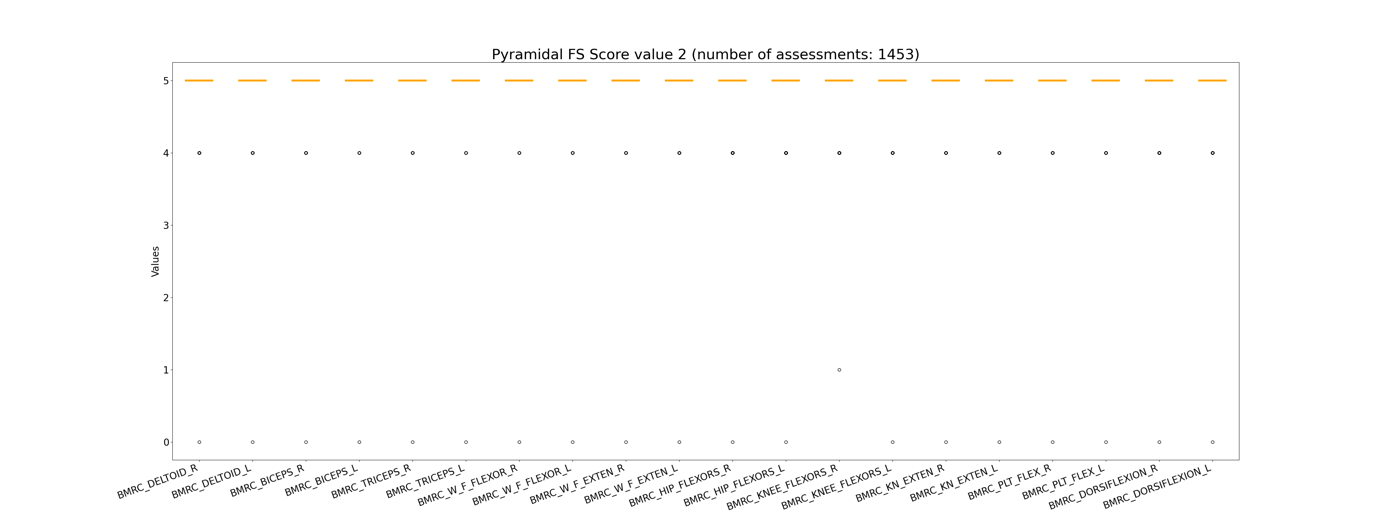

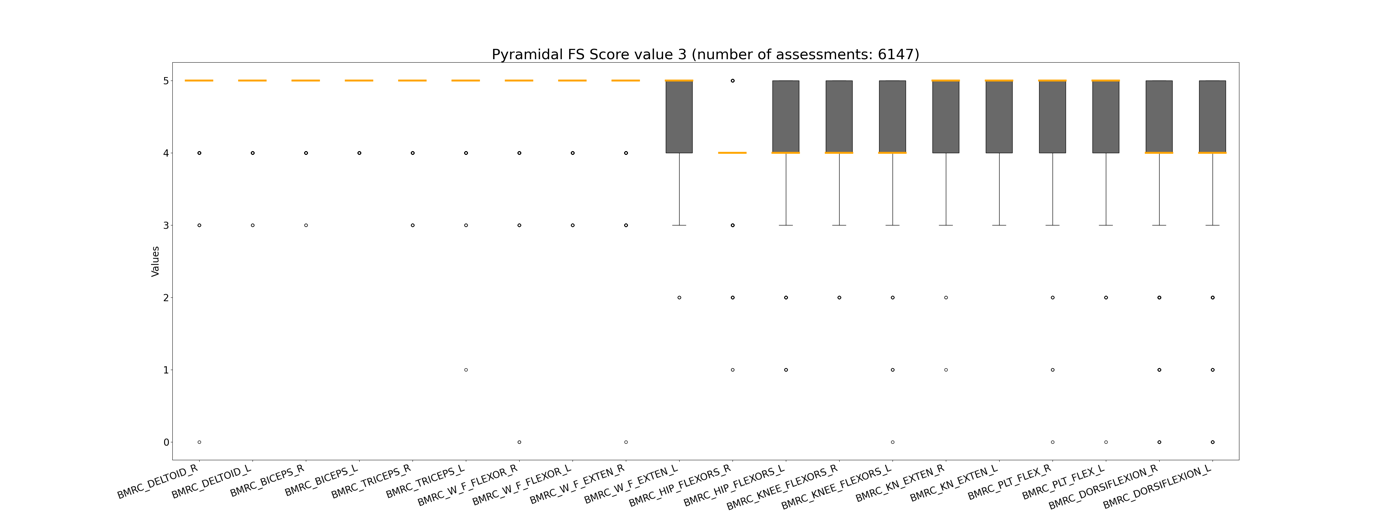

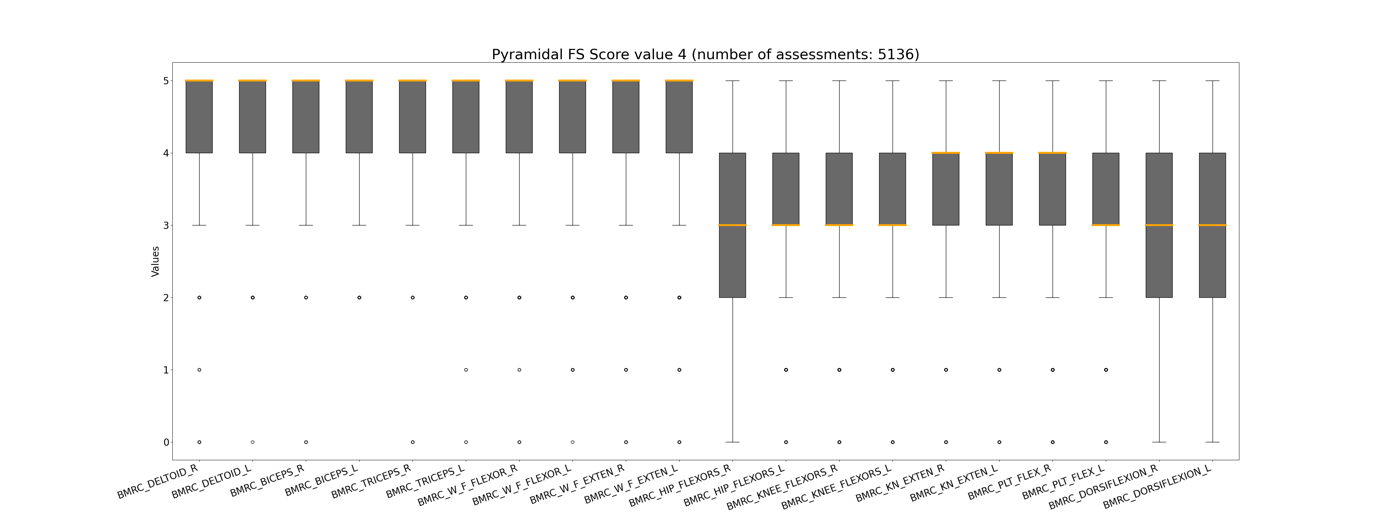

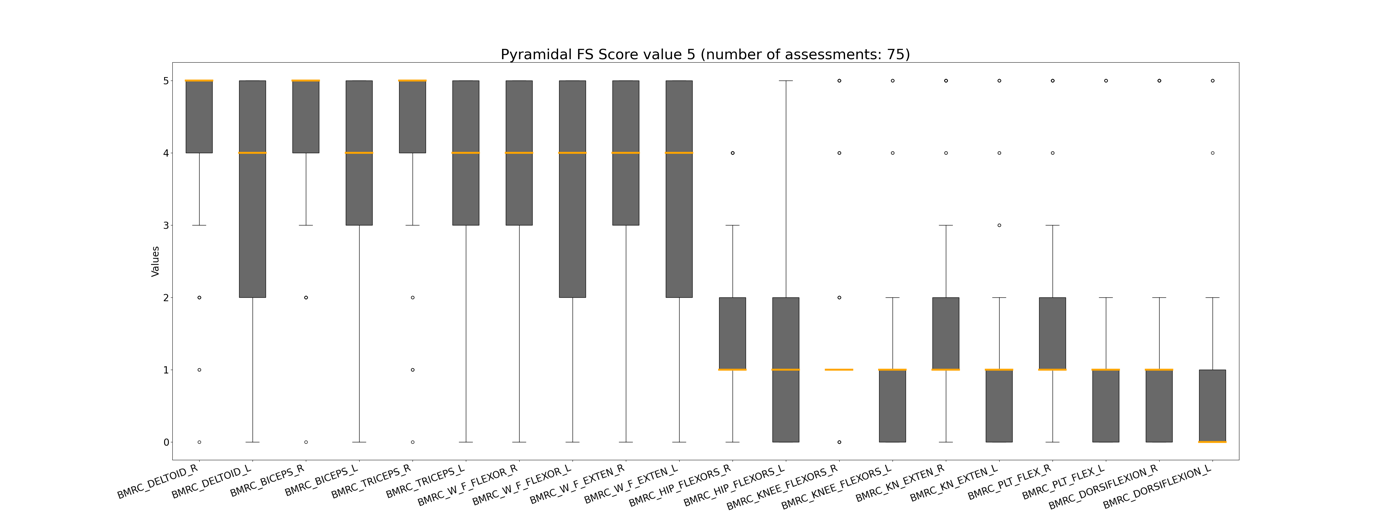


##
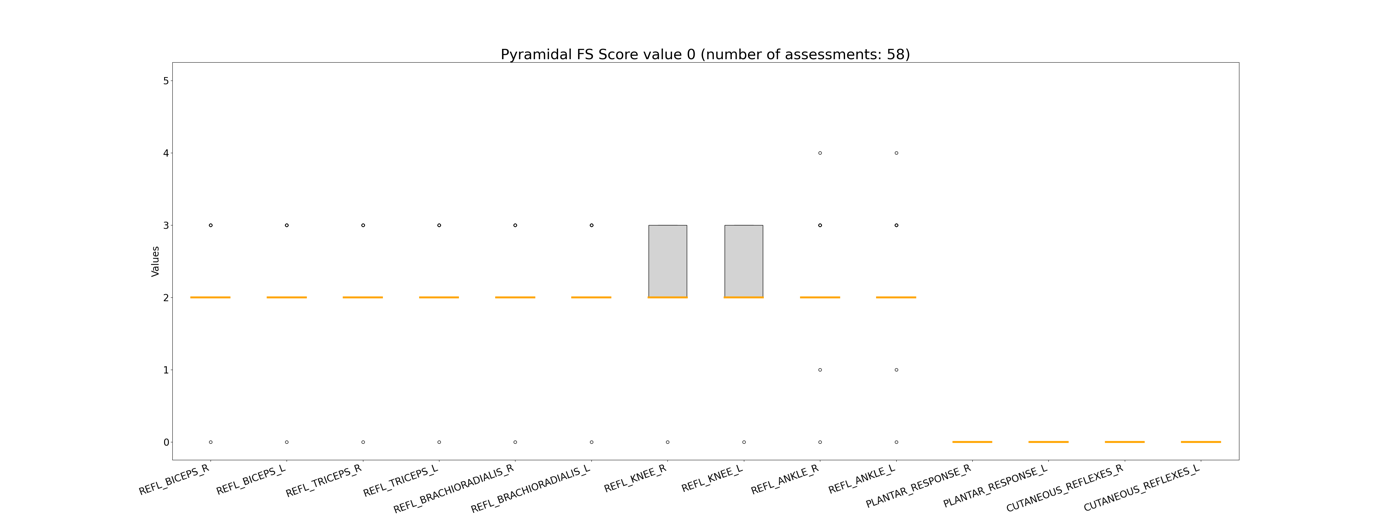

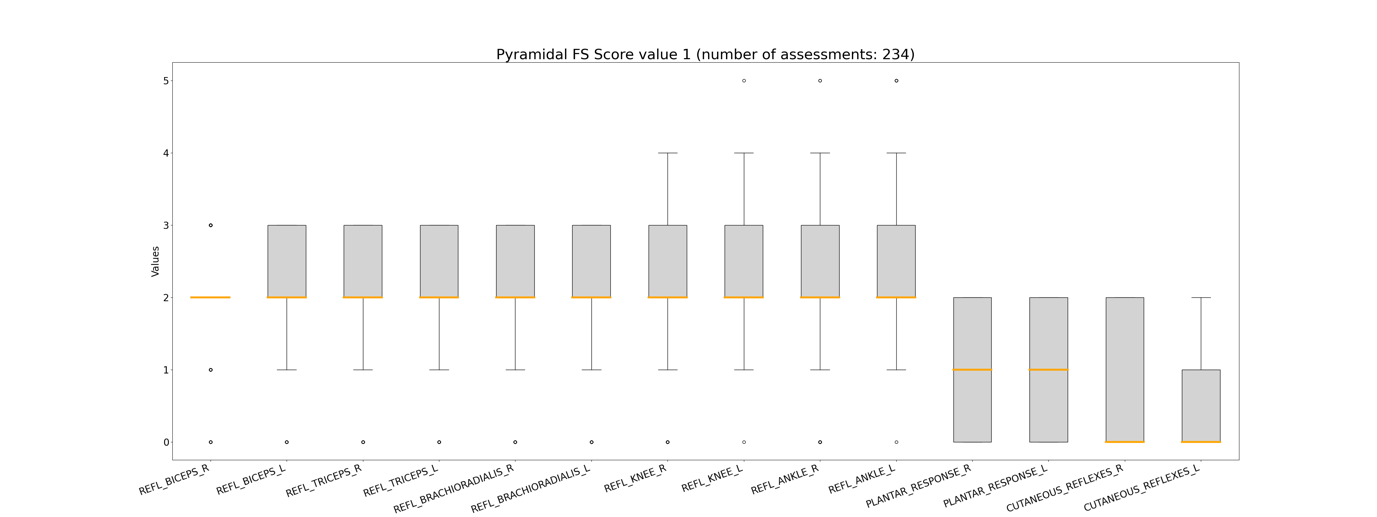

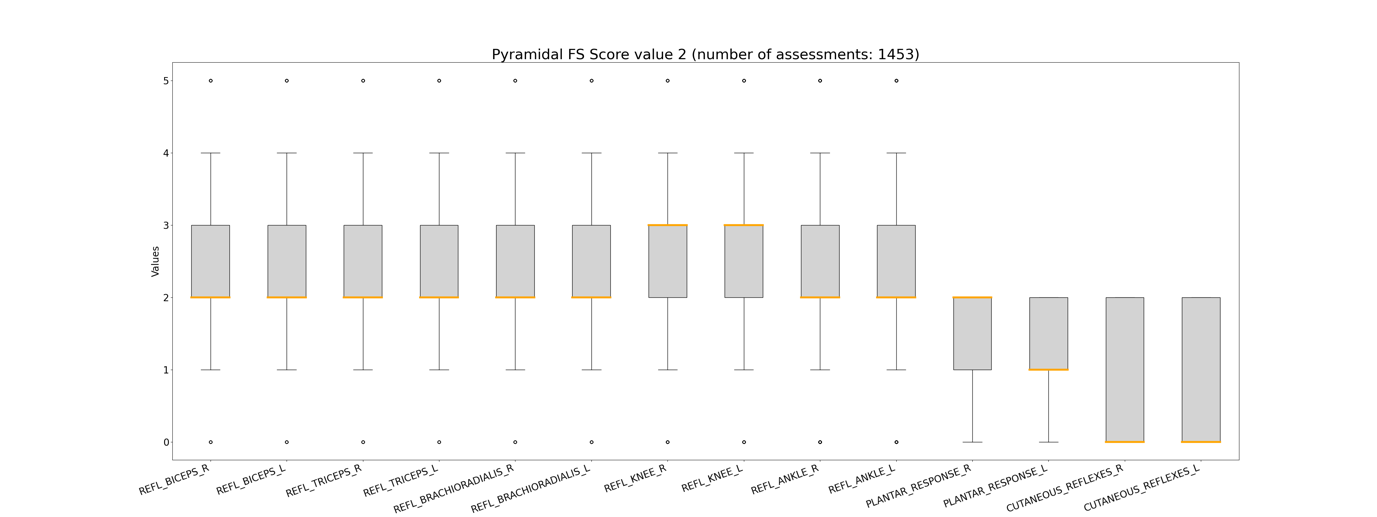

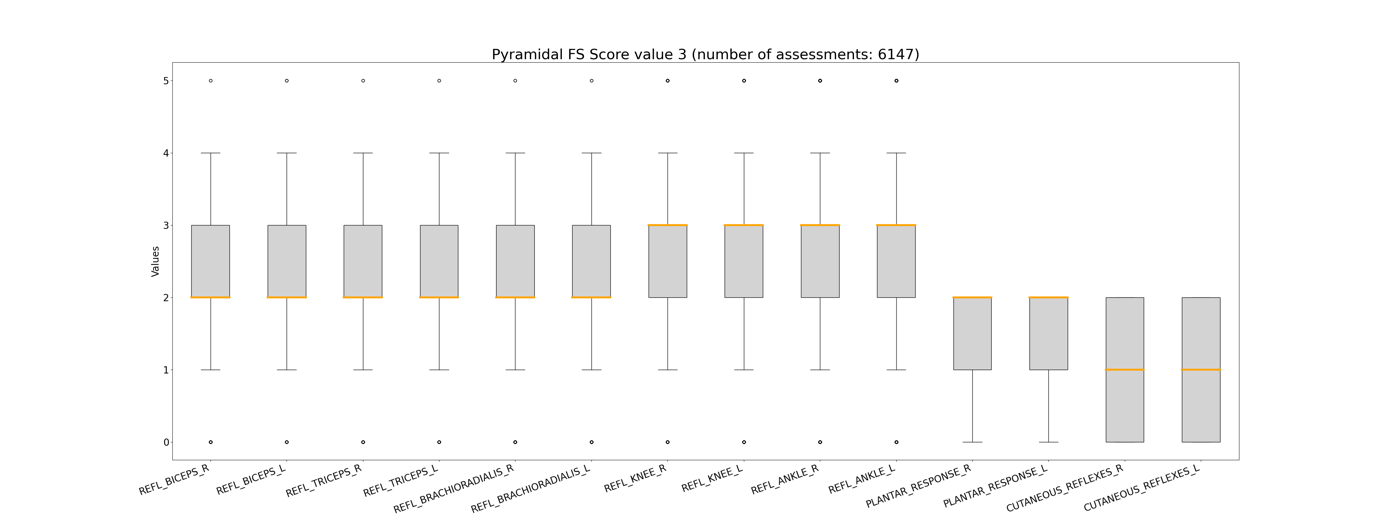

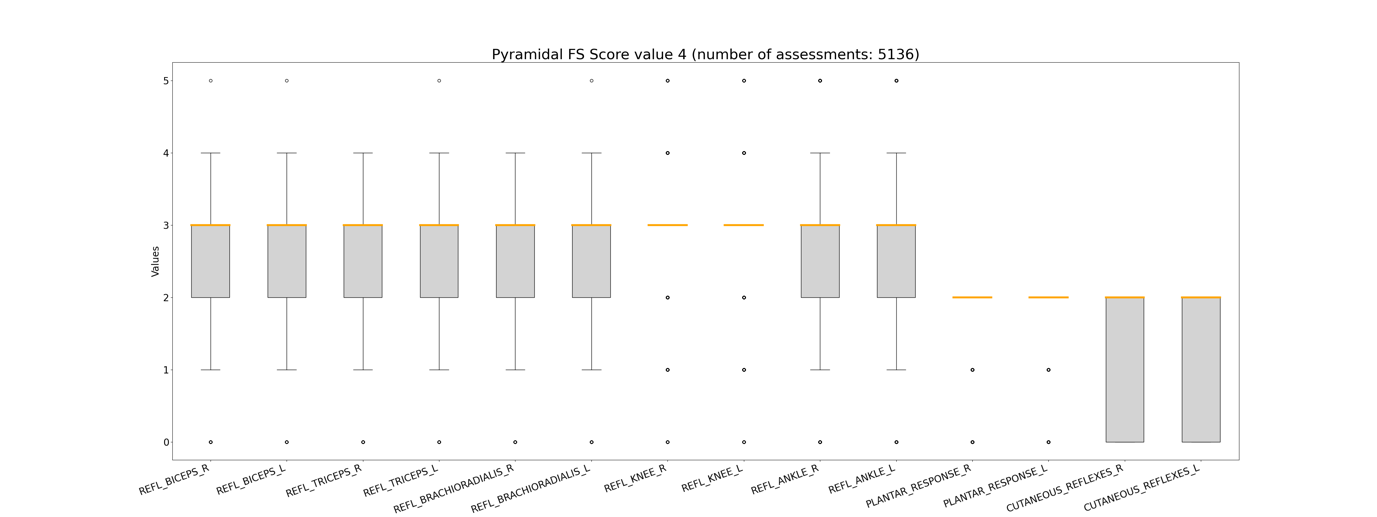

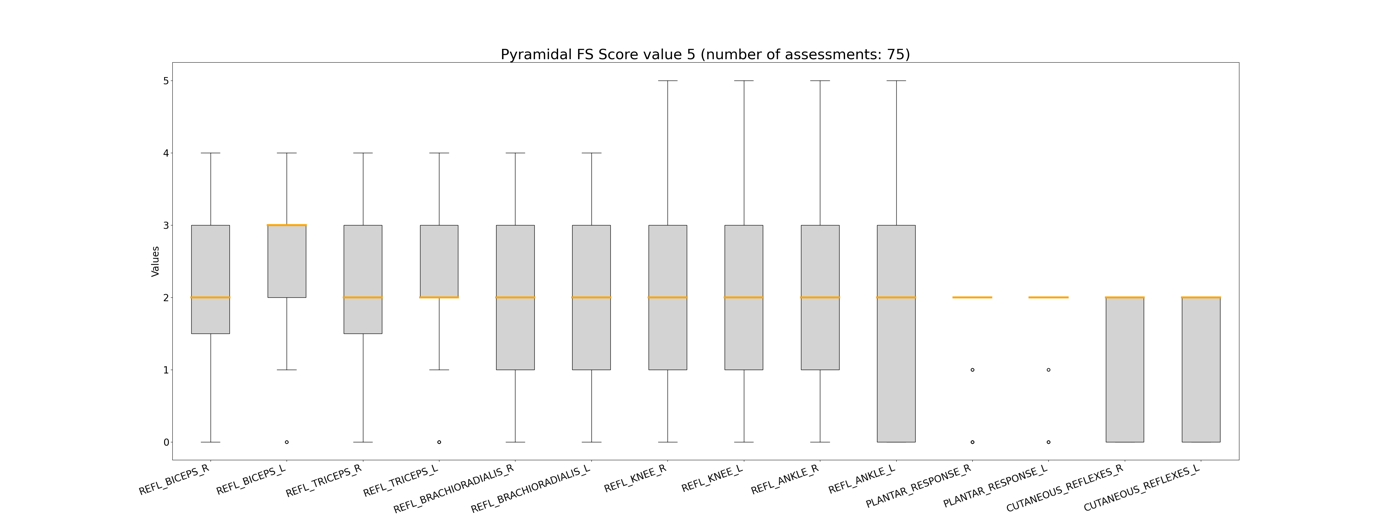


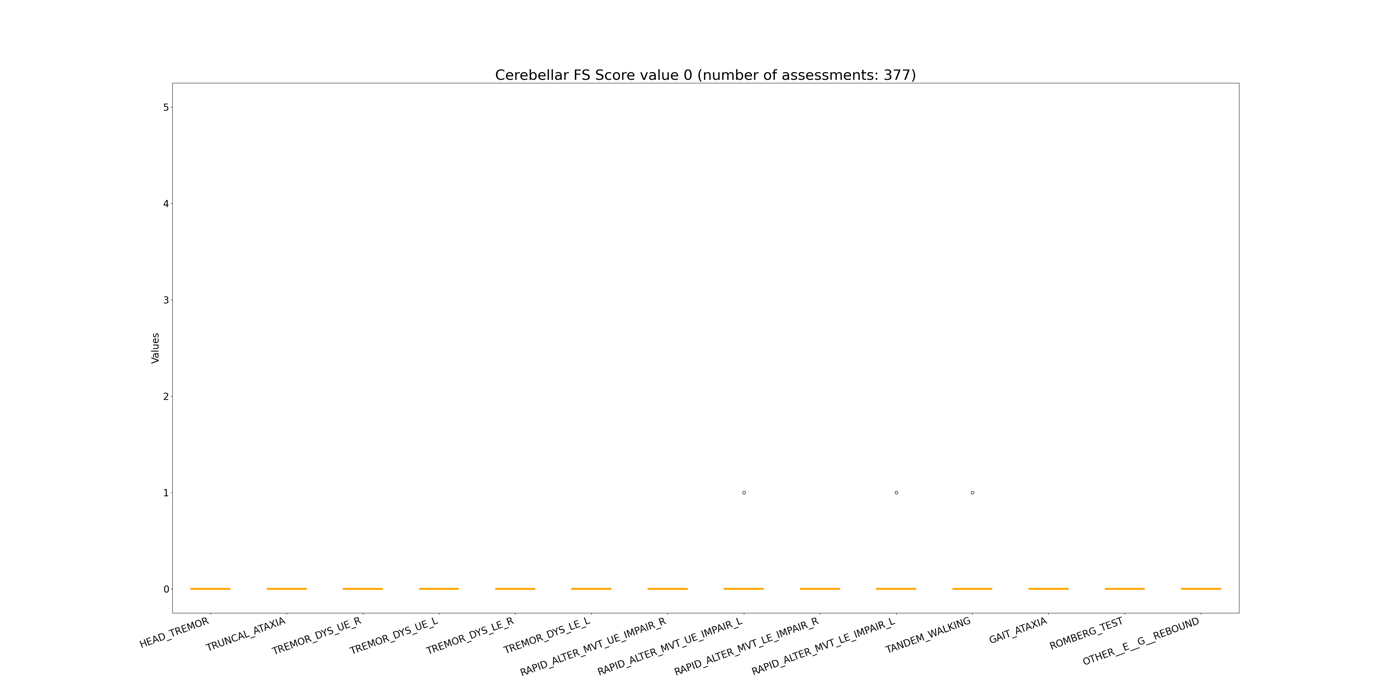

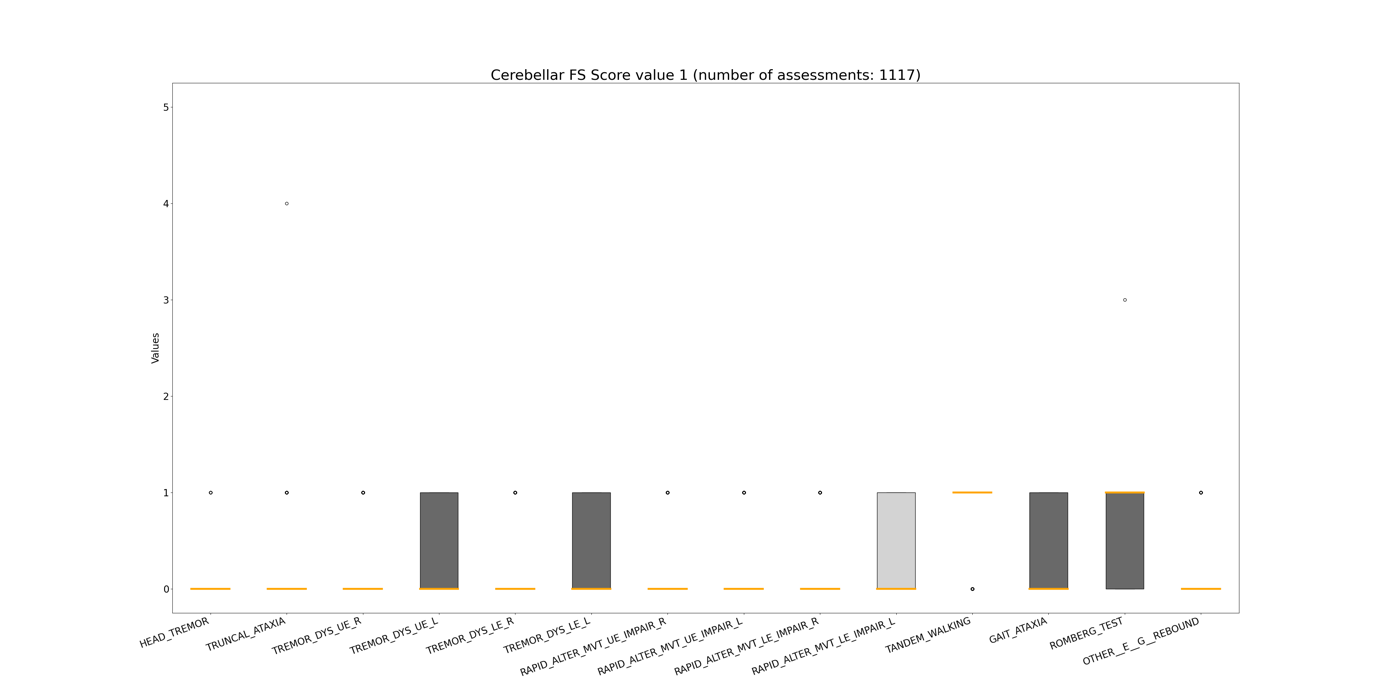


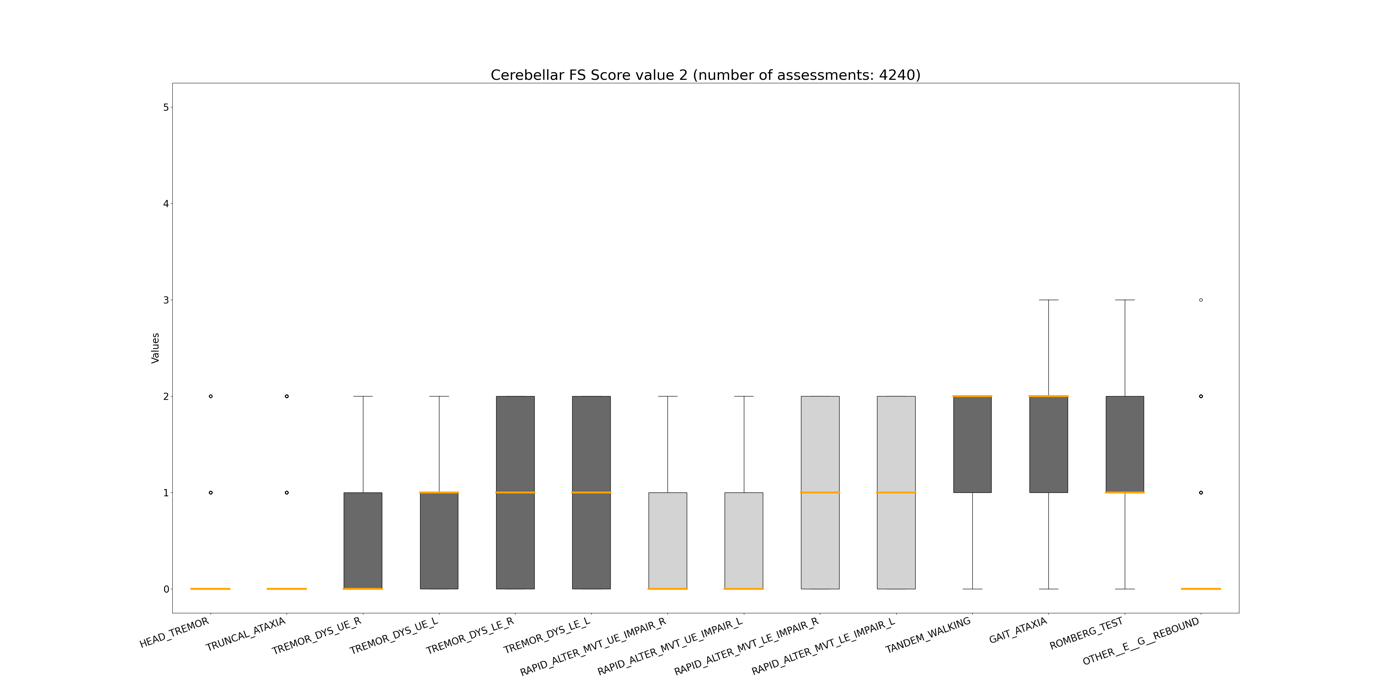


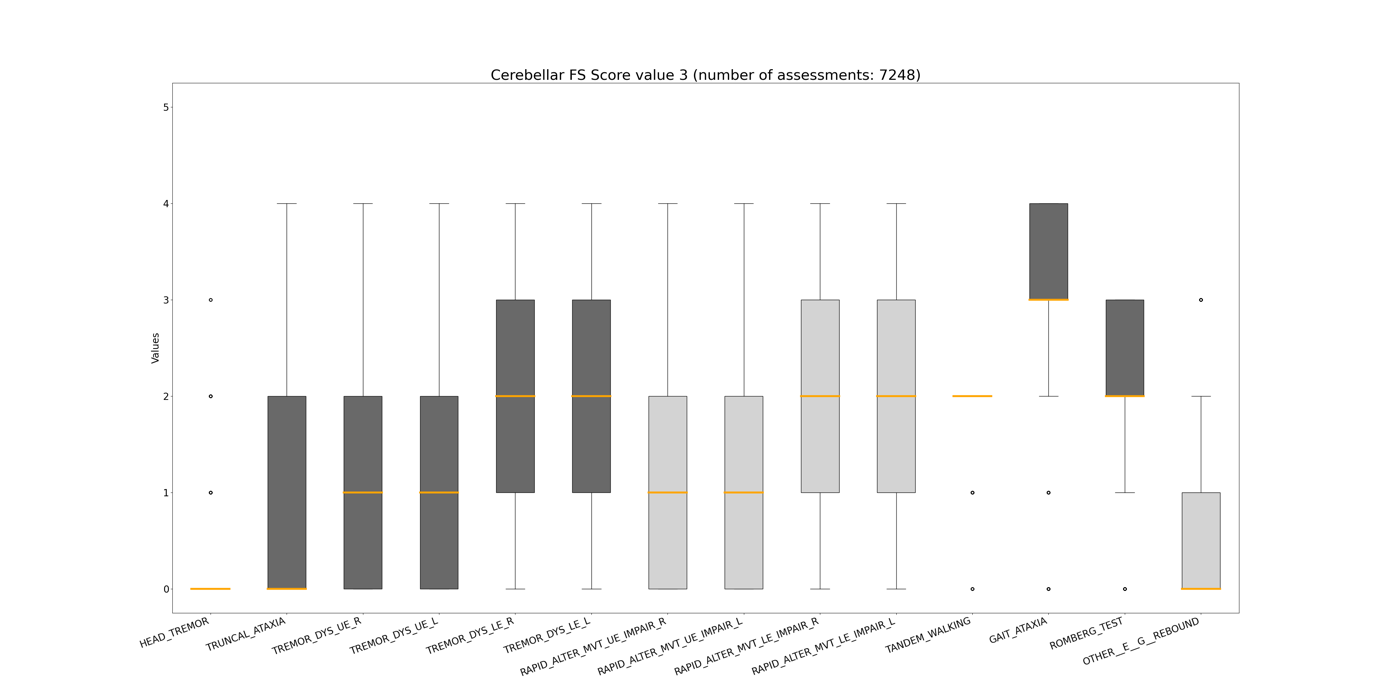

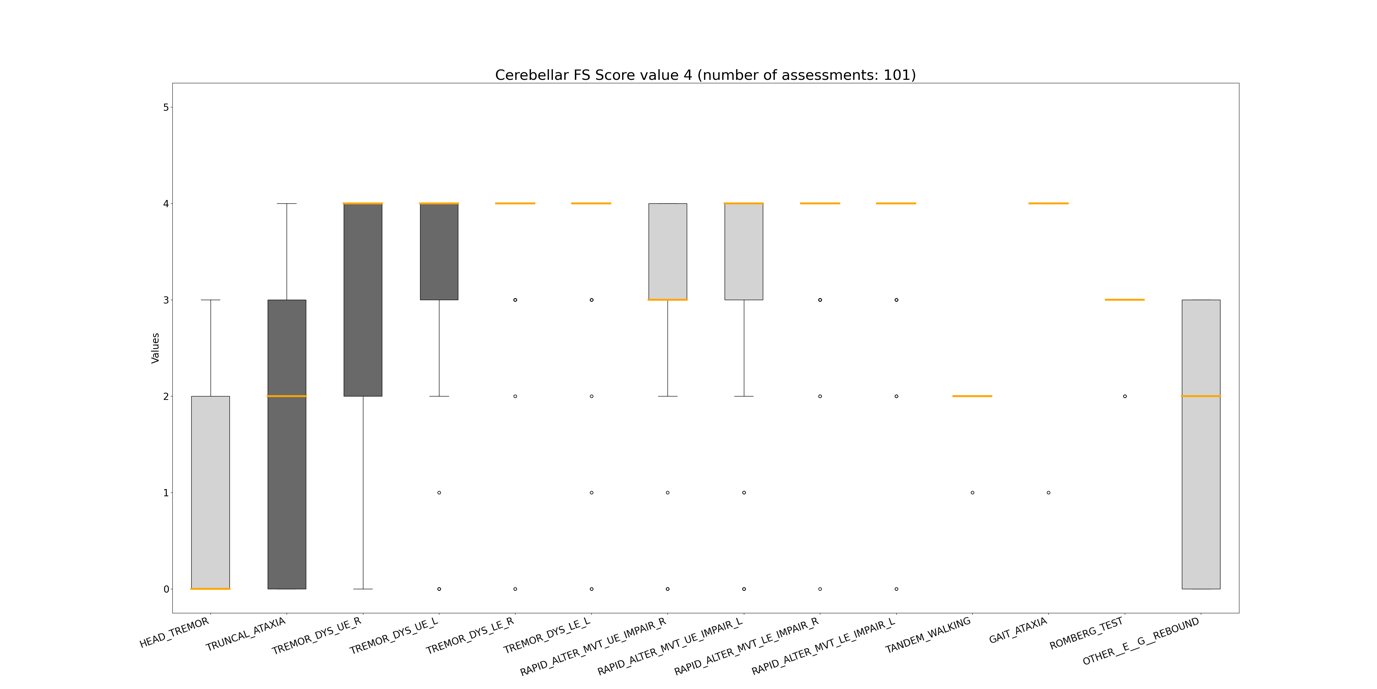

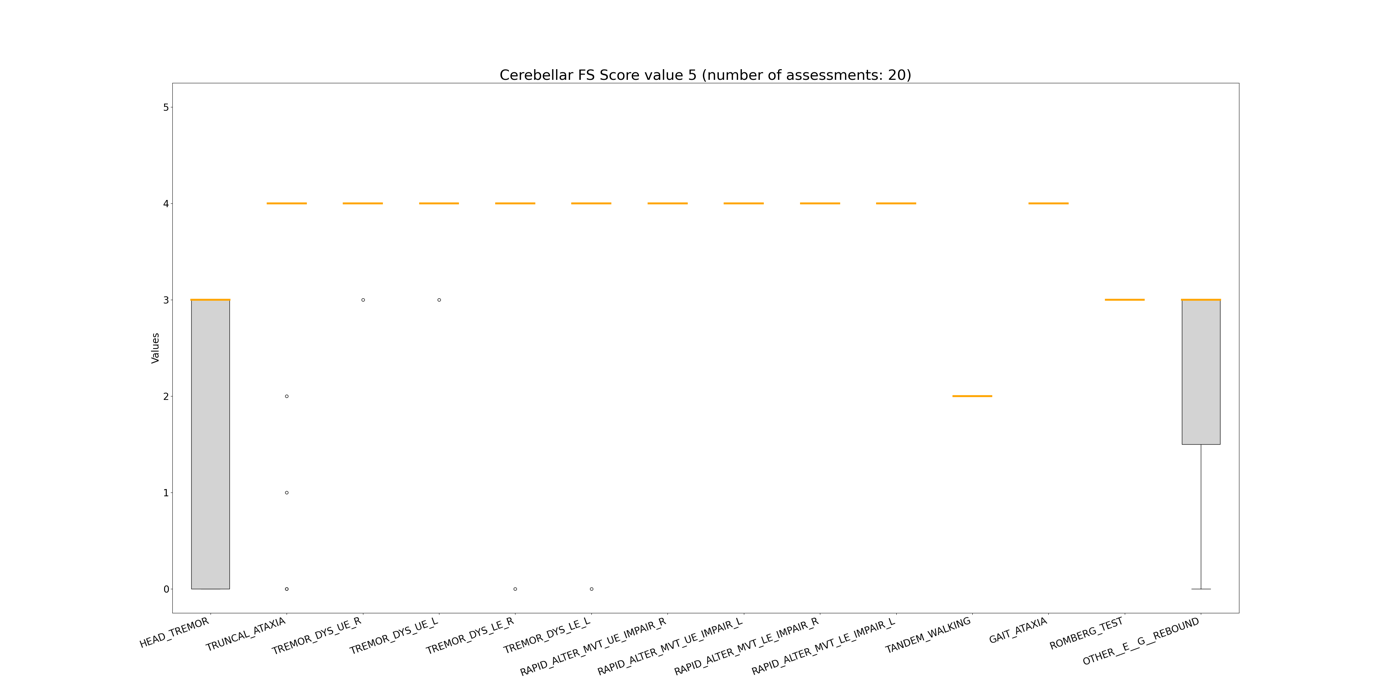


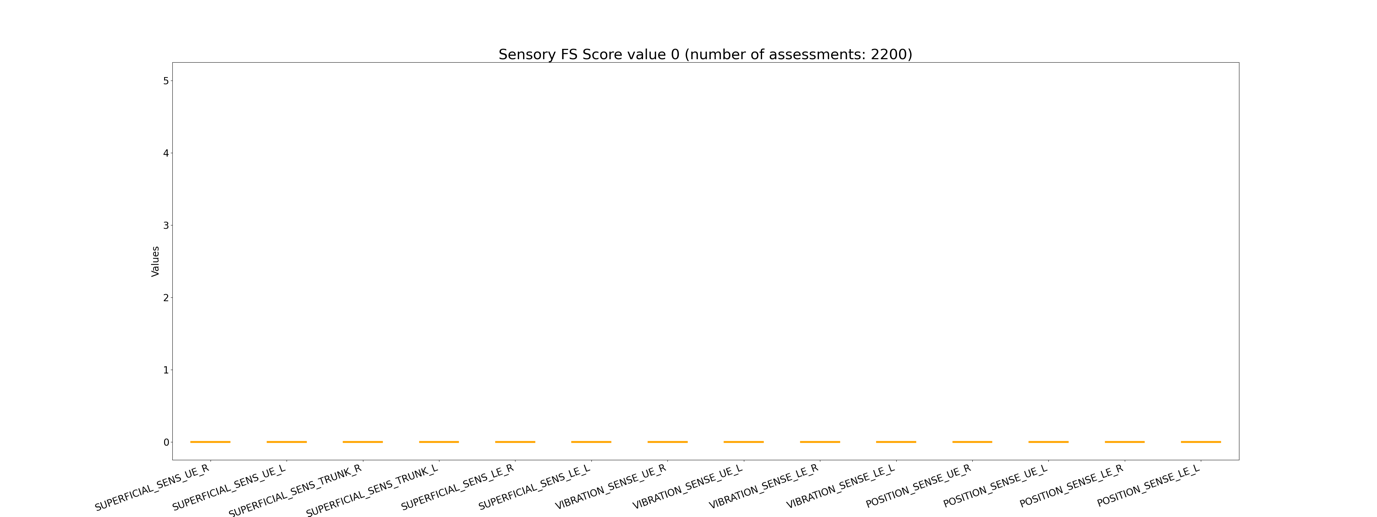


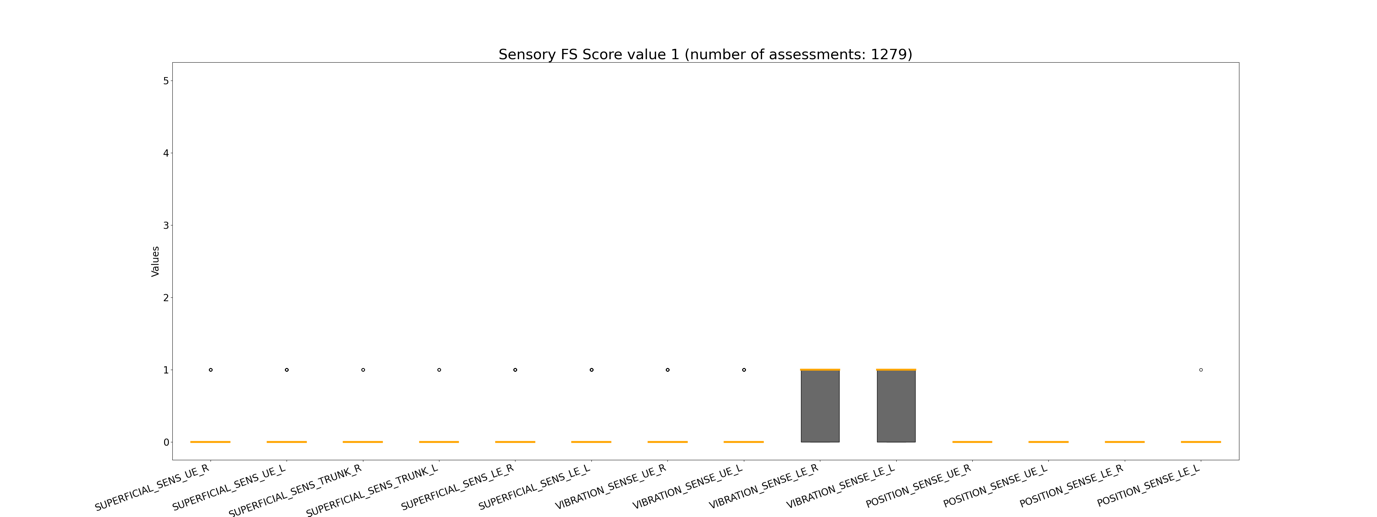

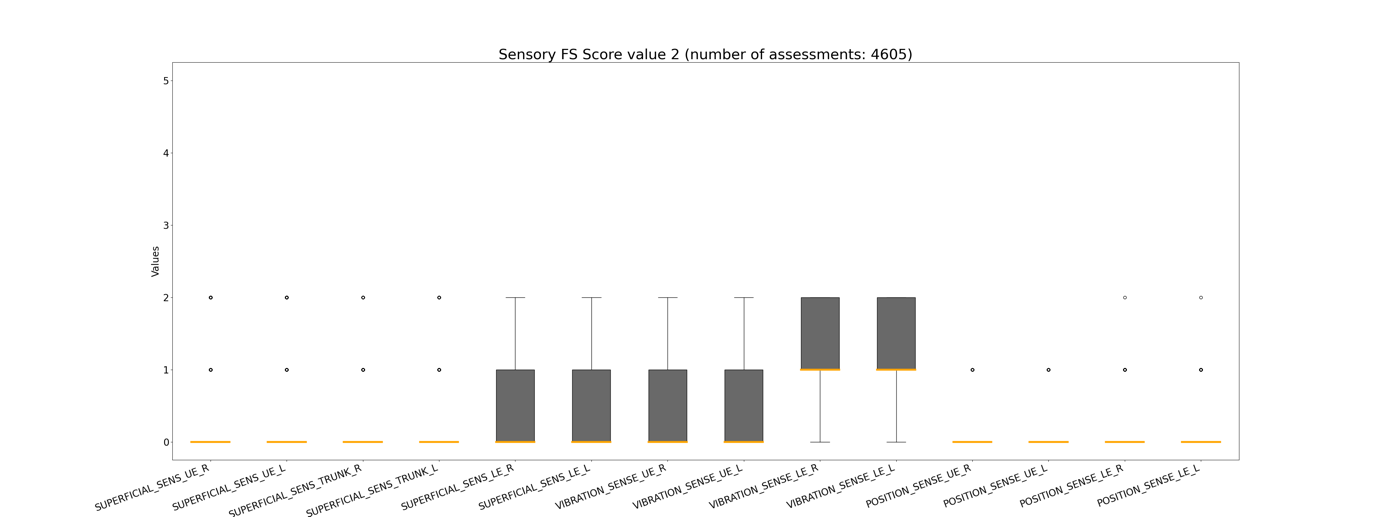

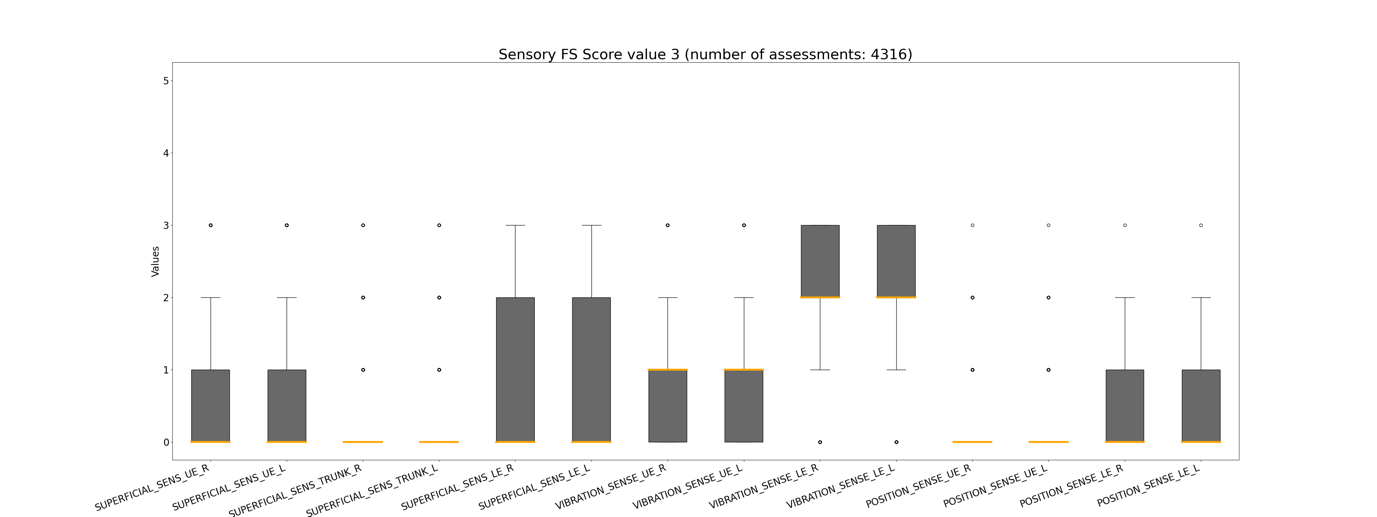

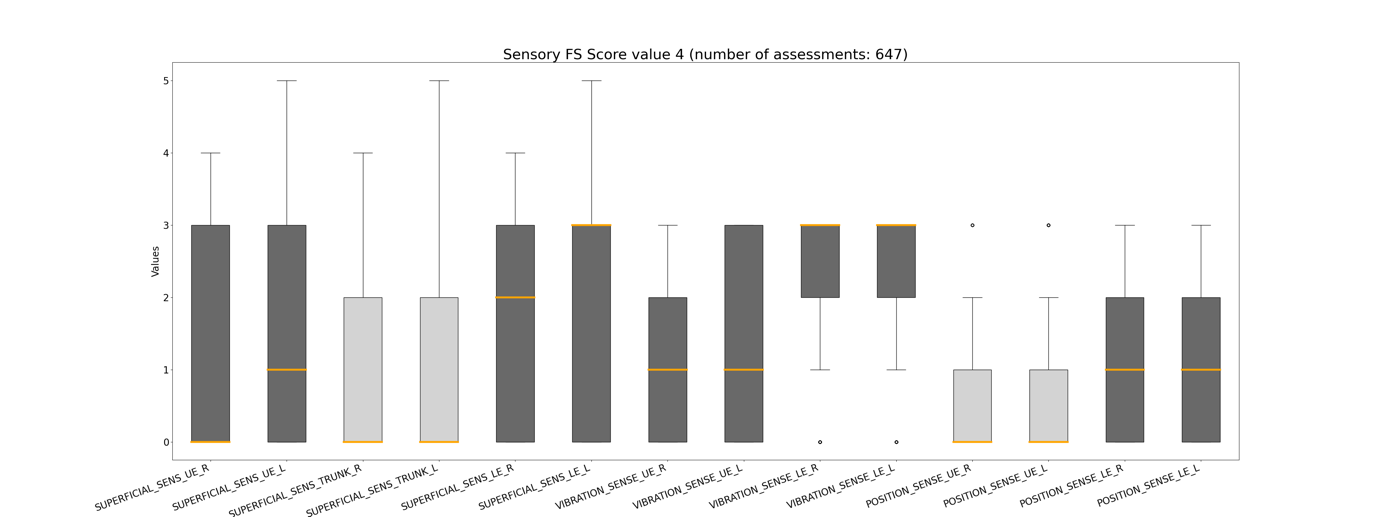

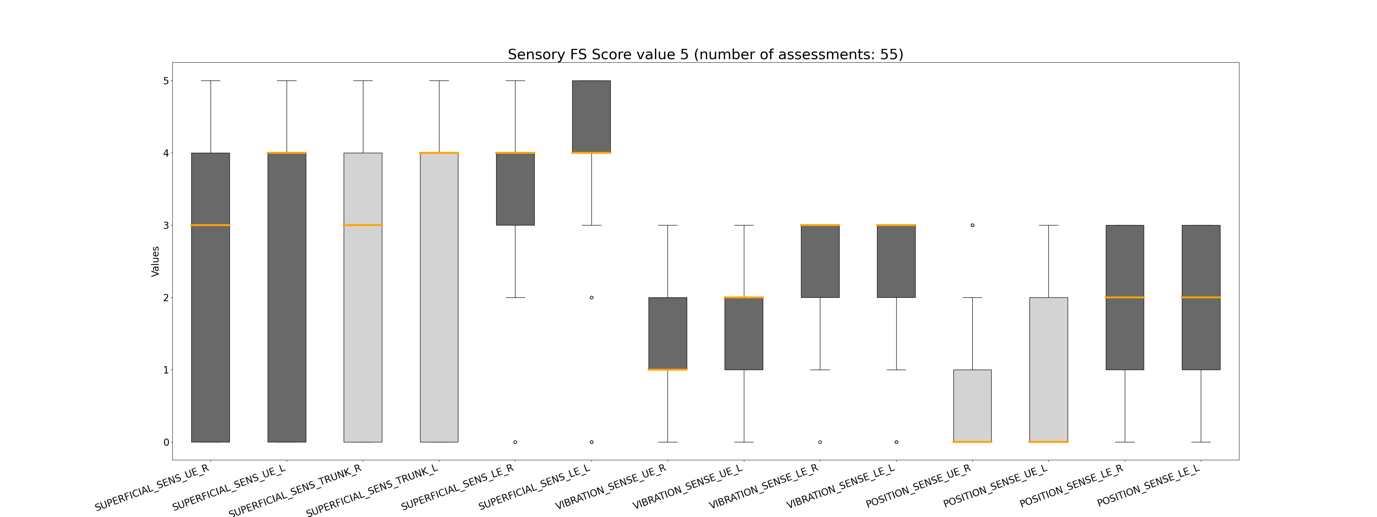

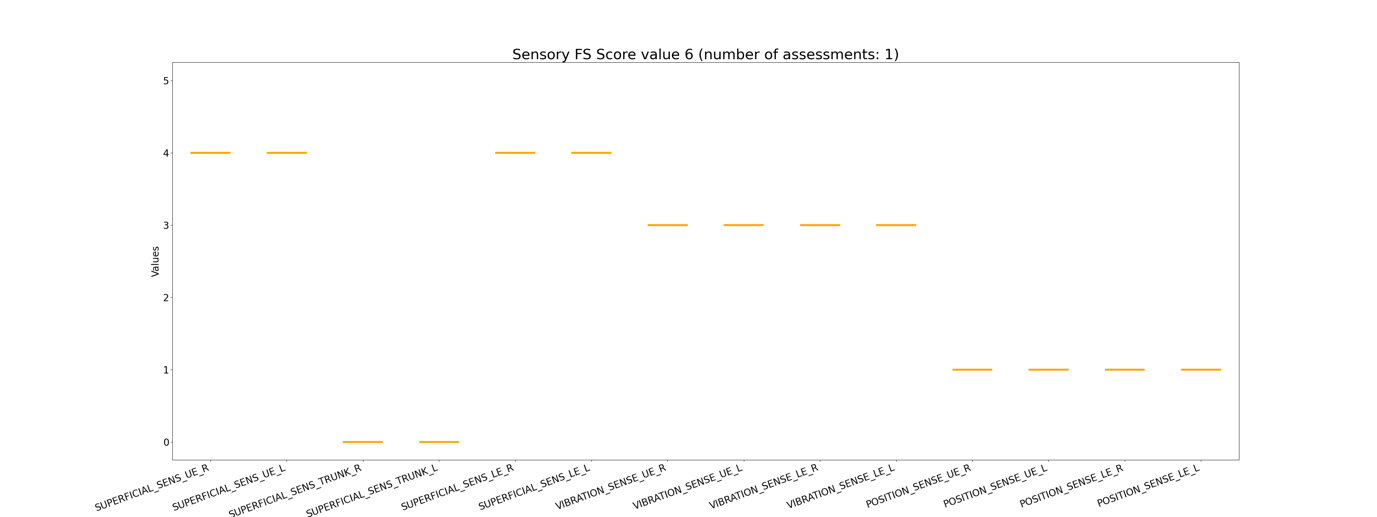


##
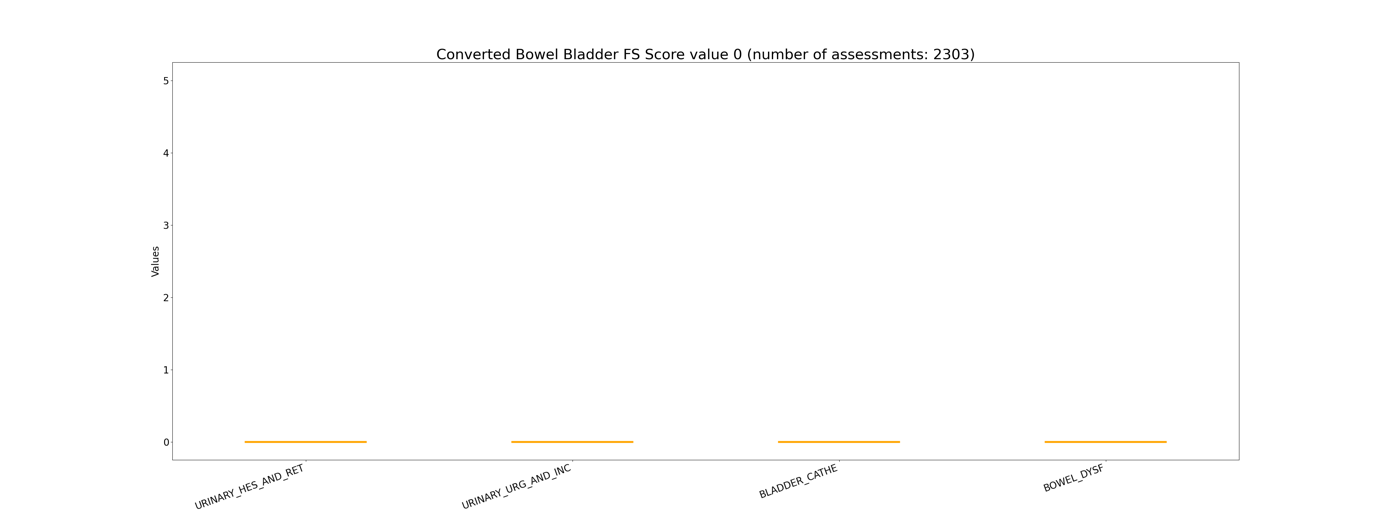

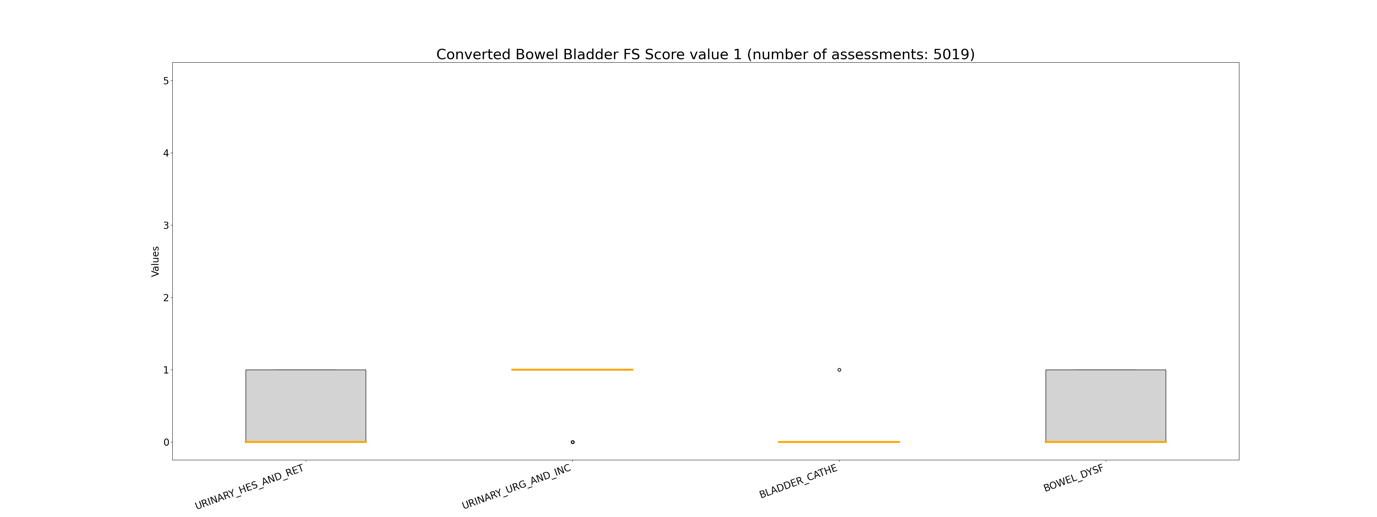

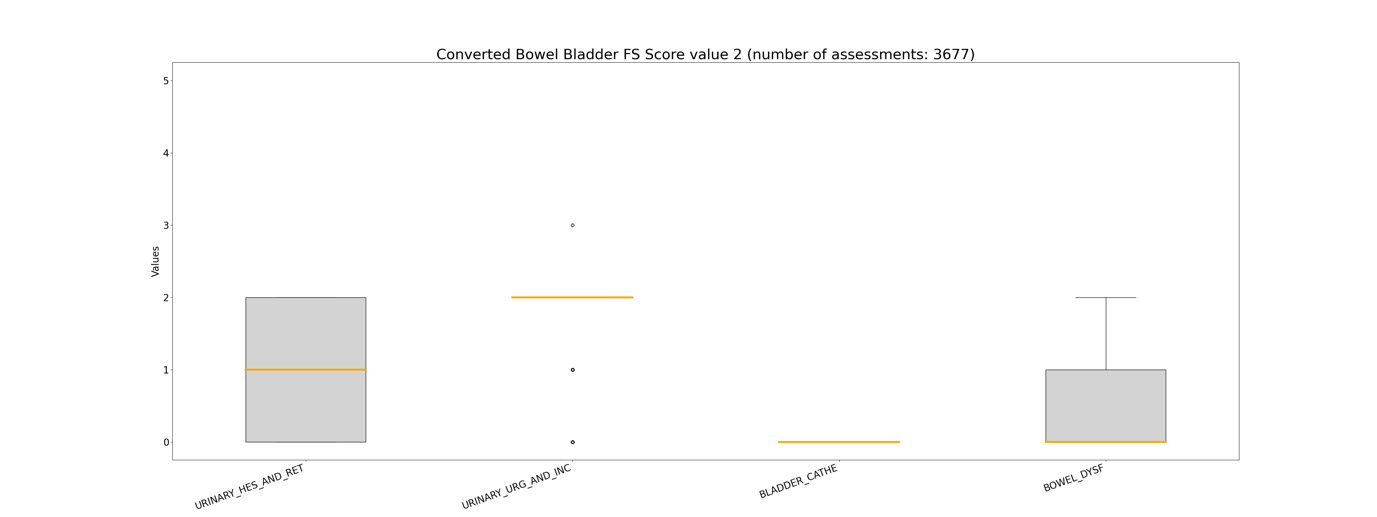

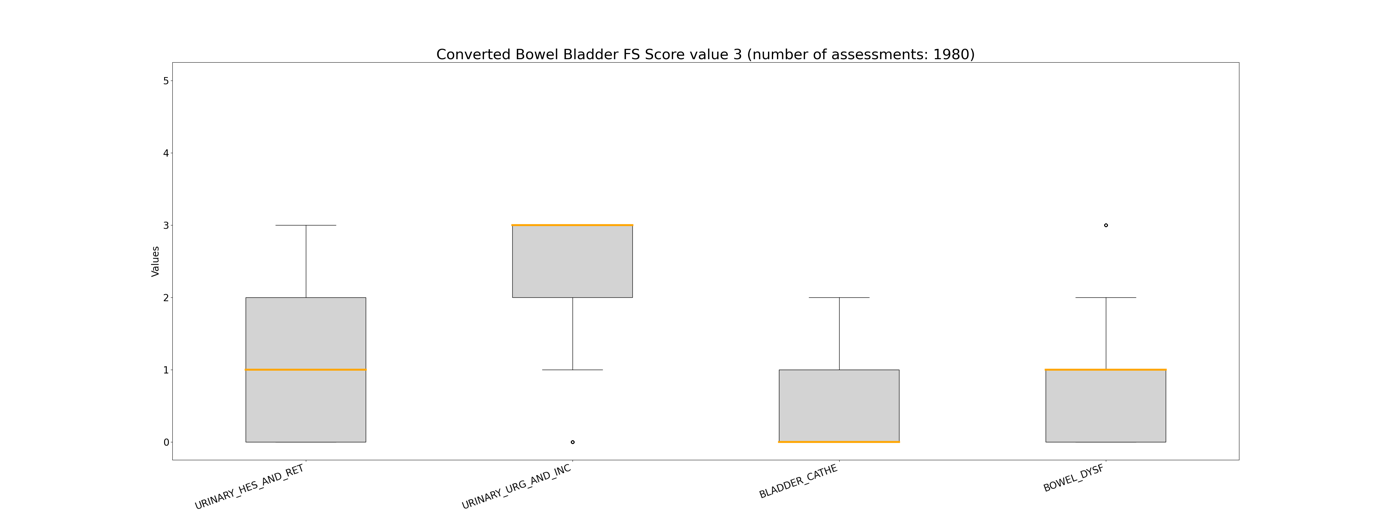

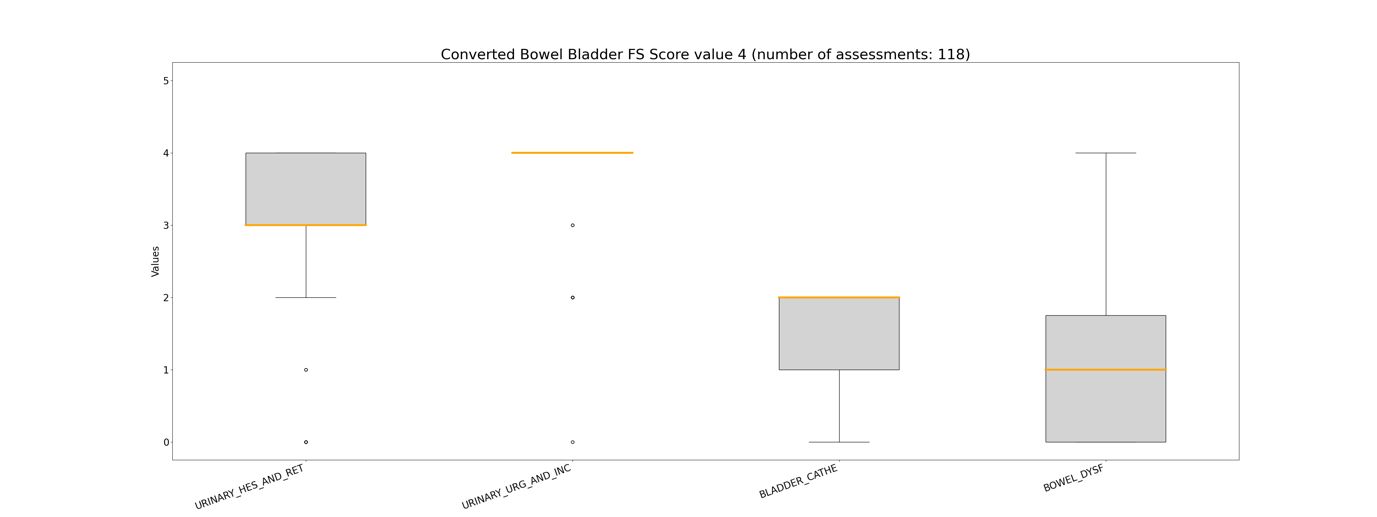

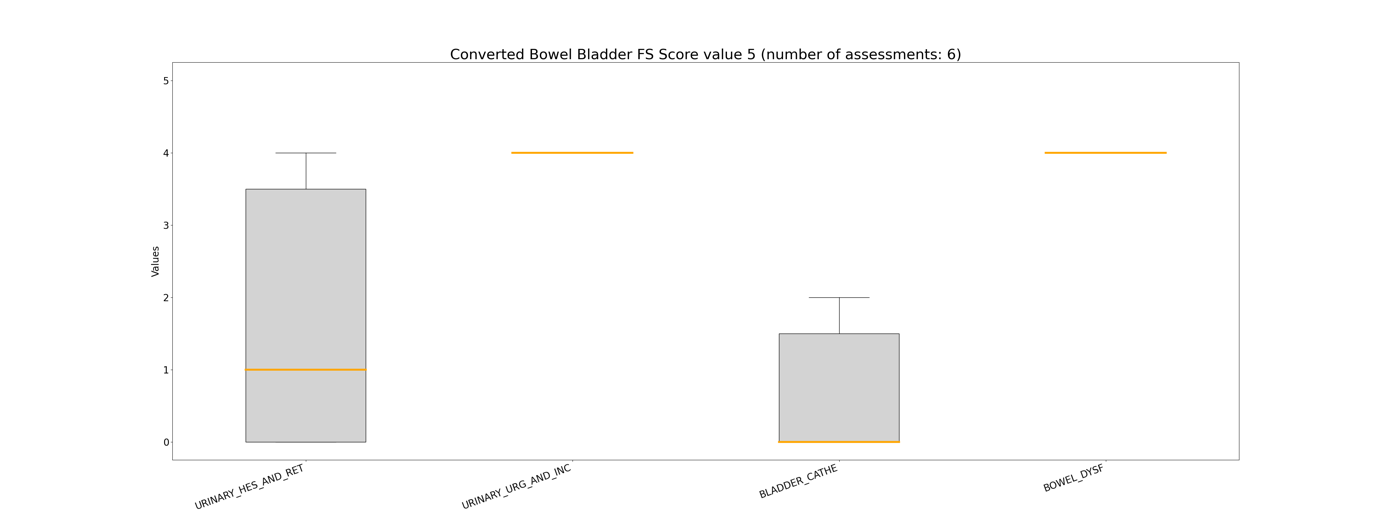


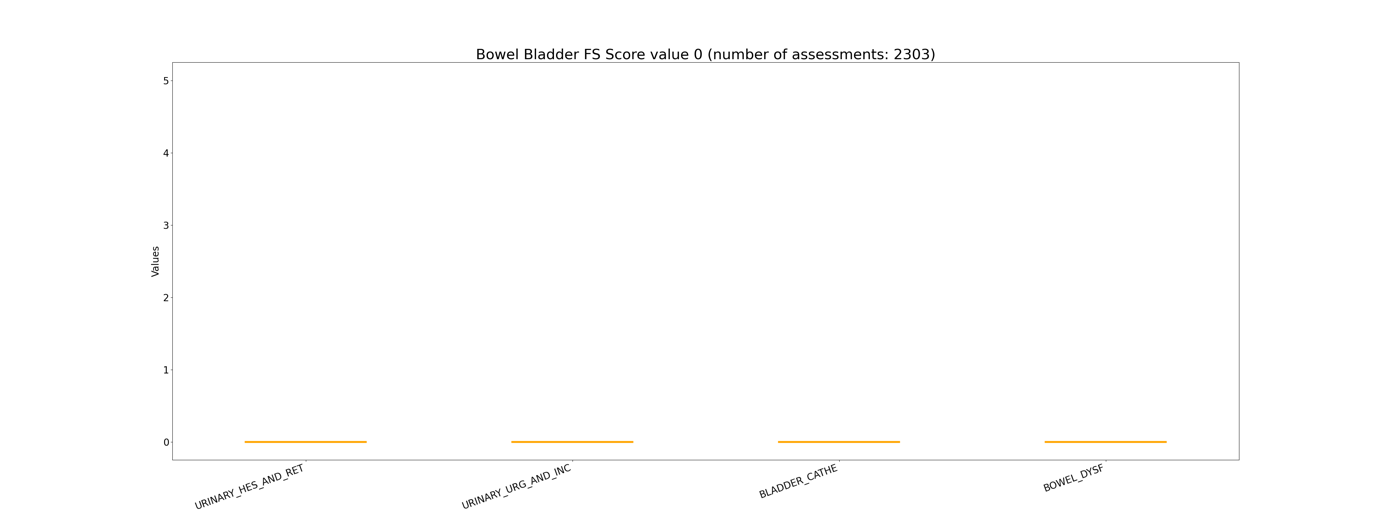

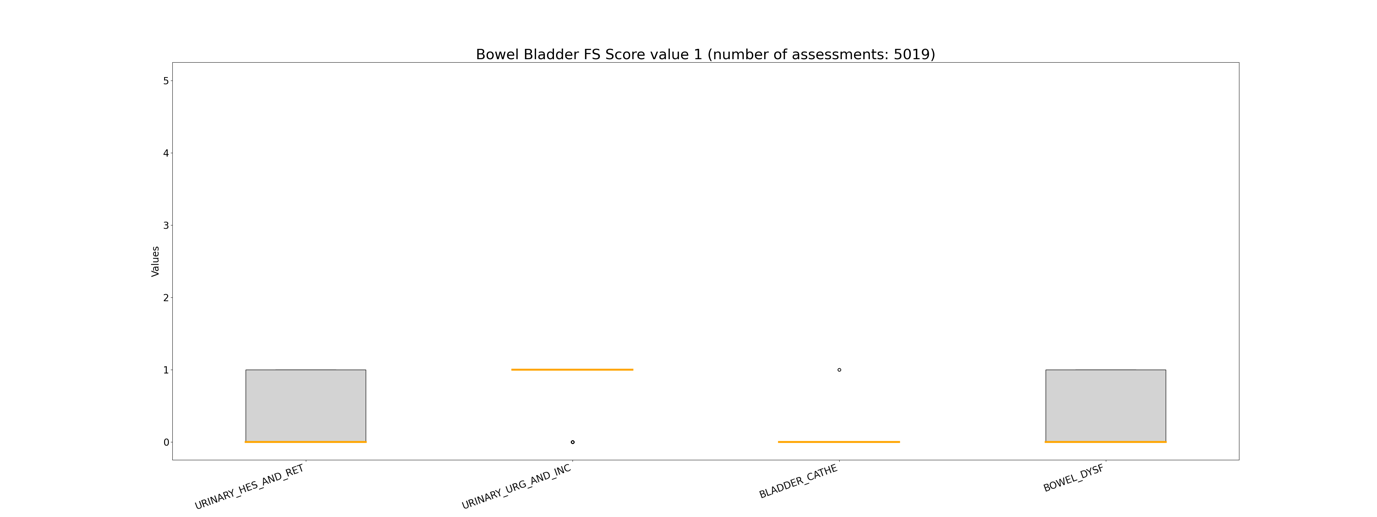

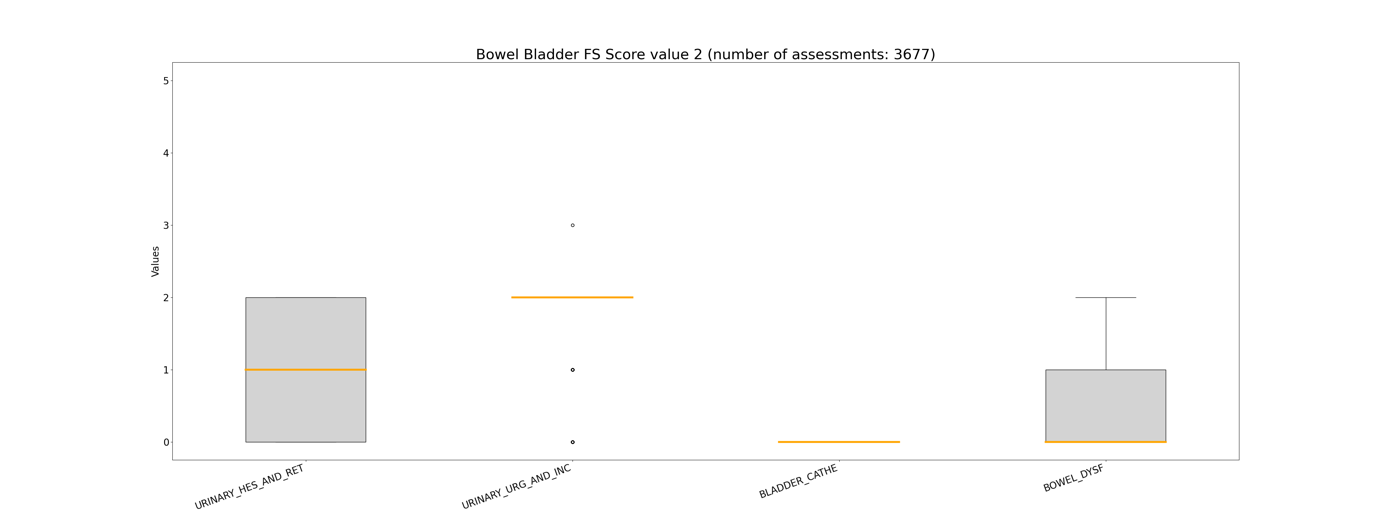


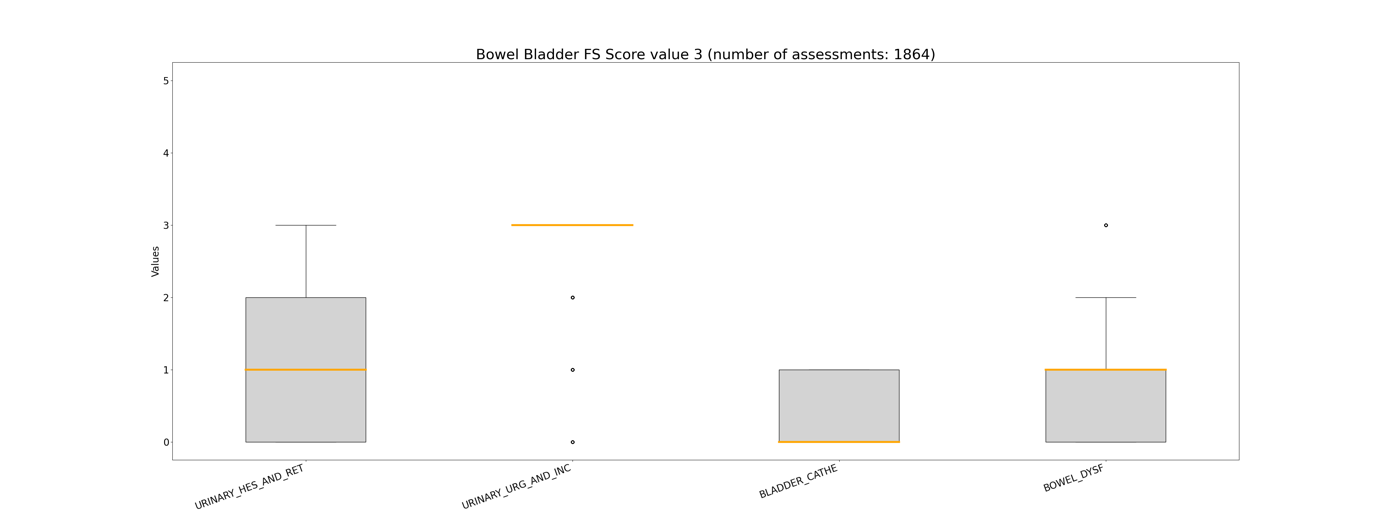


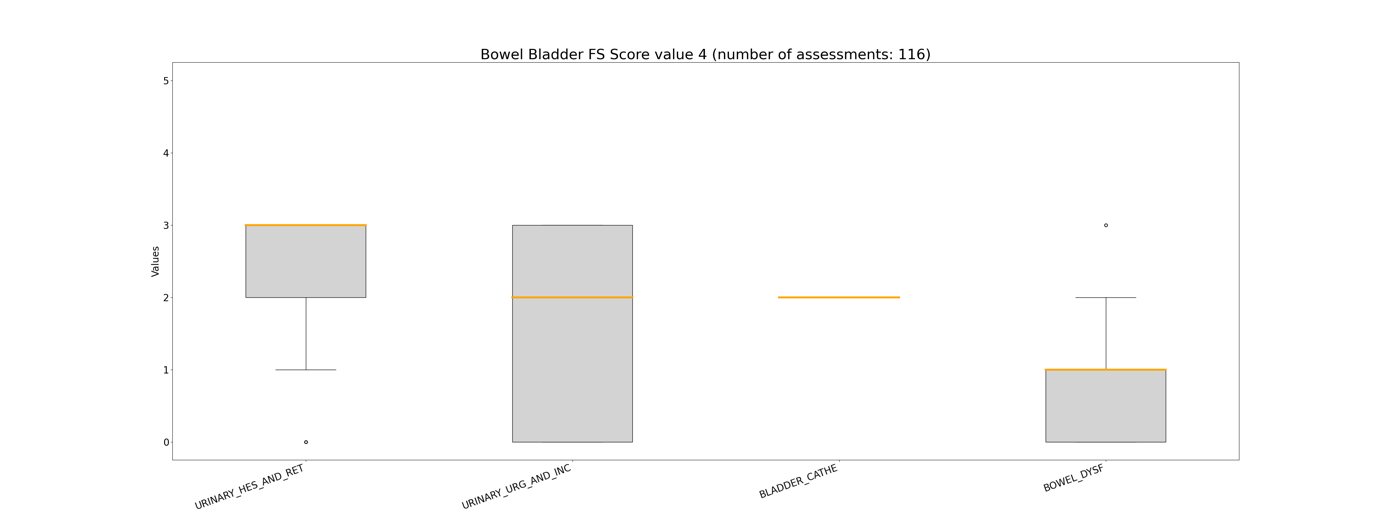

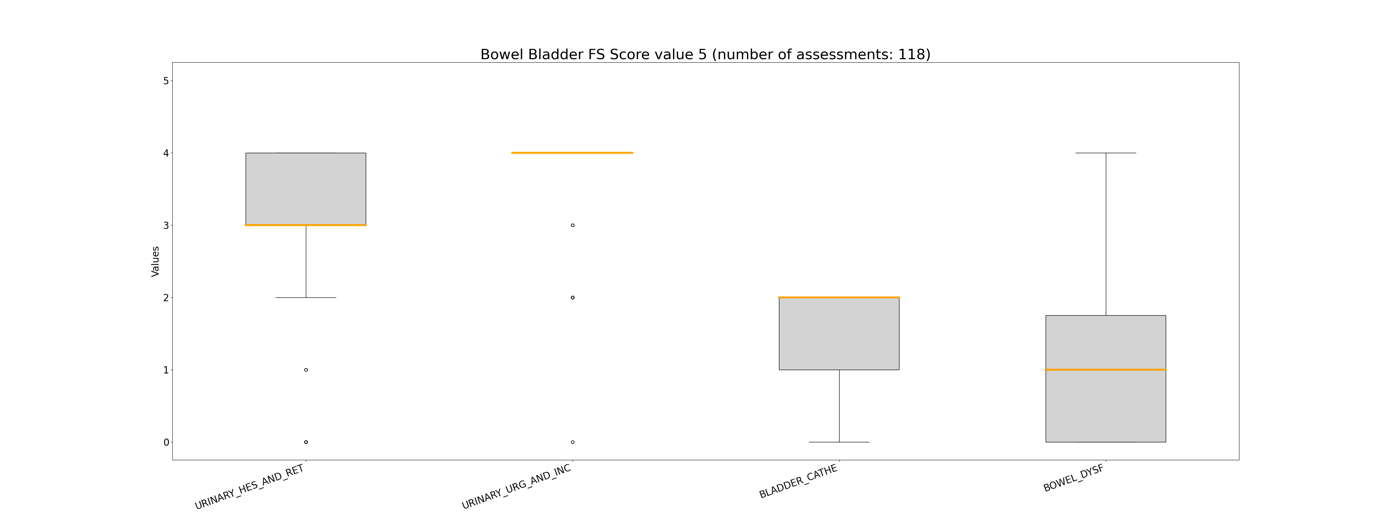

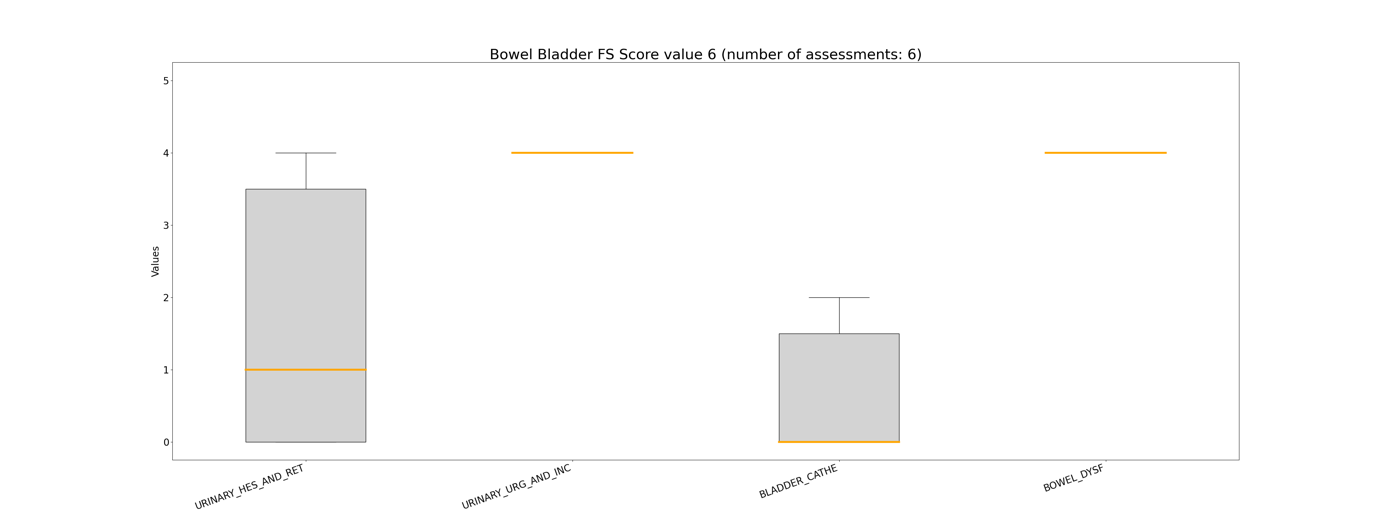


##
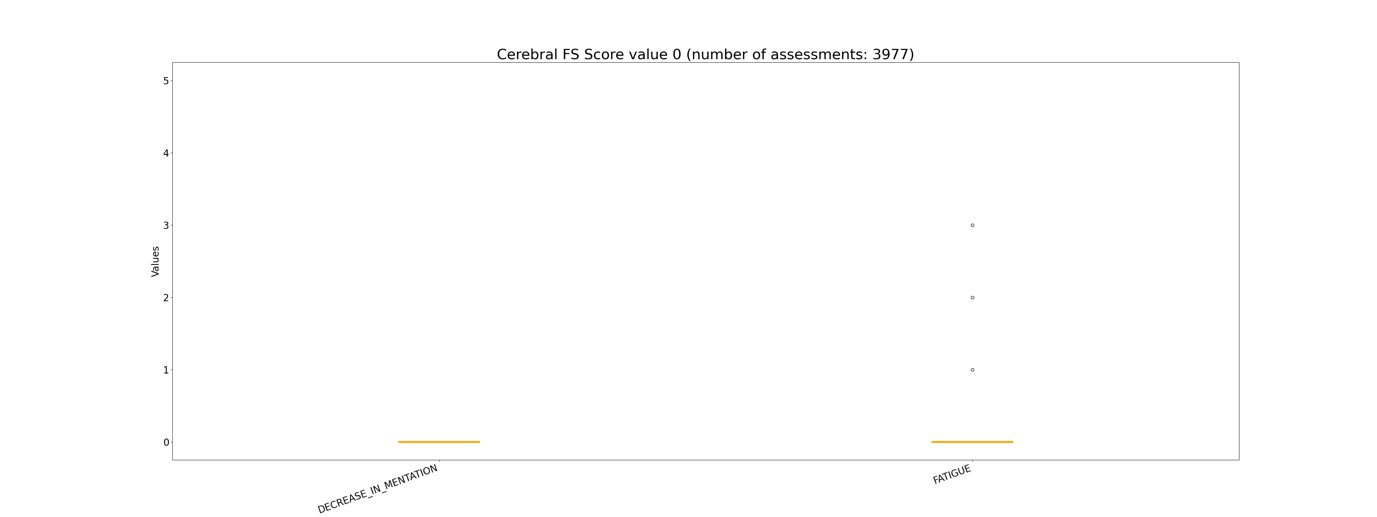

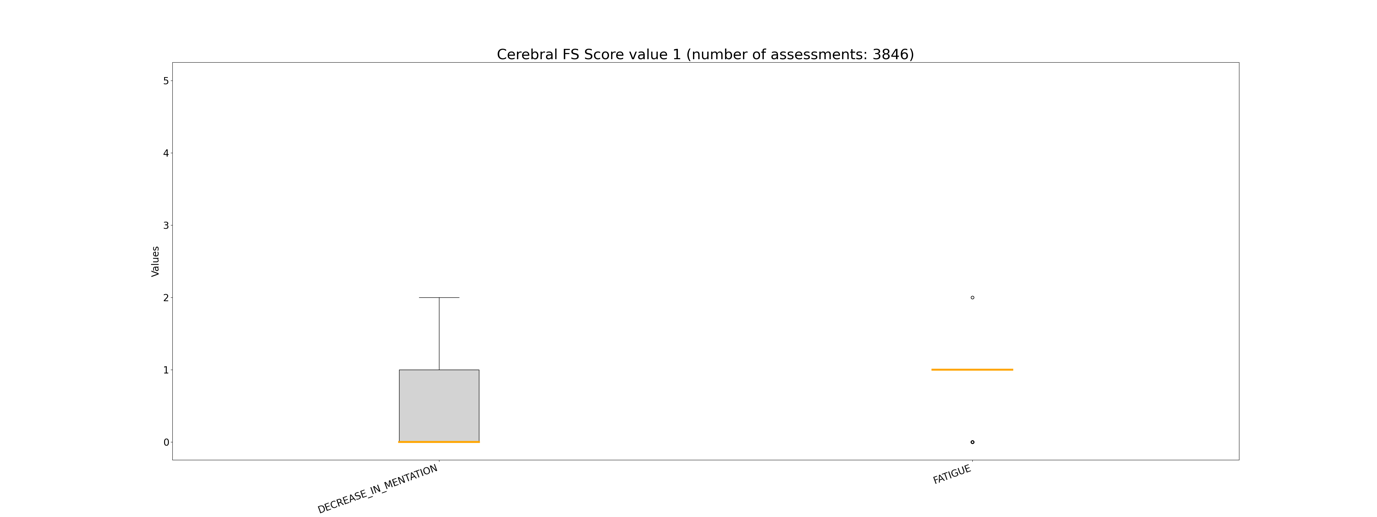

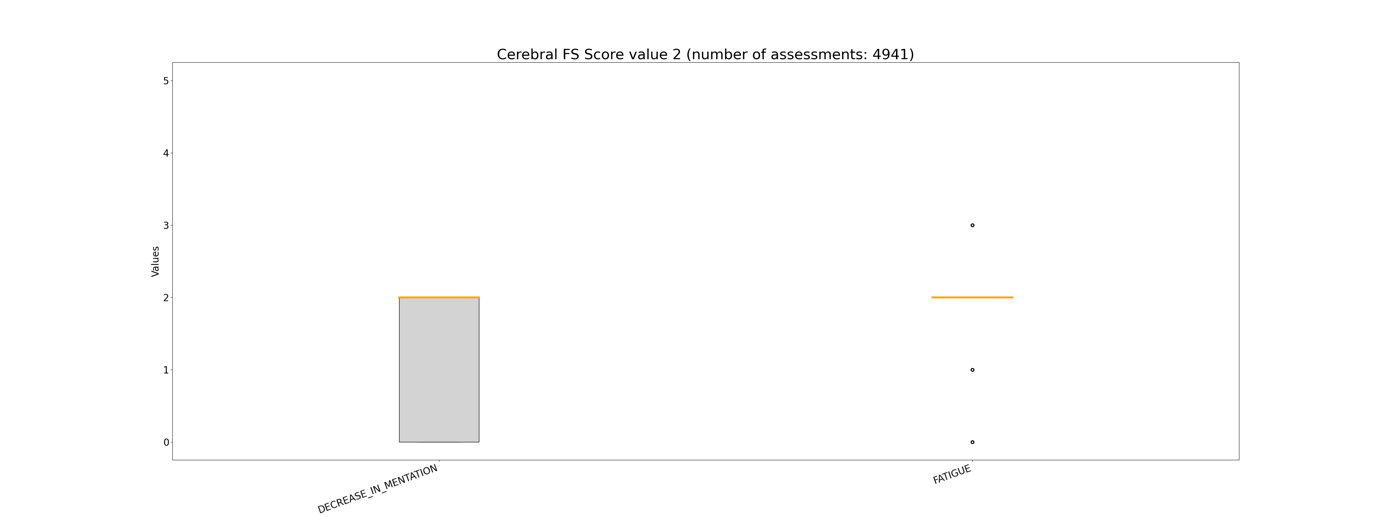

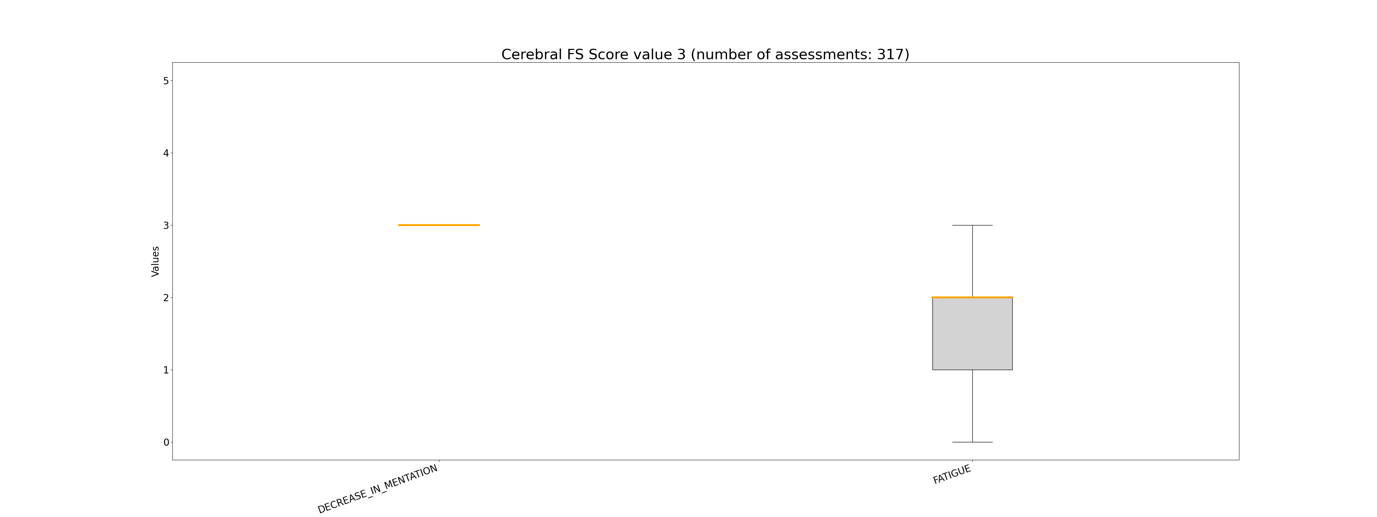

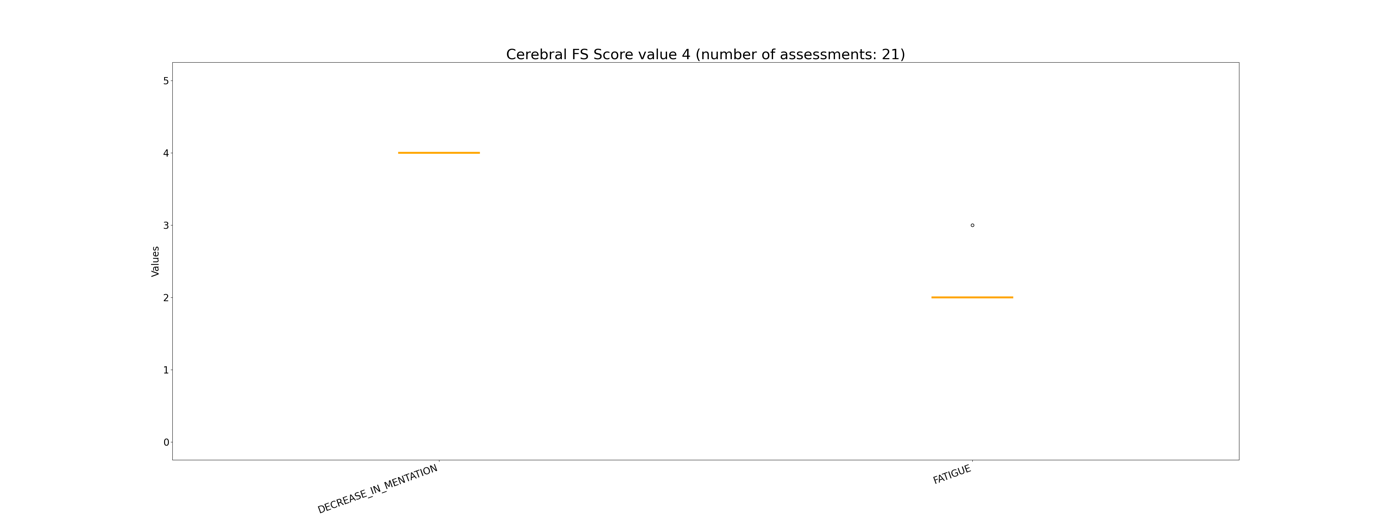

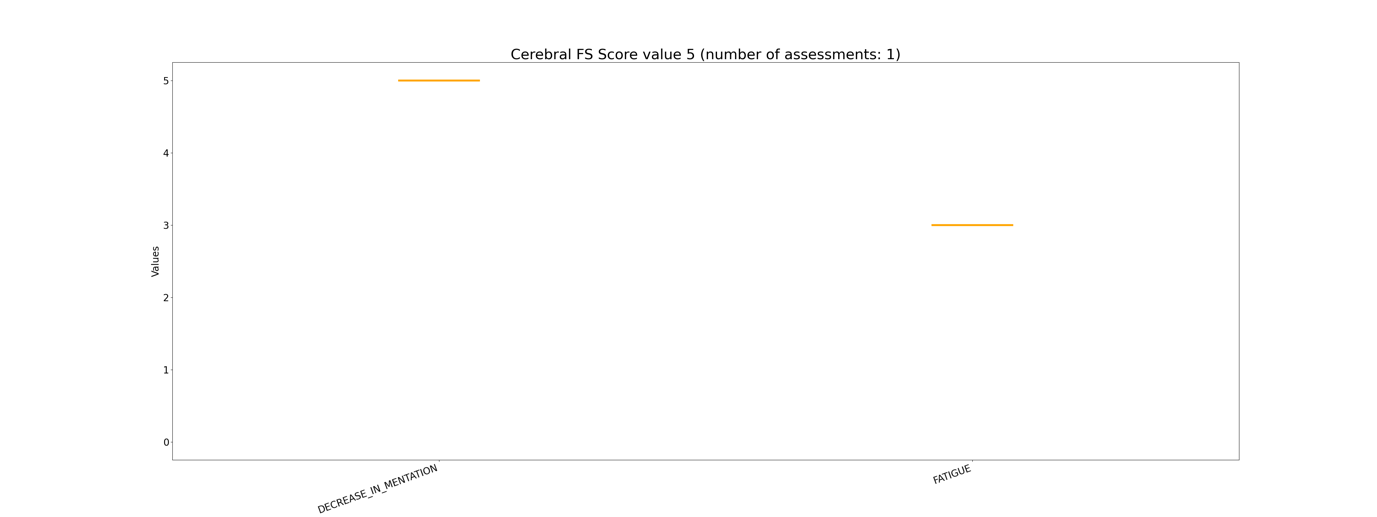


## 5.2 Distribution of Subscores for each EDSS

The distribution of each subscore is presented for every EDSS step. Subscores highlighted in dark grey are those included in the cluster analysis.


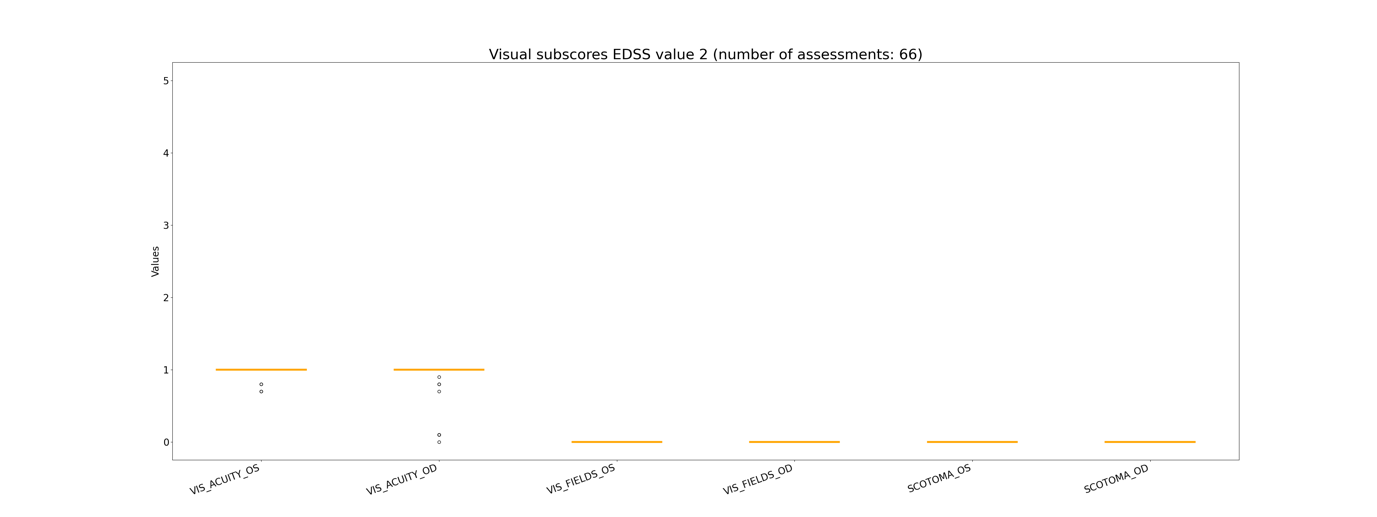

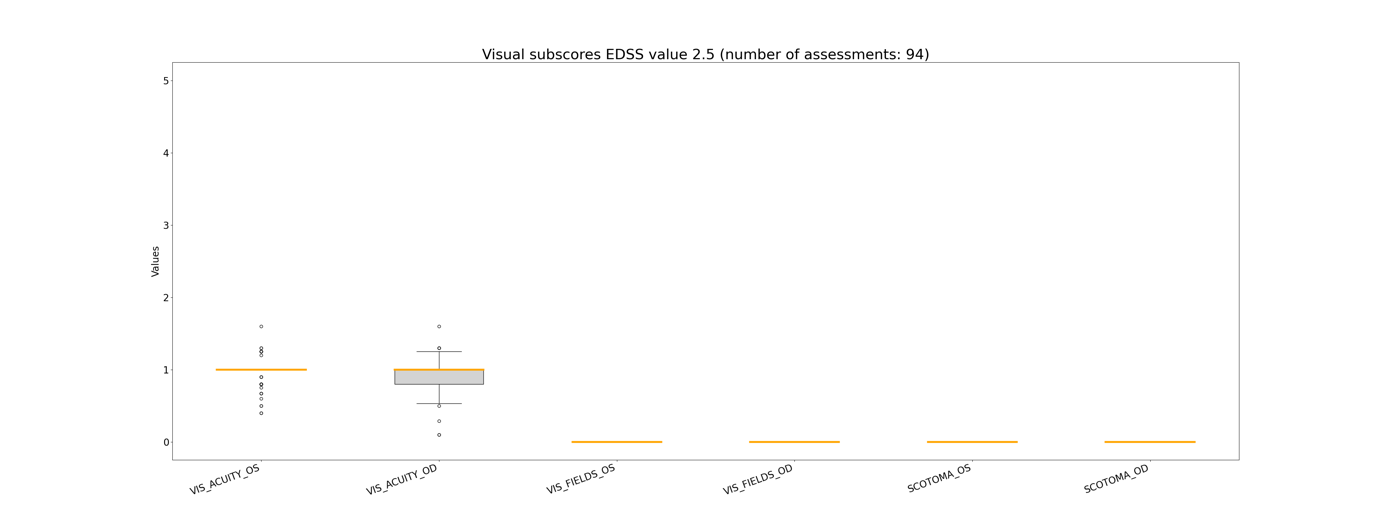

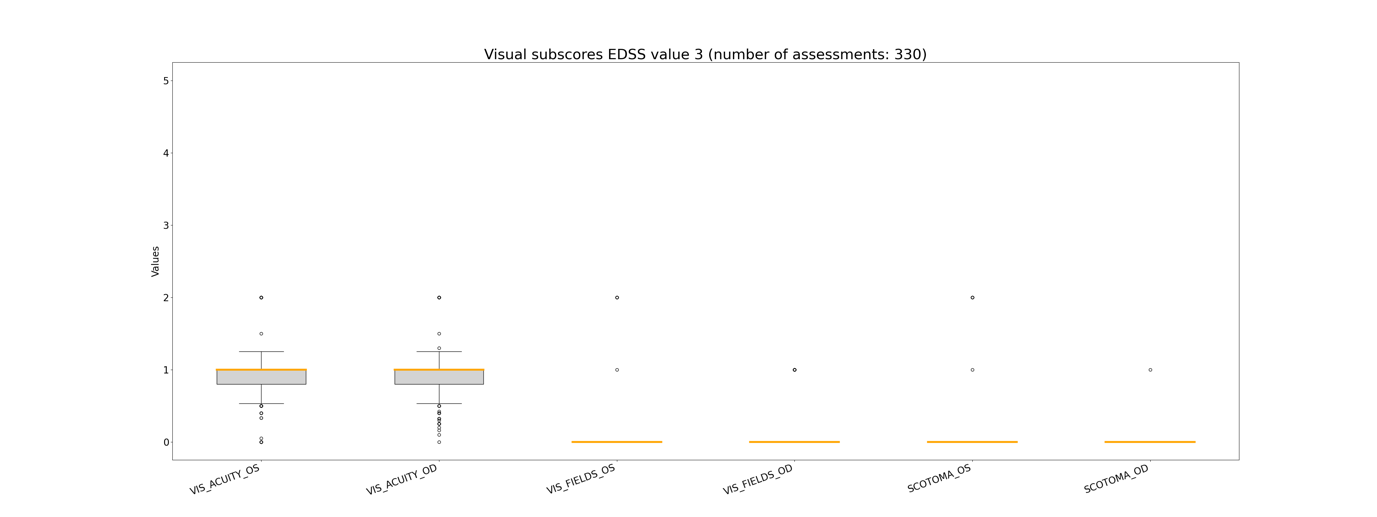

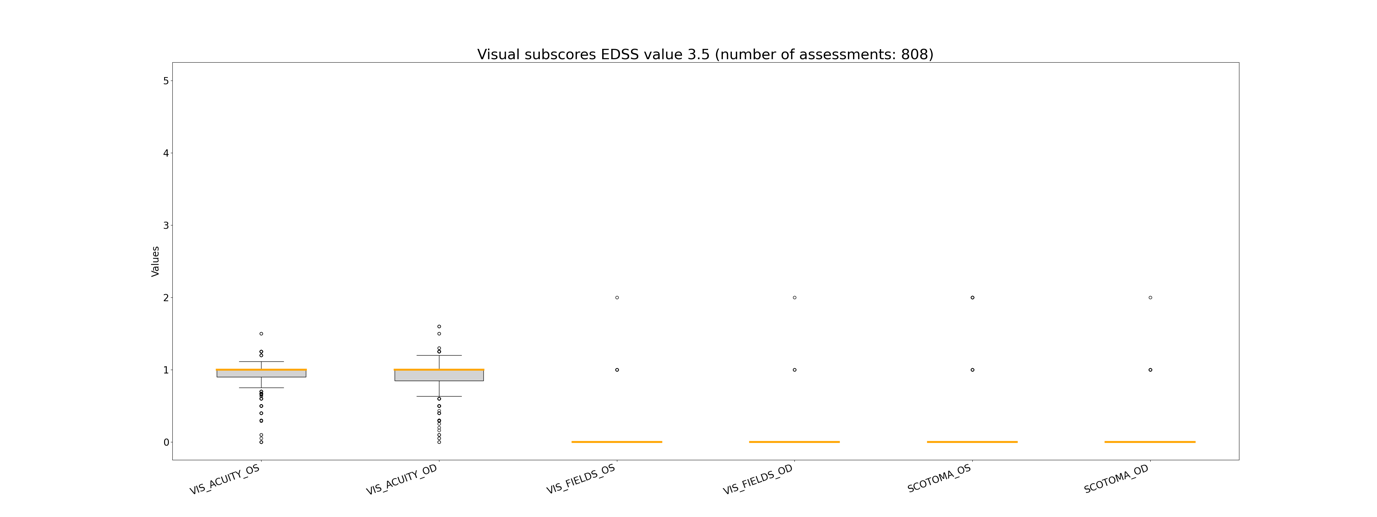

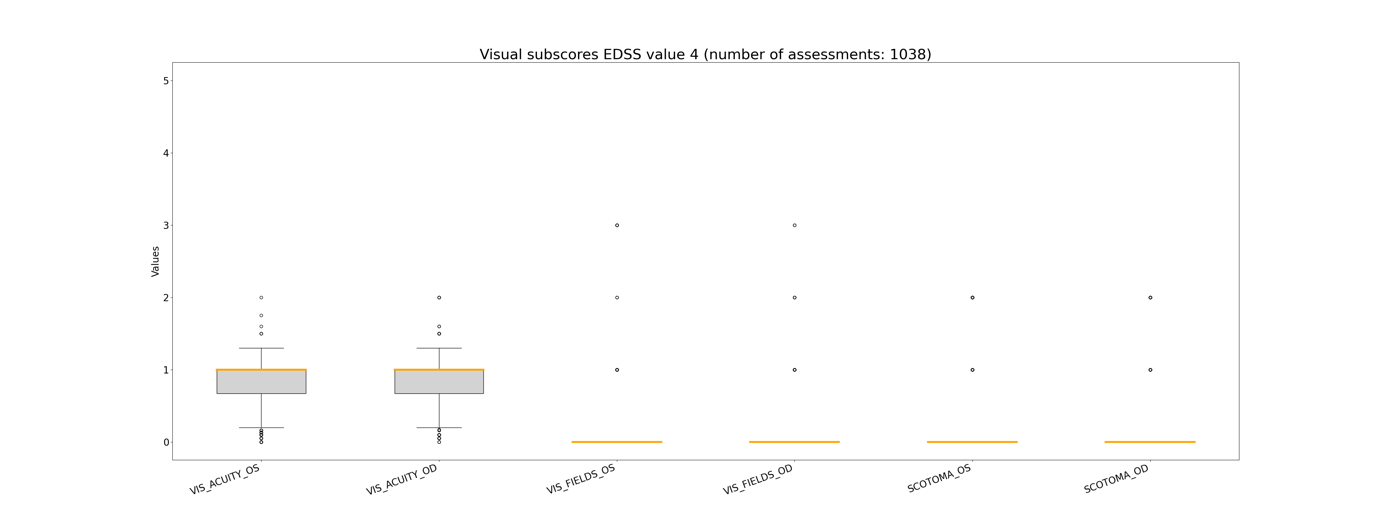

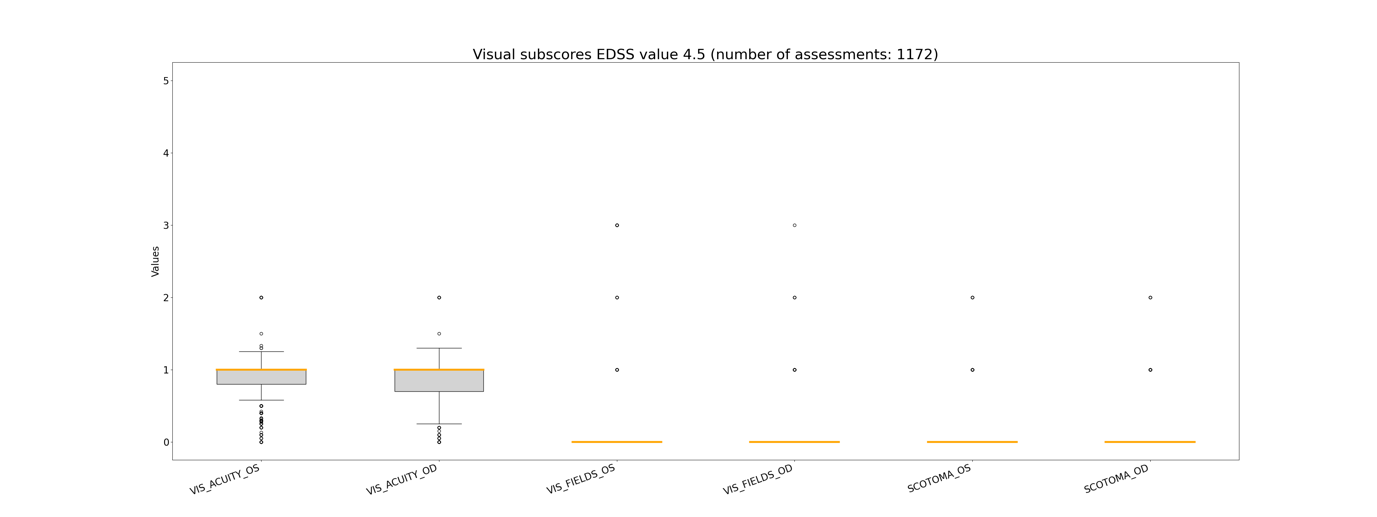

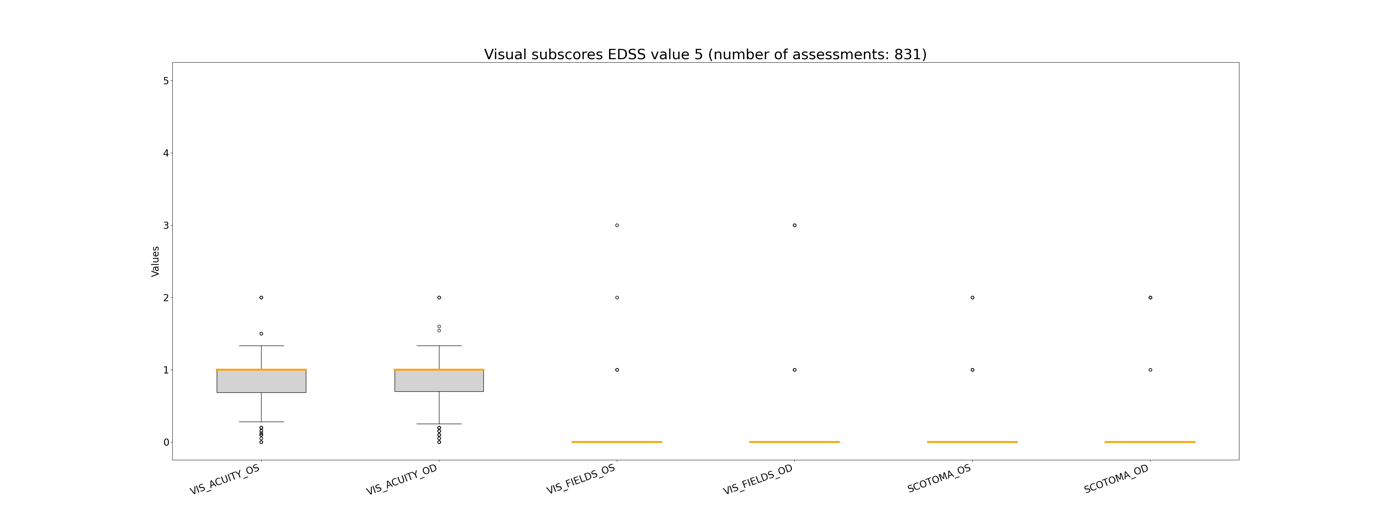

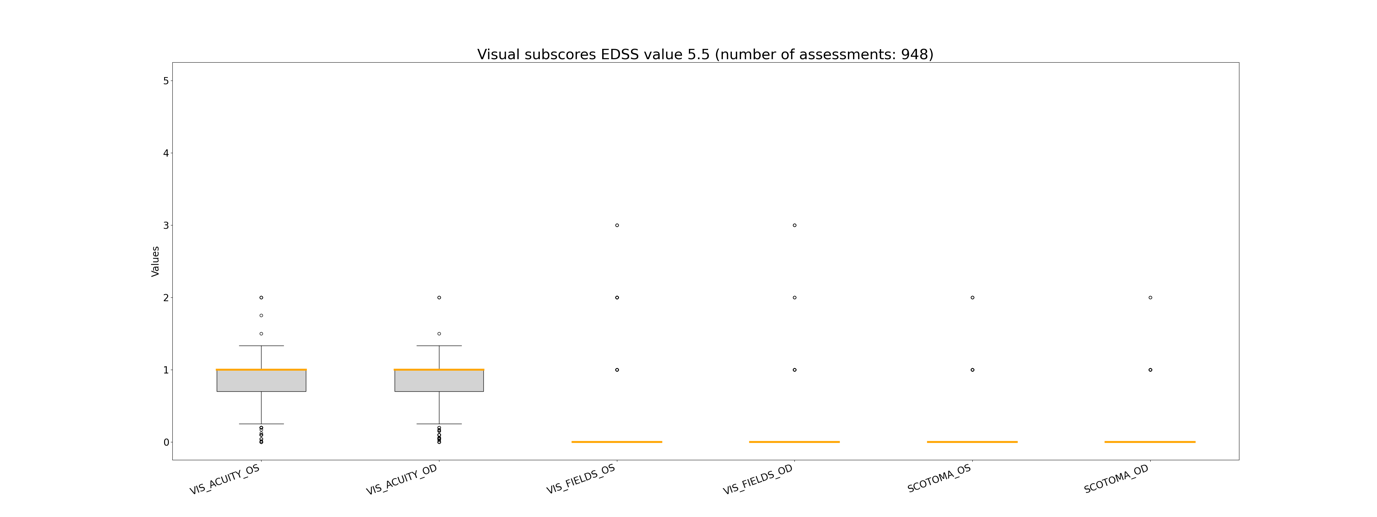

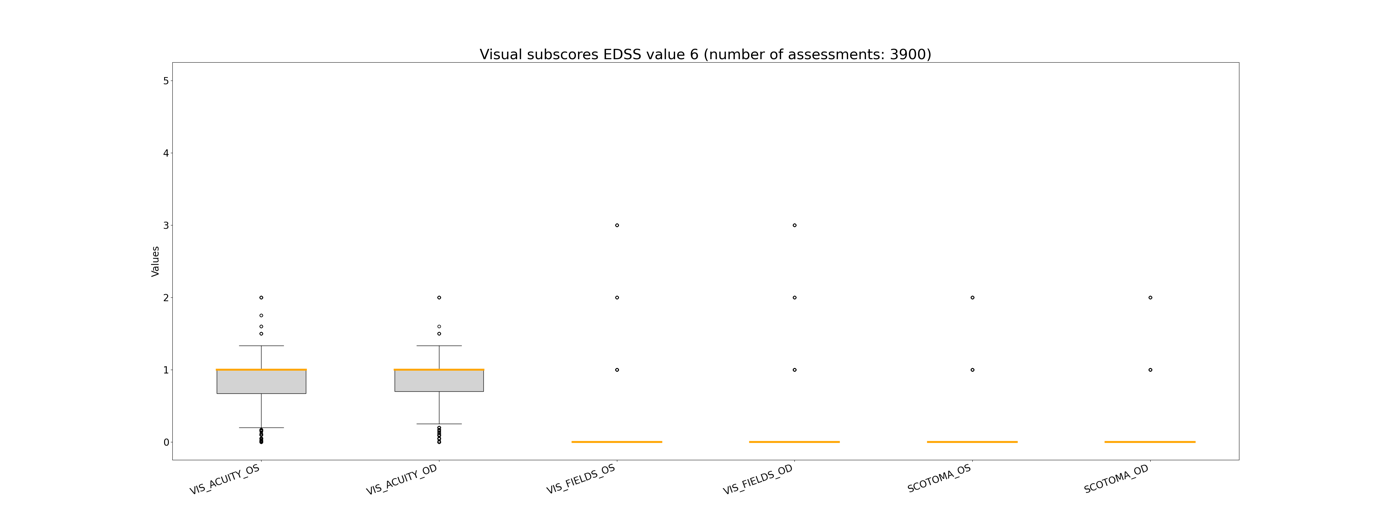

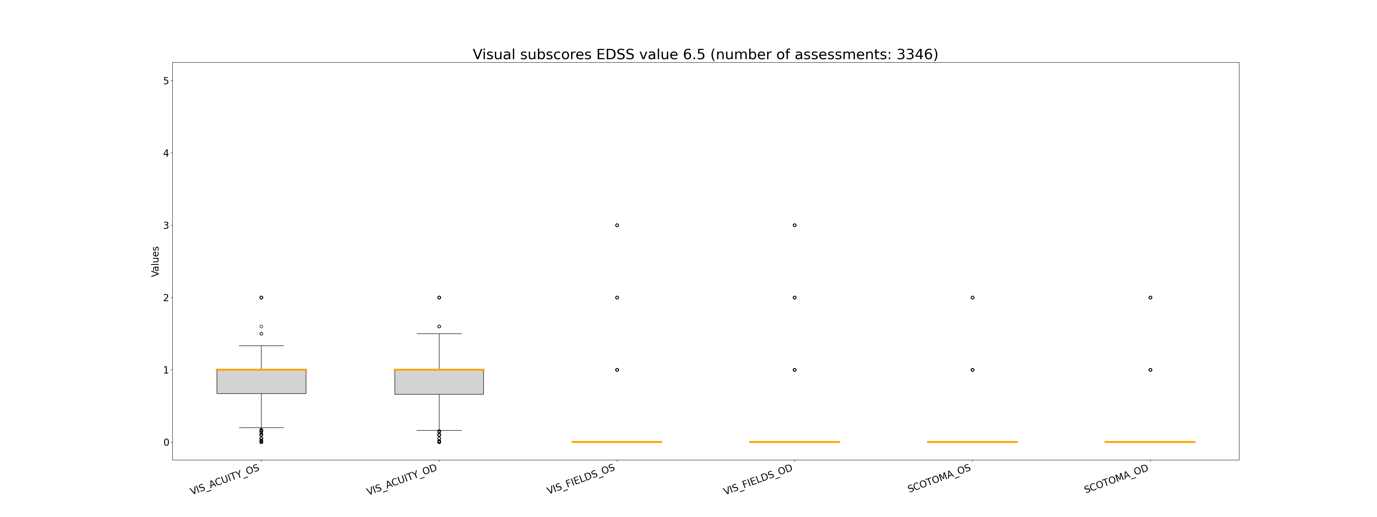

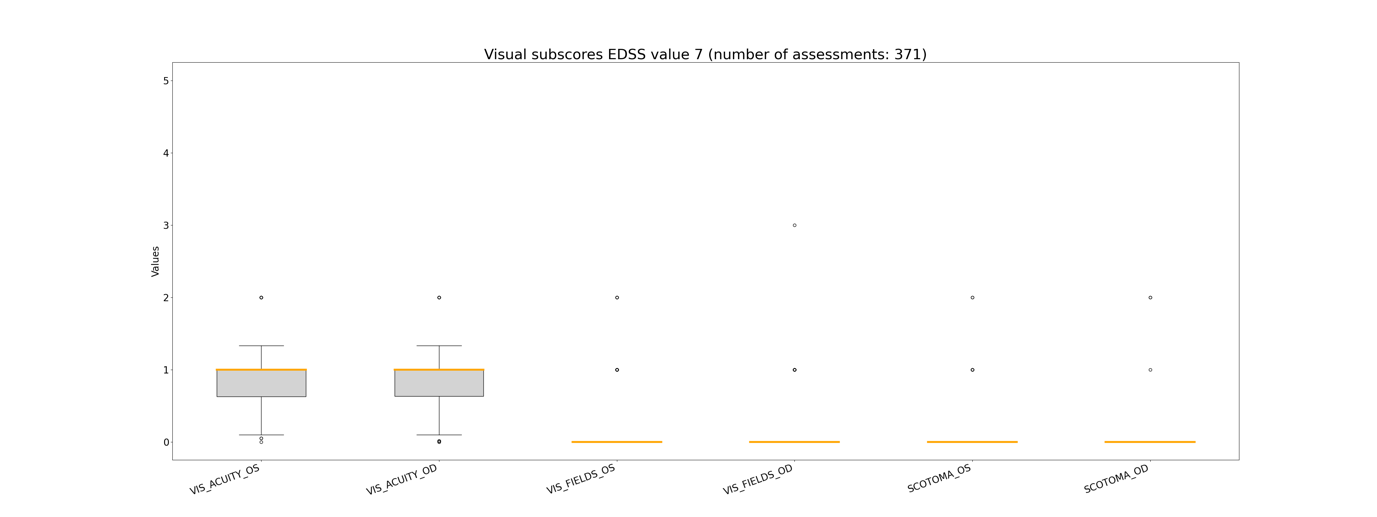

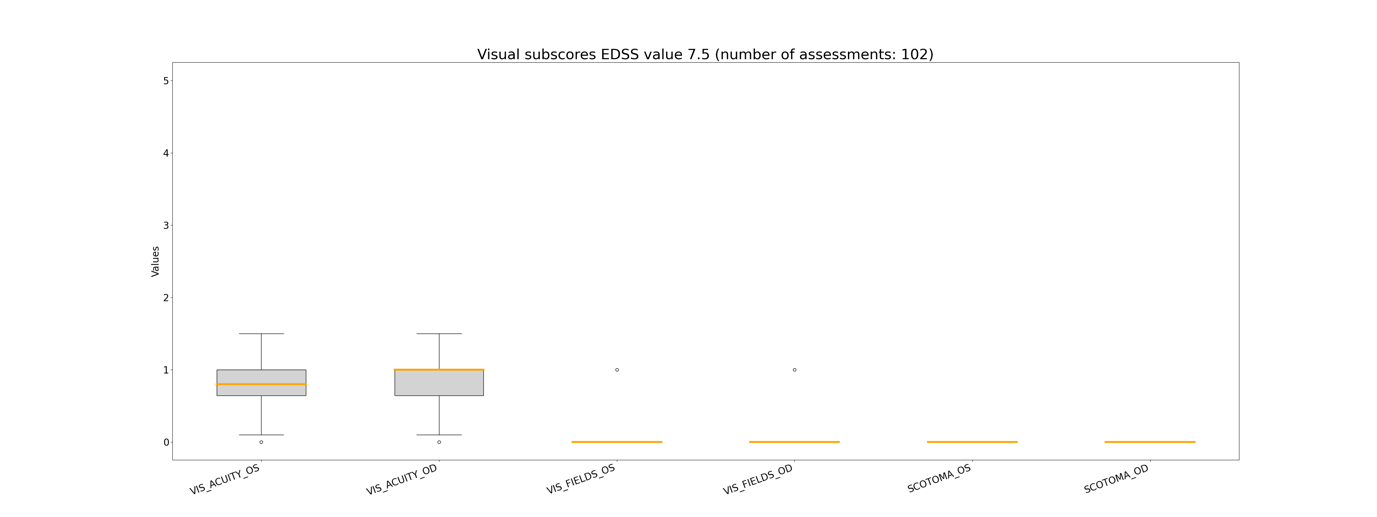

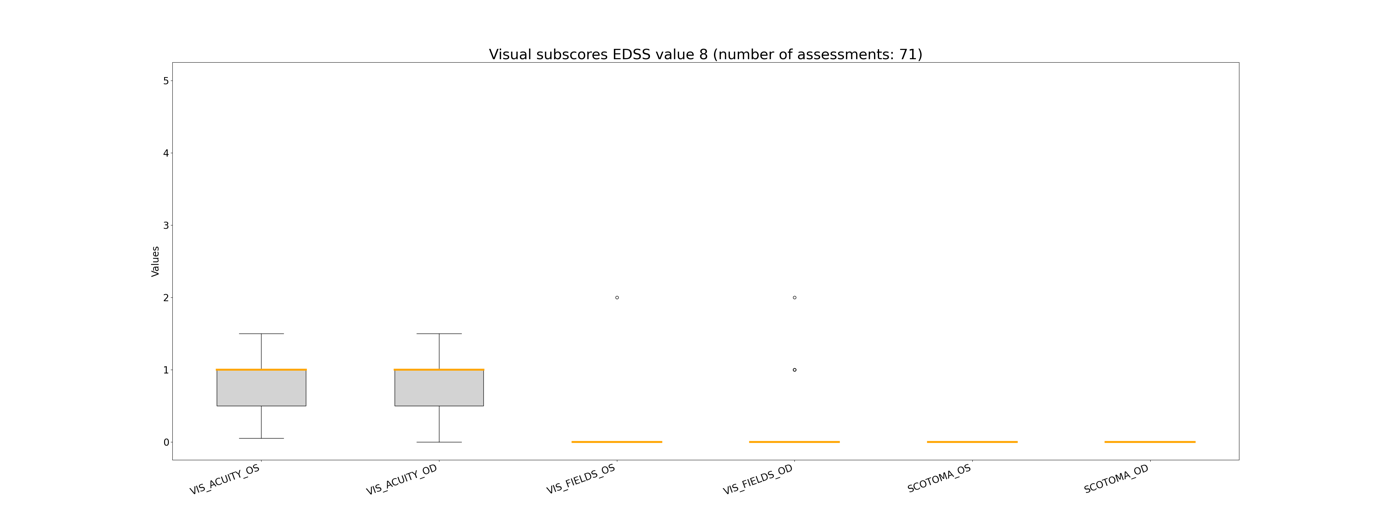

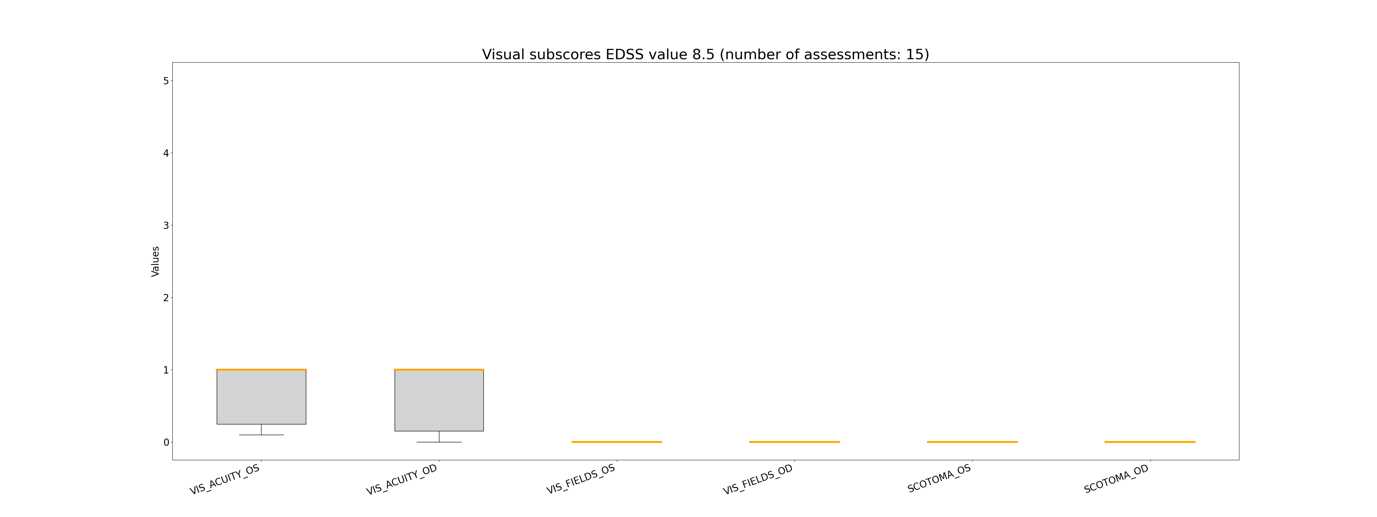


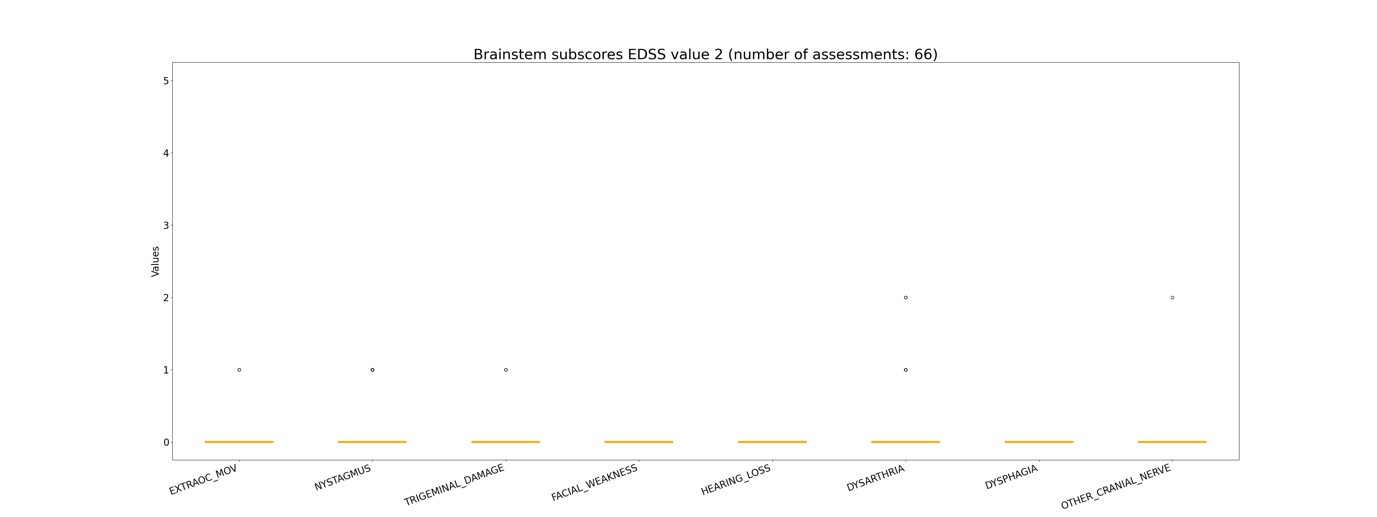

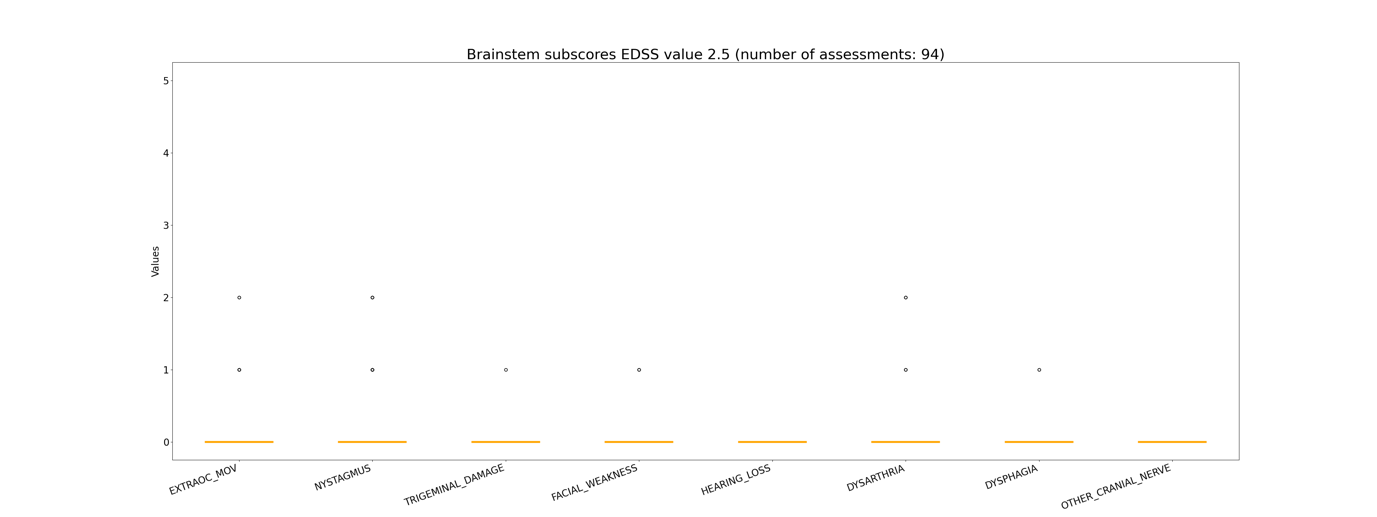

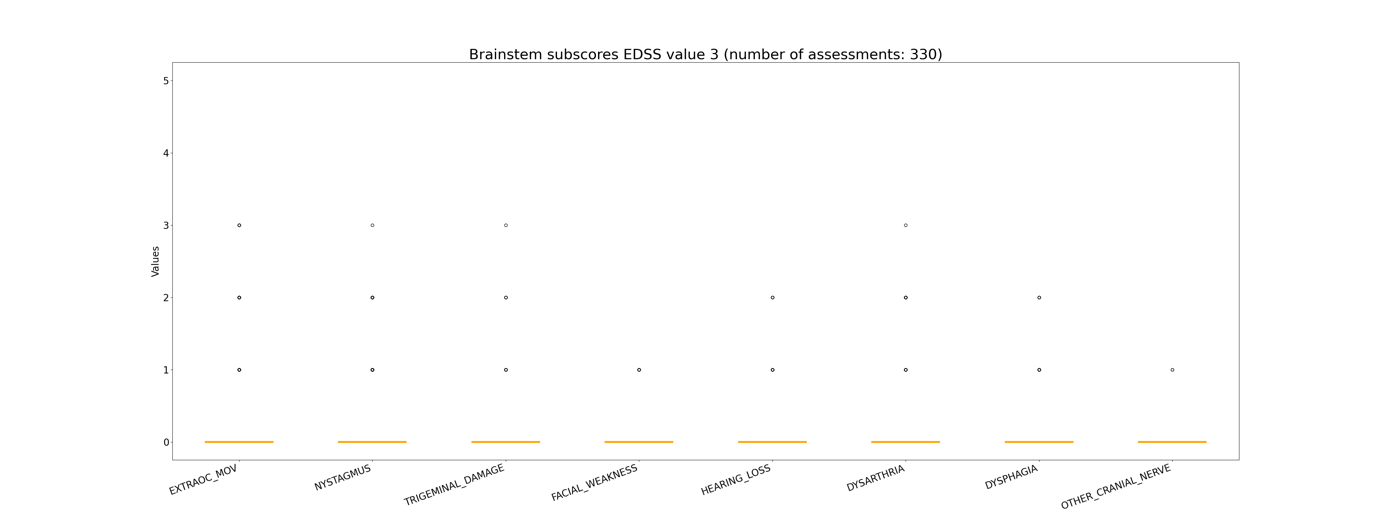


## 5.3 Summary of the abbreviations utilized in the boxplot

| **ABBREVIATIONS** | **FULL TERMS** |
| --- | --- |
| UE | UPPER EXTREMITIES |
| LE | LOWER EXTREMITIES |
| R | RIGHT |
| L | LEFT |
| VIS | VISUAL |
| EXTRAOC_MOV | EXTRAOCULAR MOVEMENTS (EOM) IMPAIRMENT |
| OTHER_CRANIAL_NERVE | OTHER CRANIAL NERVE FUNCTIONS |
| BMRC_DELTOID_R | LIMB STRENGTH: DELTOID_R |
| BMRC_DELTOID_L | LIMB STRENGTH: DELTOID_L |
| BMRC_BICEPS_R | LIMB STRENGTH: BICEPS_R |
| BMRC_BICEPS_L | LIMB STRENGTH: BICEPS_L |
| BMRC_TRICEPS_R | LIMB STRENGTH: TRICEPS_R |
| BMRC_TRICEPS_L | LIMB STRENGTH: TRICEPS_L |
| BMRC_W_F_FLEXOR_R | LIMB STRENGTH: WRIST/FINGER FLEXORS_R |
| BMRC_W_F_FLEXOR_L | LIMB STRENGTH: WRIST/FINGER FLEXORS_L |
| BMRC_W_F_EXTEN_R | LIMB STRENGTH: WRIST/FINGER EXTENSORS_R |
| BMRC_W_F_EXTEN_L | LIMB STRENGTH: WRIST/FINGER EXTENSORS_L |
| BMRC_HIP_FLEXORS_R | LIMB STRENGTH: HIP FLEXORS_R |
| BMRC_HIP_FLEXORS_L | LIMB STRENGTH: HIP FLEXORS_L |
| BMRC_KNEE_FLEXORS_R | LIMB STRENGTH: KNEE FLEXORS_R |
| BMRC_KNEE_FLEXORS_L | LIMB STRENGTH: KNEE FLEXORS_L |
| BMRC_KN_EXTEN_R | LIMB STRENGTH: KNEE EXTENSORS_R |
| BMRC_KN_EXTEN_L | LIMB STRENGTH: KNEE EXTENSORS_L |
| BMRC_PLT_FLEX_R | LIMB STRENGTH: PLANAR FLEXION_R |
| BMRC_PLT_FLEX_L | LIMB STRENGTH: PLANAR FLEXION_L |
| BMRC_DORSIFLEXION_R | LIMB STRENGTH: DORSIFLEXION_R |
| BMRC_DORSIFLEXION_L | LIMB STRENGTH: DORSIFLEXION_L |
| OVERALL_MOTOR_PERF | OVERALL MOTOR PERFORMANCE |
| TREMOR_DYS_UE_R | TREMOR/DYSMETRIA_UE_R |
| TREMOR_DYS_UE_L | TREMOR/DYSMETRIA_UE_L |
| TREMOR_DYS_LE_R | TREMOR/DYSMETRIA_LE_R |
| TREMOR_DYS_LE_L | TREMOR/DYSMETRIA_LE_L |
| RAPID_ALTER_MVT_UE_IMPAIR_R | RAPID ALTERNATING MOVEMENTS_UE_IMPAIRMENT_R |
| RAPID_ALTER_MVT_UE_IMPAIR_L | RAPID ALTERNATING MOVEMENTS_UE_IMPAIRMENT_L |
| RAPID_ALTER_MVT_LE_IMPAIR_R | RAPID ALTERNATING MOVEMENTS_LE_IMPAIRMENT_R |
| RAPID_ALTER_MVT_LE_IMPAIR_L | RAPID ALTERNATING MOVEMENTS_LE_IMPAIRMENT_L |
| OTHER__E__G__REBOUND | OTHER, E.G. REBOUND |
| SUPERFICIAL_SENS_UE_R | SUPERFICIAL SENSATION_UE_R |
| SUPERFICIAL_SENS_UE_L | SUPERFICIAL SENSATION_UE_L |
| SUPERFICIAL_SENS_TRUNK_R | SUPERFICIAL SENSATION_TRUNK_R |
| SUPERFICIAL_SENS_TRUNK_L | SUPERFICIAL SENSATION_TRUNK_L |
| SUPERFICIAL_SENS_LE_R | SUPERFICIAL SENSATION_LE_R |
| SUPERFICIAL_SENS_LE_L | SUPERFICIAL SENSATION_LE_L |
| URINARY_HES_AND_RET | URINARY HESITANCY/RETENTION |
| URINARY_URG_AND_INC | URINARY URGENCY/INCONTINENCE |
| BLADDER_CATHE | BLADDER CATHETERISATION |
| BOWEL_DYSF | BOWEL DYSFUNCTION |
| REFL | REFLEXES |

## 6. Histogram of the Dataset with EDSS between 4.0 and 6.5

The presented histograms illustrate the distribution of each FSS within the dataset composed by EDSS between 4.0 and 6.5. The histogram in blue represents the distribution of FSS selected through the feature selection process, while the grey plot corresponds to those that were excluded. The visual FSS correspond to the converted value of the visual FSS. The same applies to the bowel and bladder measurements.

### 7. Functional System score (FSS) level

To cluster groups with similar characteristics differently from the pattern score, we establish the FSS level. All the clusters that show a percentage of assessment higher than 50% with a high impact on ADLs in at least one of the Pyramidal subscores (BMRC, Spasticity) are classified as part of the Pyramidal level. Furthermore, if other subscores belonging to Cerebellar FS or Sensory FS are higher than the threshold, the cluster would belong to the level ‘Pyramidal Cerebellar’ or ‘Pyramidal Sensory’ respectively. This procedure was applied for all the combinations of FSS as reported in Table S3.

| **FSS**  **level** | **BMRC**  (upper,  lower) | **Spasticity**  (upper,  lower) | **Tremor**  (upper,  lower) | **Truncal ataxia** | **Tandem walking** | **Romberg text** | **Gait ataxia** | **Superficial Sens.**  (upper, lower) | **Vibration Sens.**  (upper,  lower) | **Position Sense**  (upper,  lower) |
| --- | --- | --- | --- | --- | --- | --- | --- | --- | --- | --- |
| **Pyramidal Cerebellar**  **Sensory** |  |  |  |  |  |  |  |  |  |  |
| **Pyramidal**  **Cerebellar** |  |  |  |  |  |  |  |  |  |  |
| **Pyramidal**  **Sensory** |  |  |  |  |  |  |  |  |  |  |
| **Pyramidal** |  |  |  |  |  |  |  |  |  |  |
| **Cerebellar**  **Sensory** |  |  |  |  |  |  |  |  |  |  |
| **Cerebellar** |  |  |  |  |  |  |  |  |  |  |
| **Sensory** |  |  |  |  |  |  |  |  |  |  |
| **None** |  |  |  |  |  |  |  |  |  |  |

Table S3: Description of the FSS level. For each row, the FSS level is defined if at least one for each colour category has a percentage of assessment with high impairment in daily life higher than 50%.

Table S4 presents the number of assessments and clusters belonging to clusters categorized under different FSS levels. It is apparent that as the EDSS step rises, the proportion of assessments indicating high impact on ADLs in Sensory FS diminishes. Conversely, there is an increase in the percentage of assessments demonstrating high disability in Pyramidal FS, Cerebellar FS, and in the category that includes all the FSS. This analysis underscores the heterogeneity among assessments classified under the same EDSS, presenting Top of Formdisability in diverse combinations of FSS.

| **EDSS** | **None** | **Pyramidal** | **Pyramidal**  **Cerebellar** | **Pyramidal**  **Sensory** | **Cerebellar** | **Cerebellar**  **Sensory** | **Sensory** | **Pyramidal**  **Cerebellar**  **Sensory** |
| --- | --- | --- | --- | --- | --- | --- | --- | --- |
| **4.0** | 26.3% | 11.3% | 16.3% | 2.3% | 20.3% | 8.8% | 10.8% | 3.9% |
| **4.5** | 20.1% | 18.3% | 23.7% | 4.1% | 20.8% | 5.2% | 5.5% | 2.2% |
| **5.0** | 20.4% | 21.3% | 23.4% | 7.1% | 18.4% | 0.0% | 6.0% | 3.4% |
| **5.5** | 12.8% | 17.6% | 27.0% | 3.1% | 14.0% | 6.8% | 5.6% | 13.1% |
| **6.0** | 4.6% | 11.4% | 48.4% | 0.0% | 19.3% | 6.0% | 0.0% | 10.2% |
| **6.5** | 0.0% | 4.5% | 85.4% | 0.0% | 4.3% | 0.0% | 0.0% | 5.8% |

Table S4: Results reported the percentage of assessments in each Disability level.
